# Integrated UPLC-MS and Network Pharmacology Approach to Explore the Active Components and the Potential Mechanism of Yiqi Huoxue Decoction for Treating Nephrotic Syndrome

**DOI:** 10.3389/fphar.2021.775745

**Published:** 2022-02-24

**Authors:** Dan Feng, Xiang-Ri Li, Zhao-Yi Wang, Nian-Nian Gu, Shuang-Xi Zhang, Chao-Feng Li, Yang Chen, Zhi-Qiang Ma, Rui-Chao Lin, Hong-Gui Zhang, Chongjun Zhao

**Affiliations:** ^1^ School of Chinese Materia Medica, Beijing University of Chinese Medicine, Beijing, China; ^2^ First Affiliated Hospital of Henan University of Traditional Chinese Medicine, Zhengzhou, China

**Keywords:** Yiqi huoxue decoction, ultra-high performance liquid chromatograph-mass, network pharmacology, component, nephrotic syndrome, mouse podocyte clone-5, phosphatidylinositol 3 kinase-RAC serine/threonine-protein kinase, nuclear factor kappa-B

## Abstract

**Background:** Yiqi Huoxue Decoction (YQHXD) is a traditional Chinese medicine that promotes blood circulation, removes blood stasis, facilitates diuresis, and alleviates edema. It is composed of 10 herbal medicines and has extensive application in treating nephrotic syndrome (NS). However, the active components and the potential mechanism of YQHXD for treating NS remain unclear.

**Methods:** We set up a sensitive and rapid method based on Ultra-High Performance Liquid Chromatograph-Mass (UPLC-MS) to identify the compounds in YQHXD and constituents absorbed into the blood. Disease genes were collected through GeneCards, DisGeNET, and OMIM database. Genes of compounds absorbed into blood were predicted by the TCMSP database. We constructed Disease-Drug-Ingredient-Gene (DDIG) network using Cytoscape, established a Protein-protein interaction (PPI) network using String, Gene biological process (GO), and Kyoto Encyclopedia of Genes and Genomes (KEGG) pathway analysis was performed using DAVID. Cellular experiments were performed to validate the results of network pharmacology.

**Result:** A total of 233 compounds in YQHXD and 50 constituents absorbed into the blood of rats were identified. The 36 core targets in the PPI network were clustered in the phosphatidylinositol 3 kinase-RAC serine/threonine-protein kinase (PI3K-AKT) and nuclear factor kappa-B (NF-κB) signaling pathways. Luteolin, Wogonin, Formononetin, and Calycosin were top-ranking components as potentially active compounds.

**Conclusion:** The results of our studies show that YQHXD is able to enhance renal function, alleviate podocyte injury, and improve adriamycin nephrotic syndrome.

## Introduction

Nephrotic syndrome (NS) is a kidney disease with a complicated mechanism, characterized by symptoms such as proteinuria, edema, hyperlipidemia, and hypoalbuminemia (Wang and Greenbaum 2019). It is classified into different pathological types due to the damage of glomerular capillary filtration membrane by numerous factors (Yizhi et al., 2014). Multiple pathogenesis results in complicated clinical manifestations and unpredictable disease progression. The incidence of end-stage renal disease (ESRD) has exceeded 30% among patients without effective treatment. Long-term treatment of immunosuppressants and glucocorticoids results in immune dysfunction or complex infections in patients (Keri et al., 2019). Therefore, treatment for NS requires further exploration to address the current issues.

Yiqi Huoxue Decoction (YQHXD) is composed of Astragalus mongholicus Bunge (AMB), Salvia miltiorrhiza Bunge (SMB), Codonopsis pilosula (Franch.) Nannf (CPN), Atractylodes macrocephala Koidz (AMK), Wolfiporia extensa (Peck) Ginns [syn. Poria cocos (Schw.)] (WEG), Leonurus japonicus Houtt (LJH), Plantago asiatica L. (PSL), Angelica sinensis (Oliv.) Diels (ASD), Ligusticum striatum DC. (syn. Ligusticum wallichii Franch.) (LSD), and Paeonia lactiflora Pall (PLP). The decoction can fortify the kidney and spleen, invigorate the blood, and remove stasis, which achieved well-recognized efficiency in treating nephrotic syndrome in clinical practice for decades. In this prescription, AMB, CPN, AMK, and WEG fortify the spleen and nourish the qi, PSL relieves swelling, and SMB, ASD, PLP, LSD, and LJH activate the blood and resolve stasis. The combined use of ten herbal medicines strengthens the immune system, lowers blood lipids, prevents inflammation and oxidation, and boosts energy.

Multiple compounds in YQHXD have been proved to be effective in the treatment of kidney diseases. SUN et al. revealed that the human mesangial cell line (HMC) exposure to high glucose downregulated the expression of TRPC6 protein, and induced the contractile dysfunction of mesangial cells. But treated with Astragaloside IV, HMC inhibited HG-induced contractile dysfunction and mesangial cell proliferation through the NADPH oxidase/ROS/Akt/NF-κB signaling pathway. Rats were injected with adriamycin (7.5 mg/kg) once to induce kidney injury, after administering with Salvianolic acid A (SAA) and prednisone acetate for 21 days, the symptoms of adriamycin-induced nephropathy were significantly improved. Fan et al. demonstrated that SAA, as a multifunctional compound, ameliorated histological damages, alleviated proteinuria and hyperlipidemia, downregulated oxidative stress, relieved blood hypercoagulability (Fan et al., 2015). Furthermore, SAA up-regulated the expression of podocin and IκBα protein, down-regulated the expression of p-IκBα, NF-κB p65. Cheng et al. revealed the pretreatment of Leonurine before ureteral obstruction repealed the expression of fibronectin, down-regulated the expression of vimentin, α-SMA, and type I/III collagen, TGF-β, TNF-α, IL-6 and IL-1β, Smad3, which revealed that Leonurine might be a renoprotective candidate for renal-fibrosis *via* effecting the NF-κB pathway and TGF-β/Smad3 pathway (Cheng et al., 2015). These studies laid a research foundation for multi-component and multi-target of YQHXD for treating nephrotic syndrome.

Investigating the effective material base of Chinese herbal composite is key to clarifying the compatibility mechanism of traditional Chinese medicine (TCM) prescriptions and improving the Chinese herbal medicines industry (Ma et al., 2017). Effective ingredients of TCM herbs are transported to organs, tissues, and target points after entering the blood, and a therapeutic effect on diseases is only achieved with a sufficient blood concentration (He et al., 2015). Therefore, the components that enter the blood may be the effective substances of the TCM decoction. The serum pharmacochemistry based on the determination of effective ingredients of TCM comprehensively analyzes these components which promotes the progression and development of the theory and practice of Chinese herbal medicines through the study on the dynamic characteristic of principal effective substances *in vivo* (Wang et al., 2005).

Ultra-performance liquid chromatography (UPLC) coupled with ESI-LTQ-Orbitrap XL mass spectrometry (MS) is a novel high-resolution mass spectrometry technology with high-resolution advantages, mass accuracy and wide dynamic range, which can quickly and comprehensively identify and analyze multi-component systems (Zubarev and Makarov 2013). The ESI-LTQ-Orbitrap XL mass analyzer potentiates the selectivity and confidence of routine analyses. The orbitrap provides a wide range of applications from routine component identification to the analysis of trace-level compounds in complicated mixtures, which has also contributed greatly to many discoveries in the field of TCM (Selliez et al., 2020).

Systems biology lays the foundation for the development of network pharmacology in TCM (Fang et al., 2013). The prediction of potential active ingredients and targets of herbal medicines provides a theoretical basis for animal or cellular experiments, which facilitates the rapid and accurate interpretation of the compounding rules of herbal medicines (Li and Zhang 2013). The multi-targeted action relationships of complex components of herbal medicines, interconnected by networks, can reveal the potential action pathways of herbal medicines for the treatment of diseases after high-throughput computational analysis (Hao da and Xiao 2014). The research idea of network pharmacology is consistent with the “holistic view” of TCM for disease treatment (Li et al., 2012), so network pharmacology provides a new direction for TCM modernization research by investigating the potential protein targets of TCM for disease treatment and elucidating the compounding rules (Luo et al., 2020).

Podocytes are cells with complex morphology within the glomerulus that cover the outer surface of the filtering capillaries, maintain the normal morphological structure of the glomerular basement membrane (GBM), construct the glomerular filtration barrier, and regulate glomerular filtration. Podocyte injury indicates glomerular disease. The production of reactive oxygen species (ROS) and inflammatory factors induced by adriamycin impairs the normal structure and function of podocytes. The adriamycin-induced pathological model of mouse podocyte clone-5 (MPC5) cells has been widely used to study the pathological manifestations and mechanisms of nephrotic syndrome *in vitro*.

The current study elucidated the material basis and pharmacological mechanism of YQHXD in the treatment of nephrotic syndrome by UPLC-MS and network pharmacology for the first time to provide a basis for further research and clinical application of YQHXD against nephrotic syndrome.

## Experiments

### Materials and Reagents

4-hydroxycoumarin, Ferulic acid, Isoquercitrin, Chlorogenic acid, Gallic acid, Tormentic acid, Leucine, Wogonin, Arginine, Levistilide A, Quercetin, Leonurine, L-Isoleucine, Apigenin, Isoquercitrin, Danshensu, Cryptotanshinone, Hyperoside, Salicylic acid, Guanosine, Rutin, Tyrosine, Astragaloside IV, Calycosin-7-O-glucoside, Atractylenolide I, Valine, Protocatechuic acid, Phenylalanine, Atractylenolide Ⅱ, Adriamycin, Luteolin, Calycosin, Formononetin were purchased from Beijing Innochem Technology Co., Ltd. (Beijing, China). The purities of all of the standard compouds are no less than 98% based on HPLC analysis by normalisation of the peak areas. MPC5 cells were purchased from BeNa Culture Collection. AMB, SMB, CPN, AMK, WEG, LJH, PSL, ASD, LSD, PLP were purchased from Beijing University of Chinese medicine third affiliated hospital. Analytical grade of methanol, chloroform, ethanol was purchased from Beijing Chemical Industry Co., Ltd. (Beijing, China). HPLC grade of formic acid and methanol, mass spectrometry grade of acetonitrile were purchased from Thermo Fisher Scientific Co., Ltd. (America). Deionised water was purified by a Milli-Q system (Millipore, Bedford, Massachusetts, America); Microsample total RNA Extraction Kit (Tiangen Biochemical Technology Co., Ltd., China); TransScript^®^ One step gDNA removal and cDNA synthesis Supermix (Beijing all gold Biotechnology Co., Ltd., China); Protein quantification (TP) assay kit, Superoxide dismutase (SOD) kit, Catalase (CAT) kit, Malondialdehyde (MDA), Glutathione peroxidase (GSH-Px) kit, Caspase 3 kit and Caspase 9 kit were obtained from Nanjing Jiancheng Bioengineering Institute (Nanjing, China).

### Studies on the Chemical Constituents of YQHXD

#### Preparation of YQHXD Test Solution and Standard Solutions

YQHXD consists of ten herbs, including 60 g AMB, 30 g SMB, 30 g CPN, 30 g AMK, 30 g WEG, 30 g LJH, 15 g PSL, 15 g ASD, 10 g LSD, 10 g PLP. After being soaked with distilled water for 30 min, the materials of YQHXD were decocted twice for 2 h each time. Then the supernatants were collected, concentrated, evaporated to dryness by rotary evaporator, and the rate of aqueous extract was 22.93%. The aqueous extract was freeze-dried with a low-temperature freeze dryer, and the yield of the lyophilized powder was 13.81%.

1.0 g lyophilized powder was dissolved in 70% methanol (20 ml) and ultrasonic extracted for 40 min. The solution was centrifugated at 4,000 rpm for 15 min, and test solution was obtained after filtering the supernatant through a 0.22 μm membrane. Standard solutions were dissolved in 70% methanol. All solutions were stored at 4°C.

#### UPLC-ESI-LTQ-Orbitrap XL Condition

Chromatographic separation was performed on a Waters ACQUITY UPLC HSS T3 C18 column (100 mm × 2.1 mm, 1.8 μm) at a flow rate of 0.3 ml/min at 25°C. The injection volume was 5 μL. The mobile phase contained acetonitrile (C) and 0.1% formic acid aqueous solution (B). The chromatographic separation was performed by the following gradient elution program: 0–2 min, 2–5% C; 2–2.5 min, 5–13% C; 2.5–16 min, 13–36.5% C; 16–18 min, 36.5–60% C; 18–27 min: 60–80% C; 27–30 min: 80–98% C; 30–35 min: 98% C.

The analysis of components and serum pharmacochemistry of YQHXD were performed with Dionex UltiMate 3000 ultra-high performance liquid chromatography (UPLC) coupled with an LTQ-Orbitrap XL MS that was equipped with positive and negative ion mode of ESI source, with the capillary voltages of 25 V (ESI^+^) and 35 V (ESI^−^), and the spray and tube lens voltages of 4.0 and 110.0 V respectively. Nitrogen was used as the auxiliary gas and sheath gas, with the flow rates seperately set at 20 arb and 40 arb. The primary mass spectrometer conducted a full scan with a resolution of 30000 and a mass range of m/z 100-1,200. In this experiment, data-dependent scanning was adopted to strike MS^2^ fragmentation and MS^3^ fragmentation, and in each scanning point, the strongest three-parent ions were used as targeted precursor ions for further fragmentation. Data were collected by a high-resolution Fourier transform mass spectrometer and an ion trap. Xcalibur software was employed to process the experimental data.

#### Qualitative Identification Method

Literature on the chemical composition of Chinese herbal medicines of YQHXD in the following databases: CNKI (https://www.cnki.net), PubMed (https://pubmed.ncbi.nlm.nih.gov/) and Web of Science (http://apps.webofknowledge.com), was searched and listed in the self-built database. The name, molecular formula, molecular weight and chemical structure of the compounds were then completed by searching the following databases: ChemSpider (http://www.chemspider.com/), and Chemicalbook (https://www.chemicalbook.com).

In the workstation, a formula predictor was used to generate the formulas of parent ions according to their accurate mass. The maximal mass error was set to ±5 ppm, with the maximum element composition set to (C = 50, H = 100, O = 30, N = 5, S = 4). The types of compounds were predicted through analysis of retention time, molecular formula, chromatographic behavior, secondary (tertiary) mass spectrometry fragments, and the data in references, self-built databases and online mass spectrometry databases, such as Massbank (https://massbank.eu/MassBank), PubChem (https://www.ncbi.nlm.nih.gov/pccompound), and Mzcloud (https://www.mzcloud.org/). They were compared with standard products or literature for confirmation after narrowing the scope to obtain several target compounds with higher probability.

### Studies on the Chemical Constituents Absorbed in the Blood

Male Sprague Dawley rats (weighing 200 ± 20 g) were purchased from SPF (Beijing) Biotechnology Co., Ltd. [License number: ICP (Beijing) 20001007, Beijing, China]. Thirty rats were housed in the animal room at the Beijing University of Chinese Medicine. The animal experiment was started after 5 days of adaptive feeding. Rats were provided with a standard diet and water, and every six rats were assigned to one group in one cage. All experiments were conducted in accordance with the Regulations of Experimental Animal Administration issued by the State Commission of Science and Technology of the People’s Republic of China. All procedures of animal care and animal experiments were authorized by the Animal Ethical and Welfare Committee of Beijing University of Chinese Medicine (BUCM: 4-2020062201-2018).

The rats were administered with lyophilized powder of YQHXD dissolved in deionized water at a dose of 3.24 g/kg twice a day for 3 consecutive days. At the end of the third day, the rats were fasted but had free access to water for 12 h, on the fourth-day morning, 30 min after administration, blood samples (300 μL) were collected from the orbital venous plexus, transferred into heparinized 1.5 ml EP centrifuge tubes, and centrifuged at 4,000 rpm for 10 min to collect the supernatant which was then stored at −80°C.

200 μL of plasma and 1 ml of methanol were added into a 1.5 ml Eppendorf tube and vortex mixed for 3 min. The mixture was subsequently centrifuged at 12,000 rpm for 15 min, and the supernatant was transferred into a new 1.5 ml Eppendorf tube and evaporated to dryness through nitrogen at 35°C. The residue was reconstituted in 100 μL methanol and centrifuged at 12,000 rpm for 15 min, and 5 μL of the supernatant was injected into the UPLC-ESI-LTQ-Orbitrap XL/MS system for analysis. The gradient elution procedure is the same as that of *UPLC-ESI-LTQ-Orbitrap XL Condition*.

### Network Pharmacology

#### Target Collection of NS and Components

The retrieved keyword was “nephrotic syndrome,” and gene targets of NS were collected through retrieving the three following sources: GeneCards database (https://www.genecards.org), DisGeNET database (https://www.disgenet.org), OMIM database (http://omim.org). After deleting the duplicate targets and eliminating the redundant targets, the integrated targets of three databases were obtained. The targets of components absorbed in the rats’ blood were examined by the TCMSP database.

#### Network Construction

Cytoscape software was used to construct the Disease-Drug-Ingredient-Gene interaction network (DDIG), and the “Network Analyzer” was employed to analyze the network topology to further screen out the top 10 active ingredients and the top 15 target genes.

#### Protein-Protein Interaction Network

The 79 intersectional targets were imported into the String database (https://string-db.org) to build the interactional network for YQHXD component targets and nephrotic syndrome related targets. A high confidence score of 0.7 was used to screen the interactional targets with the highest correlation. Then the targets from the String database were imported into Cytoscape. According to the results of NetworkAnalyzer, 36 targets higher than the average degree were considered as the core targets and constructed PPI network.

#### GO Enrichment Analysis and KEGG Enrichment Analysis

The annotation and signaling pathways of the 36 core targets were conducted by Gene biological process (GO), and Kyoto Encyclopedia of Genes and Genomes (KEGG) enrichment analysis in the DAVID database (https://David.Ncifcrf.Gov/summary.JSP), and the first 20 items were filtered according to the fold enrichment value to manifest the signaling pathway and mechanism of YQHXD in the treatment of nephrotic syndrome.

### Cellular Experiments

#### Cell Culture

MPC5 cells were cultured in a 1640 medium containing fetal bovine serum and penicillin/streptomycin at 37°C in a 5% CO_2_ incubator, and the culture medium was refreshed daily.

#### MTT for Cell Viability

Exposure concentrations: MPC5 cells were exposed to adriamycin at 4, 2, 1, 0.5, 0.25, 0.125, and 0.0625 μM, YQHXD extracts at 3,200, 1,600, 800, 400, 200, 100, 50, 25, 12.5, and 6.25 μg/ml, and Luteolin, Wogonin, Formononetin, and Calycosin at 320, 160, 80, 40, 20, 10, 5, 2.5, 1.25, and 0.625 μM. Digestion was performed when the cell density reached 70% and a cell suspension was prepared at a concentration of 3.0 × 10^4^ cells/mL, followed by the inoculation into a 96-well plate and 24 h cultivation at 37°C in a 5% CO_2_ incubator. Subsequently, each well was added with 100 μL of different concentrations of Adriamycin, YQHXD extract, Luteolin, Wogonin, Formononetin, and Calycosin, and the culture solution was discarded after 48 h. 100 μL of medium and 10 μL of MTT solution at a concentration of 5 mg/ml were added to each well and incubated for 4 h. The solution in the wells was then discarded, and added with DMSO at 150 μL per well and mixed well. The optical density (OD) value was measured at 490 nm.

Cell viability% = (OD _value of compound group_/OD _value of the control group_).

#### Cell Grouping and Administration

5 ml of cell suspension at a concentration of 3.0 × 10^5^ cells/mL was inoculated in cell culture dishes. Blank control: Cells were exposed to the culture medium. Model group: Cells were exposed to 0.5 μM adriamycin. YQHXD extract high, medium, and low dose groups: Cells were exposed to 0.5 μM DOX + 200 μg/ml YQHXD, 0.5 μM DOX + 100 μg/ml YQHXD, and 0.5 μM DOX + 50 μg/ml YQHXD, respectively. Luteolin high, medium, and low dose groups: Cells were exposed to 0.5 μM DOX + 20 μM LUT, 0.5 μM DOX + 10 μM LUT, 0.5 μM DOX + 5 μM LUT. Formononetin high, medium, and low dose groups: Cells were exposed to 0.5 μM DOX + 20 μM FOR, 0.5 μM DOX + 10 μM FOR, and 0.5 μM DOX + 5 μM FOR, respectively. Wogonin high, medium, and low dose groups: Cells were exposed to 0.5 μM DOX + 40 μM WOG, 0.5 μM DOX + 20 μM WOG, and 0.5 μM DOX + 10 μM WOG, respectively. Calycosin high, medium, and low dose groups: Cells were exposed to 0.5 μM DOX + 40 μM CAL, 0.5 μM DOX + 20 μM CAL, and 0.5 μM DOX + 10 μM CAL, respectively.

#### Assay of SOD, CAT, MDA, GSH-Px

MPC5 cells were placed in the culture dishes for 24 h, compounds of different concentrations and YQHXD were added respectively. At 48 h, adriamycin was added to each group. At 72 h, cell lysates from different drug treatment groups were collected and the activities of superoxide dismutase (SOD), catalase (CAT), malondialdehyde (MDA), glutathione peroxidase (GSH-Px) were measured according to the instructions.

#### Detection of Caspase 3 and Caspase 9

MPC5 cells were placed in the culture dishes for 24 h, compounds of different concentrations and YQHXD were added respectively. At 48 h, adriamycin was added to each group. At 72 h, cell lysates from different drug treatment groups were collected and Caspase 3 and Caspase 9 expression levels were determined according to the instructions.

#### RT-qPCR Experiment

Total RNA was extracted from each group of cells using the cellular RNA extraction kits. The cDNA was synthesized using a one-step genomic cDNA first-strand synthesis Ultramix reagent, and the gene primers were synthesized by Beijing Bomad Biological Company. The mRNA was quantified using the PerfectStart Green qPCR SuperMix kit, with the β-actin gene as the internal reference. After the polymerase chain reaction (PCR) amplification, the average cycle threshold (CT) was calculated using “ABI Stepone Software V2.1” software, and the △CT value was calculated according to the formula △CT = CT _target gene_−CT _control gene_, and the results were analyzed using 2−^△△CT^ values (△△CT = △CT _target sample_−△CT _control sample_). The purity of RNA is shown in Table 5. The primer sequences are shown in Table 6.

## Results

### Identification of Chemical Components of YQHXD and Constituents Absorbed in Rat Plasma

UPLC-LTQ-Orbitrap/MS technology was adopted for the rapid separation, identification and structural analysis of chemical components in YQHXD and its absorbed constituents into the rats’ blood after oral administration of YQHXD. According to the accurate mass to charge ratio of the collected ions, the chemical components in YQHXD and rat plasma were identified through comparison with data of chemical components of YQHXD in the database.

**FIGURE 1:**
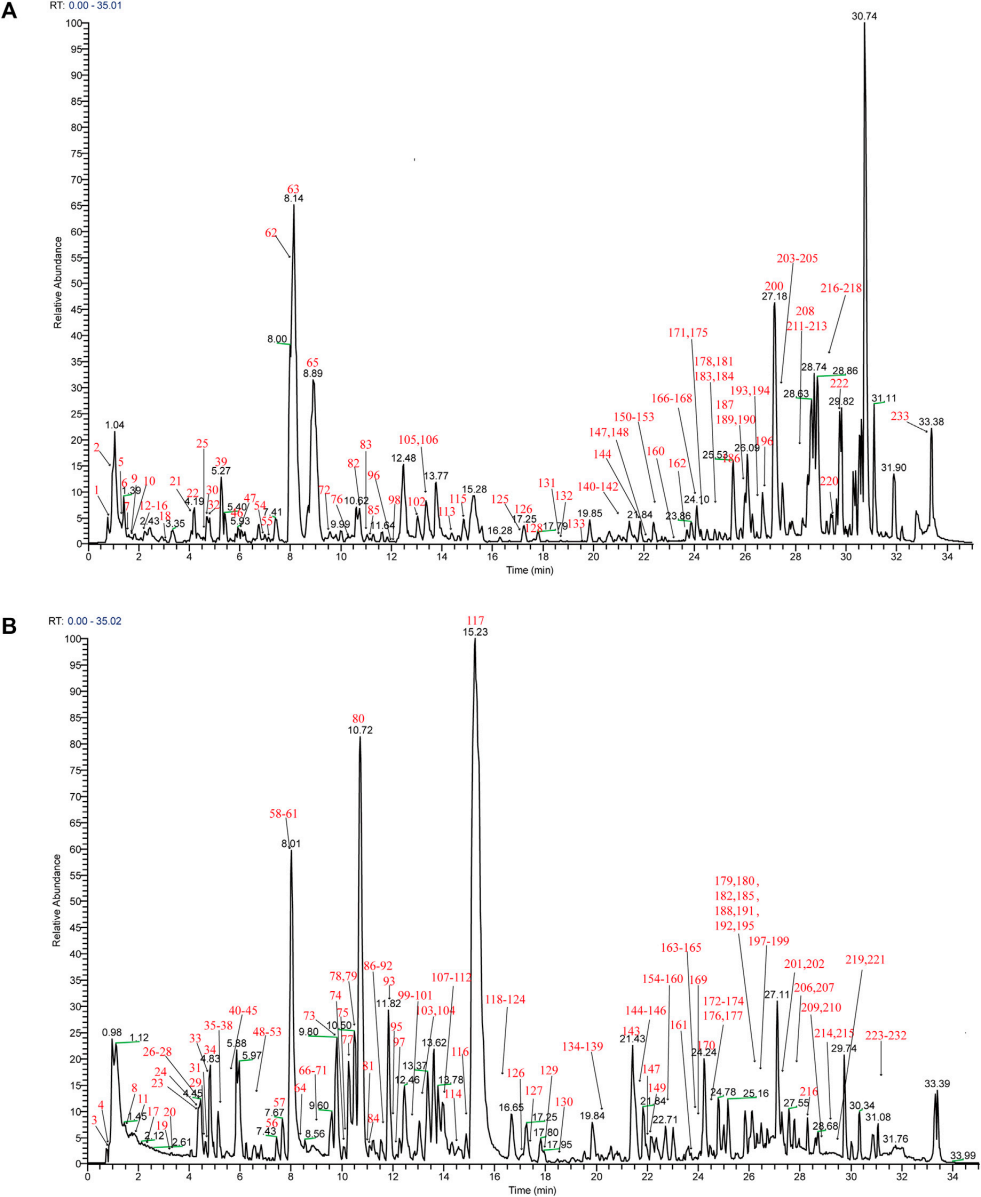
The total ion chromatogram of YQHXD. **(A)** The total ion chromatogram in positive ion mode of YQHXD; **(B)** The total ion chromatogram in negative ion mode of YQHXD.

**FIGURE 2:**
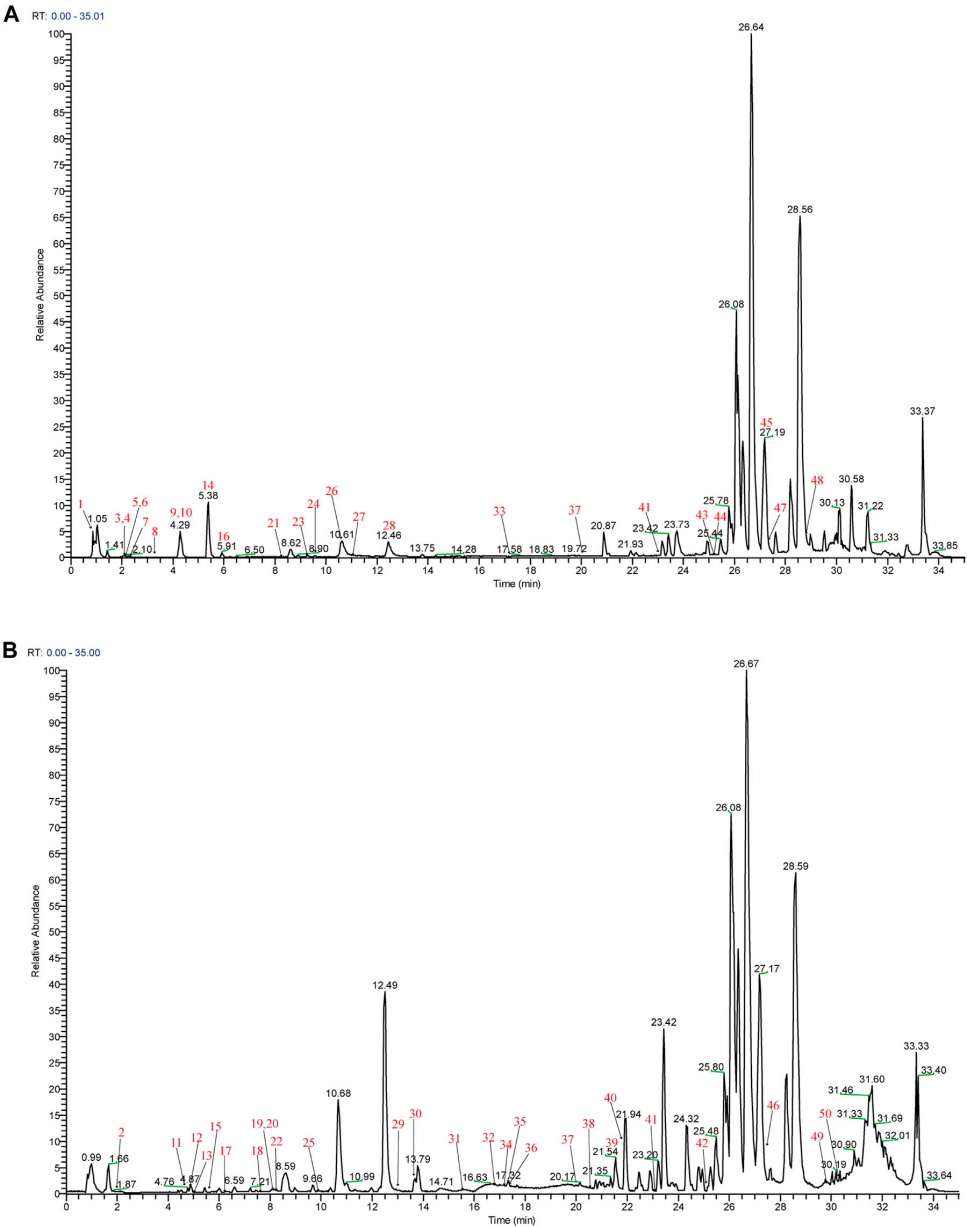
The total ion chromatogram of components absorbed into the blood of rats. **(A)** The total ion chromatogram in positive ion mode of components absorbed into the blood of rats; **(B)** The total ion chromatogram in negative ion mode of components absorbed into the blood of rats.

Mass spectra were obtained in negative and positive modes to better identify the compounds. Using the analysis and comparison of secondary fragment or tertiary fragment ions of specific compounds, a total of 233 detected chromatographic peaks were revealed in YQHXD (Figure 1) and 50 chromatographic peaks (Figure 2) were elucidated from the rats’ plasma *via* comparison with the accurate molecular mass, retention times, fragmentation pathways of constituents to be resolved with those of standard substances and in referenced literature. The identified results are shown in Supplementary Tables S1, S2.

In the negative ion mode, [M + COOH]^−^ and [M-H]^-^ were obtained, and [M + H]^+^, [M + Na]^+^, [2M + Na]^+^ were observed in the positive ion mode. Those molecular ion peaks facilitated the calculation of the molecular weights, and the MS/MS data were used for structures deduction. A total of 233 chemical constituents of YQHXD were identified and divided into 17 categories, namely, phenolic acids, flavonoids, diterpenes, triterpenes, monoterpene glycosides, phthalides, amino acids, sesquiterpene lactones, phenylethanoid glycosides, iridoid glycosides, nucleotides, fatty esters, aromatic esters, saponins, coumarins, fatty acids, and other compounds.

#### Mass Fragmentation Regularities of Compounds

##### Identification of Phenylethanoid Glycosides

The Phenylethanoid glycosides mainly came from Leonurus japonicus Houtt and Plantago asiatica L. Their structures were characterized by a phenethyl alcohol (C_6_-C_2_) moiety linked to a β-allopyranose/β-glucopyranose via a glycosidic bond. Many substituents usually modified their structures, for example, aromatic acids: caffeic acid, ferulic acid through ester linkages and different saccharides: rhamnose, glucose through glycosidic linkages (Kirmizibekmez et al., 2005).

Forsythoside E was a phenylethanoid glycoside with two sugar groups linked by hydroxytyrosol; its characteristic fragment ions were at m/z 315.05771 [M-H-Rha]^−^, 152.86441 [M-H-Rha-Glc]^−^. Verbascoside, Isoverbascoside and Plantainoside D were compounds formed by sugar, phenyl ethanol and caffeic acid, they first eliminated the caffeoyl group and then lost the glycosyl group. Product ions at m/z 179, 161, 135 corresponding to the signal of caffeic acid, caffeic acid dehydration and caffeic acid decarbonization which formed after the parent ion desorbed caffeic acid acyl group (Jiang et al., 2009). In the negative mass spectrum of Plantainoside D, its parent ion was at m/z 639.19196 [M-H]^−^, the ion m/z 160.89789 indicated the elimination of glucose and the continuous loss of water molecules. Simultaneously, the product ions at m/z 477.22302 [M-H-C_9_H_6_O_3_]^−^, m/z 315.16354 [M-H-C_9_H_6_O_3_-Glc]^−^, m/z 134.88246 [M-H-C_9_H_6_O_3_-2Glc-H_2_O]^−^ were inferred to the consecutive loss of caffeoyl group, glycosyl group, and water molecule (Manyuan et al., 2014). Similarly, the parent ion at m/z 623.19704 [M-H]^−^ of Verbascoside consecutively removed caffeoyl group and glycosyl group and water molecule to obtain characteristic ion fragments at m/z 461.21570 [M-H-C_9_H_6_O_3_]^−^, m/z 315.22729 [M-H-C_9_H_6_O_3_-Rha]^−^.

##### Identification of Flavonoids

The fragmentation pattern of flavonoids by mass spectrometry has been extensively studied. Most flavonoids had hydroxy phenol, methylated phenol and glycoside groups. Flavonoid glycosides were easy to lose a glycoside group (162 Da), malonylglucosyl group (248 Da), or acetylglucosyl group (204 Da). The loss of small neutral molecules [CH_3_(15 Da), H_2_O (18 Da), CO (28 Da), and CO_2_ (44 Da)] usually occurred in the mass spectra of flavonoids. In the negative ion mode, the fragment ions formed by the RDA cleavage of C-ring of flavonoids were ^1,3^A^−^, ^1,3^B^−^ respectively. For example, the RDA cleavage of apigenin produced product ions at m/z 150.74980 [M-H-C_8_H_6_O]^−^ and 116.85899 [M-H-C_7_H_4_O_4_]^−^. Due to the existence of the 3-hydroxyl group, flavonol glycosides often formed the fragmentation ions [Y_0_]^−^ and [Y_0_-H]^−^ after deglycosylation. Take isoquercitrin as an example, in the negative ion mode, m/z 299.94183 and m/z 301.01648 fragment ions appeared simultaneously. The flavonoid aglycones will continue to disintegrate after deglycosylation, producing fragment ions such as [Y_0_-HCO]^−^, [Y_0_-HCO-CO]^−^, for example, the product ion at m/z 271.06042 [Y_0_-H-HCO]^−^ in the mass spectrum of isoquercitrin (Shahat et al., 2005; Gonzales et al., 2014; de Souza et al., 2016; Qin et al., 2017).

Leonurusoides A-D were found in Leonurus japonicus Houtt., they were isomers of each other. Their structural differences were the stereoscopic structures of the glucose group and galactosyl group, and the substitution positions of the syringyl group. In the negative ion chromatogram, the molecular ion peak at m/z 773.19 [M-H]^−^ was appeared at 16.40, 16.42, 16.46 and 16.50 min, in the MS^2^ spectra, the four compounds showed at m/z 593.15 [M-H-C_9_H_8_O_4_]^−^, 575.13 [M-H-C_9_H_8_O_4_-H_2_O]^−^, 285.04 [M-H-C_9_H_8_O_4_-C_6_H_10_O_4_-C_6_H_10_O_5_]^−^, 285.00 [Y_0_]^−^, 284.03 [Y_0_-H]^−^, indicating that they were flavonol glycosides containing syringyl group, two molecules of Glycosyl connected with the 3-position of the flavonol, the two Glycosy groups were linked at 1→6 positions. According to the polarity of the four compounds reported in the literature, the retention time of the four compounds on the RP-HPLC column was estimated to be Leonurusoide C < Leonurusoide D < Leonurusoide A < Leonurusoide B, so the peaks appeared at 16.40, 16.42, 16.46 and 16.50 min were Leonurusoide C, Leonurusoide D, Leonurusoide A, Leonurusoide B, respectively. At the same time, references confirmed the reliability of the inference (de Souza et al., 2008; Li et al., 2012).

##### Identification of Monoterpene Glycosides

By analyzing the fragmentation pathways of monoterpene glycosides in Paeonia lactiflora Pall, it could be summarized that if 2-bond and 4-bond ruptured simultaneously, the m/z 165 characteristic fragment ions of pinane would be produced. When the 3-bond broken, it will lose glucose, at the same time, it was easy to crack the 5-bond, resulting in the loss of a small neutral molecule. At last, owing to pinane was linked to different structures, benzoic acid or other different structural groups may be produced when the 1-bond was broken.

The peak of excimer ion in the negative ESI-MS mode at 8.01 min was m/z 525.16026 [M + COOH]^−^, which was the addition ion of paeoniflorin and formic acid. It can be seen that the product ions at m/z 478.99701, m/z 449.10016, m/z 341.10828, m/z 327.04996, m/z 283.03851, m/z 164.77895 in the second mass spectrum. Firstly, the loss of hydrogen ion produced the fragment ion at m/z 478.99701 [M-H]^−^, then the elimination of formaldehyde on the 6′-position of glucose group acquired the ion at m/z 449.10016 [M-H-H_2_CO]^−^; subsequently, the parent nucleus removed benzoic acid, which generated fragment ion at m/z 327.04996 [M-H-H_2_CO-C_7_H_6_O_2_]^−^, the ion fragment at m/z 283.03851 may be formed by the six-membered ring conversion of glucose group and benzoate group, the ion at m/z 164.77895 was a very critical characteristic fragment ion for identifying the structure of pinane. Compared with the standard, it was identified as Paeoniflorin with the same pyrolysis characteristics (Wang et al., 2013; Chen et al., 2020).

##### Identification of Phenolic Acids

Monomers of phenolic acids had simple structures and generally contained only the phenyl group. The excimer ion peak of [M-H]^−^ usually can be seen in the first full scan. For example, owing to the existence of -OH and -CO_2_, the [M-H]^−^ ion at m/z 197.04539 of Danshensu generated m/z 178.89343 [M-H-H_2_O]^−^ and m/z 134.98747 [M-H-H_2_O-CO_2_]^−^, the parent ion at m/z 137.02452 of Protocatechualdehyde generated the fragment at m/z 108.85629 [M-H-CO]^−^. For the polymers of phenolic acids, [M-H-198]^−^, [M-H-198-180]^−^, [M-H-198-198]^−^ ions were usually appeared, corresponding to the disconnection of Danshensu (198 Da) and caffeic acid (180 Da). Some of the hydrophilic phenolic compounds in ESI-MS-MS spectra had [2M-H]^−^ ion, for example, Lithospermic acid, Salvianolic acid B. It was easy to lose Danshensu (198 Da) and caffeic acid (180 Da), which were their common cleavage characteristics (Hu et al., 2005; Liang et al., 2017).

The product ions at m/z 537.06763 [M-H-C_9_H_8_O_4_]^−^ and m/z 519.10223 [M-H-C_9_H_10_O_5_]^−^ were produced by the excimer ion at m/z 717.14640 [M-H]^−^ respectively disconnected a caffeic acid (180 Da) and Danshensu (198Da). The fragment ions at m/z 699.48822 [M-H-H_2_O]^−^, m/z 673.22211 [M-H-CO_2_]^−^ correspond to the losses of H_2_O, CO_2_, respectively. The compound 117 was identified as Salvianolic acid B by comparing with the standard and literature data (Ma et al., 2021).

##### Identification of Diterpenes

The other major compounds in Salvia miltiorrhiza Bunge were lipophilic terpenoids, which had a good response in the positive ion mode. The excimer ion peak of [M + H]^+^, [M + Na]^+^ and the dimer garner peak of [2M + Na]^+^ usually can be seen in the first full scan. Cryptotanshinone and Tanshinone IIA both had unaromated A-ring, so it was easy to form enol at C_11_ position after getting a proton in the first-order mass spectrum, and then lost an H_2_O. Due to enolic rearrangement of a ketonic group, the product ion [M + H-18]^+^ was often seen in the MS^2^ spectrum of Tanshinones; sometimes, ions at [M + H-28]^+^, [M + H-18-15]^+^ or [M + H-28-15]^+^ were observed, suggesting the existence of -CO and -CH_3_ (Hu et al., 2005; Liang et al., 2017).

In the positive ion mode of ESI-MS, the protonated molecular ion [M + H]^+^ of Tanshinone IIA was at m/z 295.13312, the fragment ions of m/z 277.02783 and m/z 266.05096 indicated two fragment directions, corresponding to the losses of H_2_O and CO molecules, the precursor ion [M + H-H_2_O]^+^ at m/z 277.02783 fragmented into ions at m/z 249.04050 [M + H-18-28]^+^, m/z 221.17557 [M + H-18-28-28]^+^ (Ma et al., 2021).

##### Identification of Iridoid Glycosides

The deprotonated ion [M-H]^−^ of compound 34 was observed at m/z 373.11386 [M-H]^−^, due to the losses of one molecule of sugar (162 Da), one molecule of CO_2_ (44 Da), and one molecule of H_2_O (18 Da) successively, the parent ions formed the product ions at m/z 210.95450 [M-H-Glc]^−^, m/z 166.89352 [M-H-Glc-CO_2_]^−^, 148.93442 [M-H-Glc-CO_2_-H_2_O]^−^. The ions [M-H-Glc-CO_2_]^−^ at m/z 166.89352 and [M-H-Glc-2CO_2_]^−^ at m/z 122.87265 were produced by the predecessor ion [M-H-Glc]^−^ at m/z 210.95450 removed two molecules of CO_2_ successively. According to the characteristic series of fragment ions, it was identified as Geniposidic acid (Jiang et al., 2016).

Most of the saponins in Astragalus mongholicus Bunge belonged to cycloatenane type tetracyclic triterpenoid saponins with a furan ring at the C-17 position. In this study, these saponins produced typical adducts [M + Na]^+^ and [M + COOH]^−^ with high intensity in positive and negative ion mode, respectively. The mass spectra of cyclic aten saponins were highly similar, due to they contained the same aglycone skeleton and the same cleavage pathway. The MS^2^ spectrum information in negative mode can provide the position and type of sugar linkage on saponins (Neri-Numa et al., 2020).

Compound 145 exhibited a quasi-molecular ion [M-H + HCOOH]^−^ at m/z 829.45801 in the negative MS spectrum, the production ions at m/z 783.42084 [M-H]^−^, m/z 621.23438 [M-H-Glc]^−^, m/z 489.44446 [M-H-Glc-Xyl]^−^ were contributed to the losses of HCOOH (46 Da), Glc (162 Da), and Xyl (132 Da). By losing two water molecules, the parent ions at m/z 621.23438 and m/z 489.44446 generated the product ions at m/z 586.03680 [M-H-Glc-2H_2_O]^−^, m/z 453.50983 [M-H-Glc-Xyl-2H_2_O]^−^. Comparing with the standard and literature data, compound 145 was identified as Astragaloside IV (Chu et al., 2010; Liu et al., 2013).

The parent ion [M + Na]^+^ of Malonylastragaloside I appeared at m/z 977.47394 [M + Na]^+^ in the MS spectrum. The successive losses of CO_2_, Ac, H_2_O, Glc from quasi-molecular ion contributed to yield the fragment ions at m/z 933.42950 [M + Na-CO_2_]^+^, m/z 891.57025 [M + Na-CO_2_-Ac]^+^, m/z 873.37946 [M + Na-CO_2_-Ac-H_2_O]^+^, m/z 693.41779 [M + Na-CO_2_-Ac-H_2_O-Glu]^+^, the precursor ion at m/z 933.42950 [M + Na-CO_2_]^+^ fragmented into ions at m/z 753.49249 [M + Na-CO_2_-Glu]^+^. The product ions at m/z 657.42883 [M + Na-Xyl moiety]^+^, m/z 477.37054 [aglycone + Na]^+^, m/z 343.26492 [Na + Xyl moiety]^+^ were seen in the MS^2^ spectrum (Chu et al., 2010).

### Network Pharmacology Analysis

#### Components Absorbed Into Rat Blood and Nephrotic Syndrome-Related Targets

After excluding amino acids, a total of 16 components absorbed into the blood of rats were screened out, and their gene targets were obtained under the screening criteria OB ≥ 30% in the TCMSP database. In addition, 1714 nephrotic syndrome-related targets were acquired in three disease target databases. After analyzing the intersection genes of components and NS, 79 treatment targets and 16 compounds were obtained. The 16 components are exhibited in Table 1. The 79 targets are shown in Table 2.

**TABLE 1:** Names of compounds and drugs in the DDIG network.

MOI ID	Molecule name	OB score	Abbreviation	Drug name
MOL000173	Wogonin	30.68	AMB	Astragalus mongholicus Bunge
MOL000006	luteolin	36.16	AMK	Atractylodes macrocephala Koidz
MOL007134	Danshensu	36.91	ASD	Angelica sinensis (Oliv.)Diels
MOL000043	Atractylenolide Ⅰ	37.37	CPN	Codonopsis pilosula (Franch.) Nannf
MOL001452	Protocatechualdehyde	38.35	LJH	Leonurus japonicus Houtt
MOL002209	Senkyunolide G	39.52	LWF	Ligusticum wallichii Franch
MOL000360	Ferulic acid	39.56	PCW	Poria cocos(Schw.)Wolf
MOL000422	kaempferol	41.88	PLP	Paeonia lactiflora Pall
MOL000771	4-Hydroxycinnamic acid	43.29	PSL	Plantago asiatica L
MOL000417	Calycosin	47.75	SMB	Salvia miltiorrhiza Bunge
MOL007154	Tanshinone IIA	49.89		
MOL002095	Diethyl phthalate	52.19		
MOL007125	Neocryptotanshinone	52.49		
MOL001924	Paeoniflorin	53.87		
MOL010586	Formononetin	69.67		
MOL002178	4,7-Dihydroxy-3-butylphthalide	106.09		


**TABLE 2:** 79 targets identified for the intersected genes of Nephrotic syndrome and YQHXD.

Uniprot ID	Gene symbol	Target name
P22303	ACHE	Acetylcholinesterase
P60709	ACTB	Actin, cytoplasmic 1
P07550	ADRB2	Beta-2 adrenergic receptor
O95433	AHSA1	Activator of 90 kDa heat shock protein ATPase homolog 1
P31749	AKT1*	RAC-alpha serine/threonine-protein kinase
Q07812	BAX	Apoptosis regulator BAX
Q07817	BCL2L1*	Bcl-2-like protein 1
P0DP23	CALM1	Calmodulin-1
P42574	CASP3*	Caspase-3
P55211	CASP9	Caspase-9
P13500	CCL2*	C-C motif chemokine 2
P20248	CCNA2	Cyclin-A2
P24385	CCND1*	G1/S-specific cyclin-D1
P29965	CD40LG	CD40 ligand
P24941	CDK2	Cyclin-dependent kinase 2
P11802	CDK4	Cyclin-dependent kinase 4
P38936	CDKN1A	Cyclin-dependent kinase inhibitor 1
P20309	CHRM3	Muscarinic acetylcholine receptor M3
P02452	COL1A1	Collagen alpha-1(I) chain
P02461	COL3A1	Collagen alpha-1(III) chain
P10145	CXCL8*	Interleukin-8
P08684	CYP3A4	Cytochrome P450 3A4
P21728	DRD1	D(1A) dopamine receptor
P42892	ECE1	Endothelin-converting enzyme 1
P05305	EDN1*	Endothelin-1
P25101	EDNRA	Endothelin-1 receptor
P00533	EGFR*	Epidermal growth factor receptor
P56537	EIF6	Eukaryotic translation initiation factor 6
P03372	ESR1*	Estrogen receptor, ER
P00734	F2	Prothrombin
P02751	FN1*	Fibronectin, FN
P01100	FOS*	Proto-oncogene c-Fos
P09211	GSTP1	Glutathione S-transferase P
P07900	HSP90AA1*	Heat shock protein HSP 90-alpha
P05362	ICAM1*	Intercellular adhesion molecule 1
P01857	IGHG1	Immunoglobulin heavy constant gamma 1
P22301	IL10*	Interleukin-10
P01584	IL1B*	Interleukin-1 beta
P60568	IL2*	Interleukin-2
P05112	IL4*	Interleukin-4
P05231	IL6*	Interleukin-6
P01308	INS*	Insulin
P05106	ITGB3	Integrin beta-3
P05412	JUN*	Transcription factor AP-1
P35968	KDR	Vascular endothelial growth factor receptor 2
P09960	LTA4H	Leukotriene A-4 hydrolase
P61626	LYZ	Lysozyme C
P28482	MAPK1*	Mitogen-activated protein kinase 1
Q16539	MAPK14*	Mitogen-activated protein kinase 14
P45983	MAPK8*	Mitogen-activated protein kinase 8
Q07820	MCL1	Induced myeloid leukemia cell differentiation protein
Q00987	MDM2	E3 ubiquitin-protein ligase Mdm2
P08581	MET	Hepatocyte growth factor receptor
P03956	MMP1	Interstitial collagenase
P08253	MMP2*	Matrix metalloproteinase-2
P14780	MMP9*	Matrix metalloproteinase-9
P01106	MYC*	Myc proto-oncogene protein
P21359	NF1	Neurofibromin
P25963	NFKBIA*	NF-kappa-B inhibitor alpha
P35228	NOS2	Nitric oxide synthase, inducible
P29474	NOS3*	Nitric oxide synthase
P01160	NPPA	Natriuretic peptides A
P12004	PCNA	Proliferating cell nuclear antigen
P48736	PIK3CG	PI3-kinase subunit gamma
P00749	PLAU	Urokinase-type plasminogen activator
P37231	PPARG*	Peroxisome proliferator-activated receptor gamma
Q05655	PRKCD	Protein kinase C delta type
P23219	PTGS1	Prostaglandin G/H synthase 1
P35354	PTGS2*	Prostaglandin G/H synthase 2
Q04206	RELA*	Transcription factor p65
P19793	RXRA	Retinoic acid receptor RXR-alpha
P00441	SOD1	Superoxide dismutase
P42224	STAT1*	Signal transducer and activator of transcription 1-alpha/beta
P01137	TGFB1*	Transforming growth factor beta-1 proprotein
P01033	TIMP1	Metalloproteinase inhibitor 1
P01375	TNF*	Tumor necrosis factor
P04637	TP53*	Cellular tumor antigen p53
P15692	VEGFA*	Vascular endothelial growth factor A

The genes marked * represent the core genes.

**FIGURE 3:**
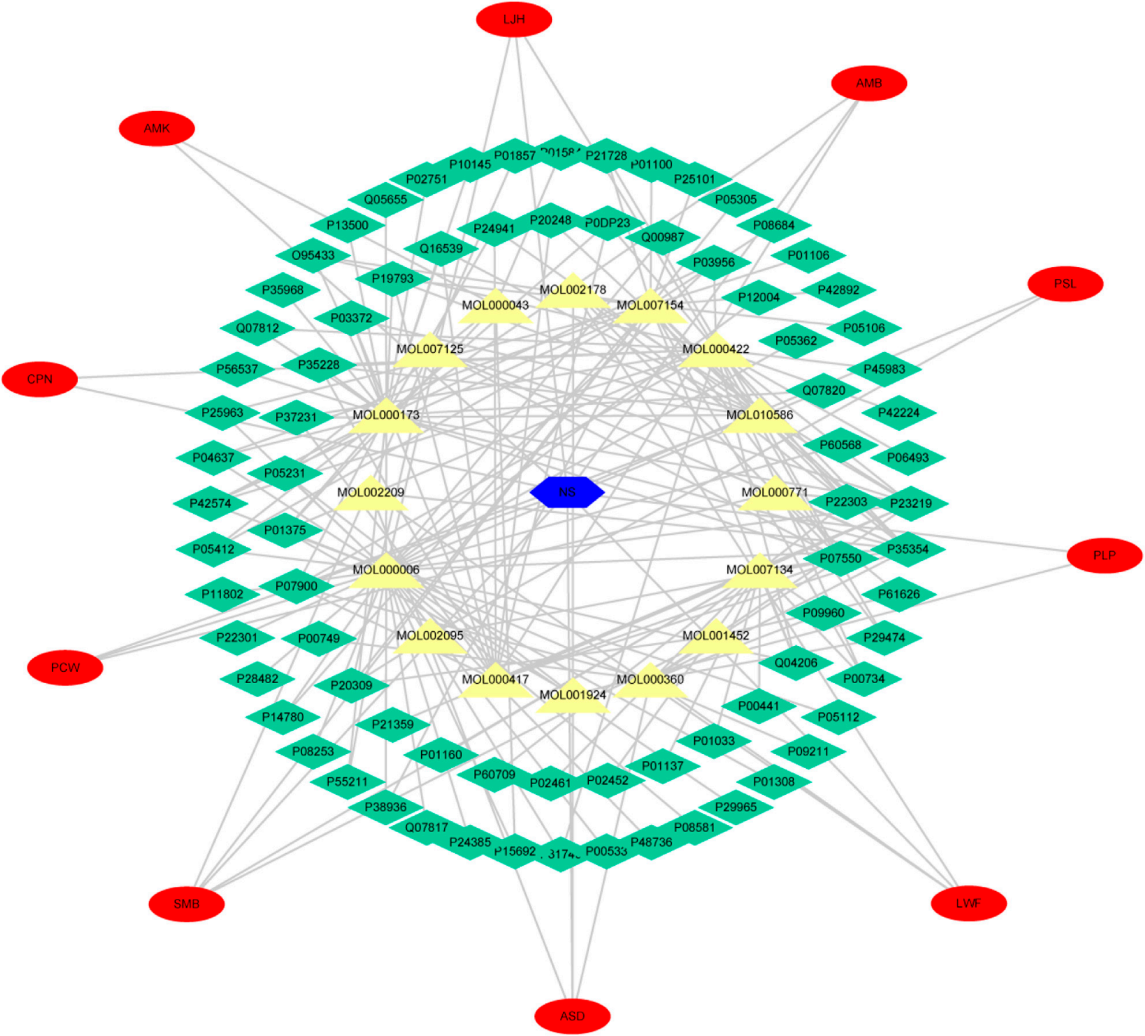
Disease-Drug-Ingredient-Gene (DDIG) network. (The blue hexagon means nephrotic syndrome; the yellow triangles mean the chemical components detected in rats’ plasma; the green diamonds mean the intersectional targets; the red ovals mean the 10 herbal medicines contained in YQHXD).

Cytoscape was used to analyze the topological structure and visualize the diagram of the Disease-Drug-Ingredient-Gene (DDIG) network (Figure 3). The degree values of this network were calculated by the NetworkAnalyzer tool. The higher the values, the more intimate the relationship between components and targets. This network contained 106 nodes (1 disease, 10 herbs, 16 candidate costituents, 79 targets) and 200 edges. The network centralization was 0.303 and the average number of neighbors was 3.774. The top 10 constituents and top 15 targets were screened using the degree and betweenness. They are shown in Tables 3, 4.

**TABLE 3:** The top 10 constituents screened out by the degree and betweenness.

Compounds	Degree	Compounds	Betweenness
luteolin	35	luteolin	0.3874176
Wogonin	28	Wogonin	0.23404949
Tanshinone IIA	23	Tanshinone IIA	0.20642444
kaempferol	21	Danshensu	0.16836507
Danshensu	16	kaempferol	0.15037821
Calycosin	13	Ferulic acid	0.04626873
Formononetin	12	Calycosin	0.03841274
Ferulic acid	9	Formononetin	0.03524434
Neocryptotanshinone	6	Neocryptotanshinone	0.02868901
4-Hydroxycinnamic acid	5	AtractylenolideⅠ	0.02636506

**TABLE 4:** The top 15 genes screened out by the degree and betweenness.

Targets	Degree	Targets	Betweenness
COX2	12	COX2	0.15981343
COX1	10	COX1	0.10795817
ADRB2	9	ADRB2	0.06680122
TNF	5	TNF	0.04372511
HSP90AA1	5	NFKB3	0.03810704
NFKB3	4	IL6	0.02649962
IL6	4	CASP3	0.02483077
CASP3	4	CDKN1A	0.01550499
CHRM3	4	JUN	0.01550499
RXRA	4	TP53	0.01550499
NOS2	4	CHRM3	0.0119666
CDKN1A	3	F2	0.01113072
JUN	3	ACHE	0.01113072
TP53	3	MMP1	0.01093697
F2	3	HSP90AA1	0.01030176

**TABLE 5:** The purity of RNA.

Name	Concentration	OD_260_/OD_280_	OD_260_/OD_230_
Control	3792.5	2.02	2.15
Model	3236.0	2.05	2.16
Calycosin 40 μM	3472.0	2.03	2.29
Calycosin 20 μM	3587.0	2.03	2.23
Calycosin 10 μM	2748.0	2.00	2.12
Formononetin 20 μM	2541.0	1.98	2.24
Formononetin 10 μM	2082.5	1.96	2.21
Formononetin 5 μM	2493.0	2.01	2.23
Wogonin 40 μM	2233.5	2.04	2.13
Wogonin 20 μM	2434.0	2.00	2.12
Wogonin 10 μM	2567.0	2.03	2.25
Luteolin 20 μM	2628.5	2.01	2.28
Luteolin 10 μM	2712.0	2.03	2.21
Luteolin 5 μM	2894.5	1.98	2.19
YQHXD 200 μg/ml	3067.0	1.96	2.22
YQHXD 100 μg/ml	3292.5	2.00	2.14
YQHXD 50 μg/ml	3454.0	1.97	2.15

**TABLE 6:** Primer sequences.

Gene name	Forward primer sequences (5′-3′)	Reverse primer sequences (5′-3′)
IL-1	CTGGTACATCAGCACCTCAC	AGAAACAGTCCAGCCCATAC
β-actin	CGTTGACATCCGTAAAGACC	TAGGAGCCAGAGCAGTAATC
Bcl-2	TTCAGGGATGGGGTGAACTG	CACAGGGCGATGTTGT
Podocin	GACGCTGTCTGCTACTACCG	AGTGAGGGATCGATGTGCCA
Bax	CCCGAGAGGTCTTCTTCC	GCCTTGAGCACCAGTTTG
Nephrin	CAGCGATGATGCGGAGTACG	CAGCTACCCAGGTAACTGTGC
TNF-α	ACCCTCACACTCAGATCATCTTC	TGGTGGTTTGCTACGACGT
IL-6	GAGGATACCACTCCCAACAGACC	AAGTGCATCATCGTTGTTCATACA
TGF-β	TTCCGCTGCTACTGCAAGTCA	GGGTAGCGATCGAGTGTCCA

#### Analyses of a PPI Network

**FIGURE 4:**
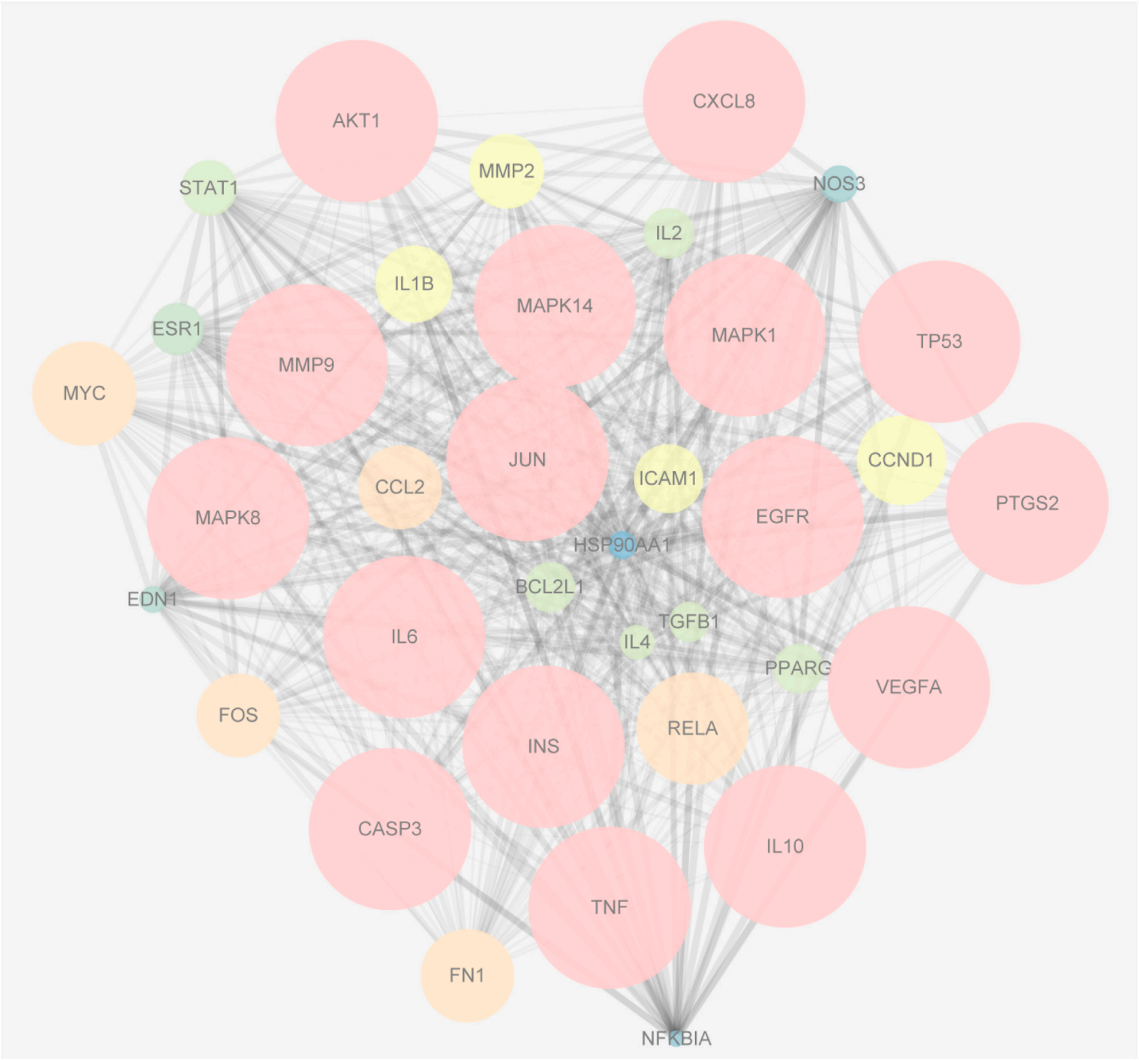
The PPI network of the 36 intersectional core targets. The bigger the node sizes, and the denser the color of the node, the more important the genes.

The 79 intersection genes were imported to the String database. The targets with a confidence value greater than 0.7 were screened out and transferred to Cytoscape for network construction. Using the Network Analyzer tool, the average degree of the nodes was calculated, and 36 core nodes larger than the average degree were screened out to draw the PPI network target of YQHXD-NS. In this PPI network, the bigger the node sizes, and the denser the color of the node, the more important the genes (Figure 4).

#### GO Enrichment and KEGG Pathway Analysis

**FIGURE 5:**
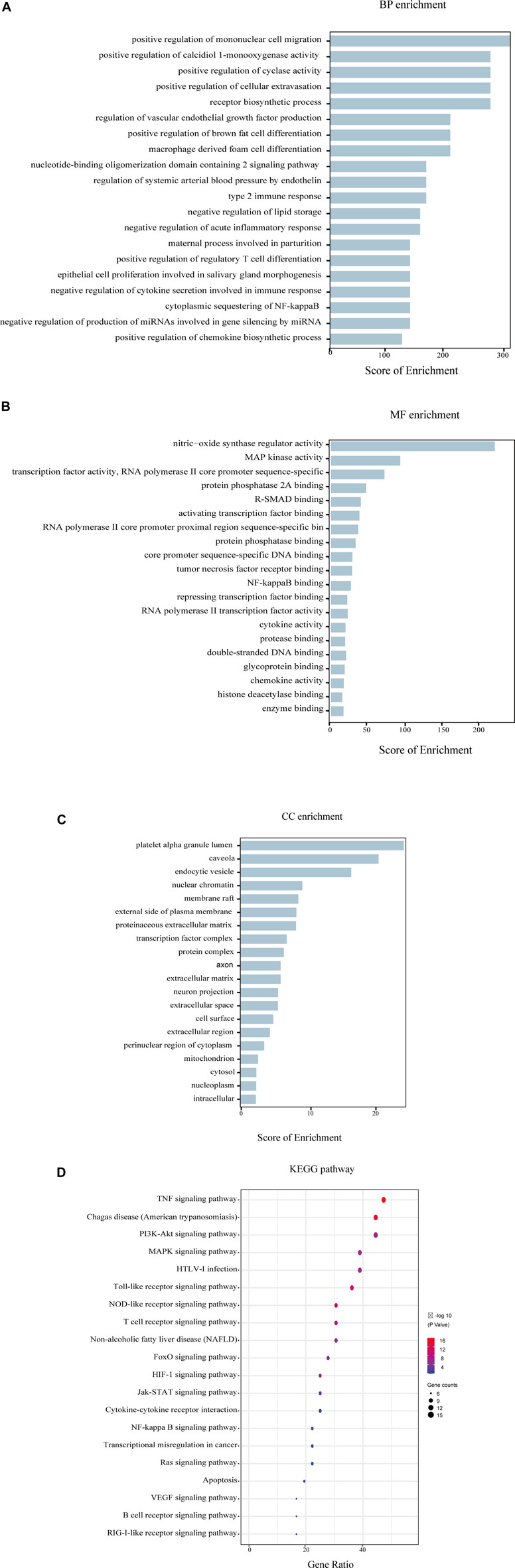
GO and KEGG enrichment analysis of the 36 intersectional core targets. **(A)** Biological process enrichment; **(B)** Molecular function enrichment; **(C)** Cellular component enrichment; **(D)** KEGG pathway enrichment.

To probe into the mechanisms of YQHXD on nephropathy, GO enrichment and KEGG pathway analysis were performed. The top 20 terms of cellular component (CC), biological process (BP), molecular function (MF) categories, and 20 main KEGG signaling pathways are shown in Figure 5. The results demonstrated the enrichment of the obtained targets in the external side of plasma membrane, negative regulation of lipid storage, cytoplasmic sequestering of NF-κB, inflammatory processes and apoptotic, which were consistent with the pathologic mechanisms of nephrotic syndrome.

Additionally, 36 core candidate targets were enriched in 20 KEGG pathways, and the enriched 20 pathways were divided into several categories: 1) signaling transduction of inflammation and oxidation, such as Cytokine-cytokine receptor interaction signaling pathway, NF-KB pathway; 2) signaling transduction of immunological reaction: T cell receptor signaling pathway, B cell receptor signaling pathway; 3) Pathways involved in cell function regulation: PI3K-Akt signaling pathway; 4) others.

### Cellular Experiment Results

#### MTT for Cell Viability

**FIGURE 6:**
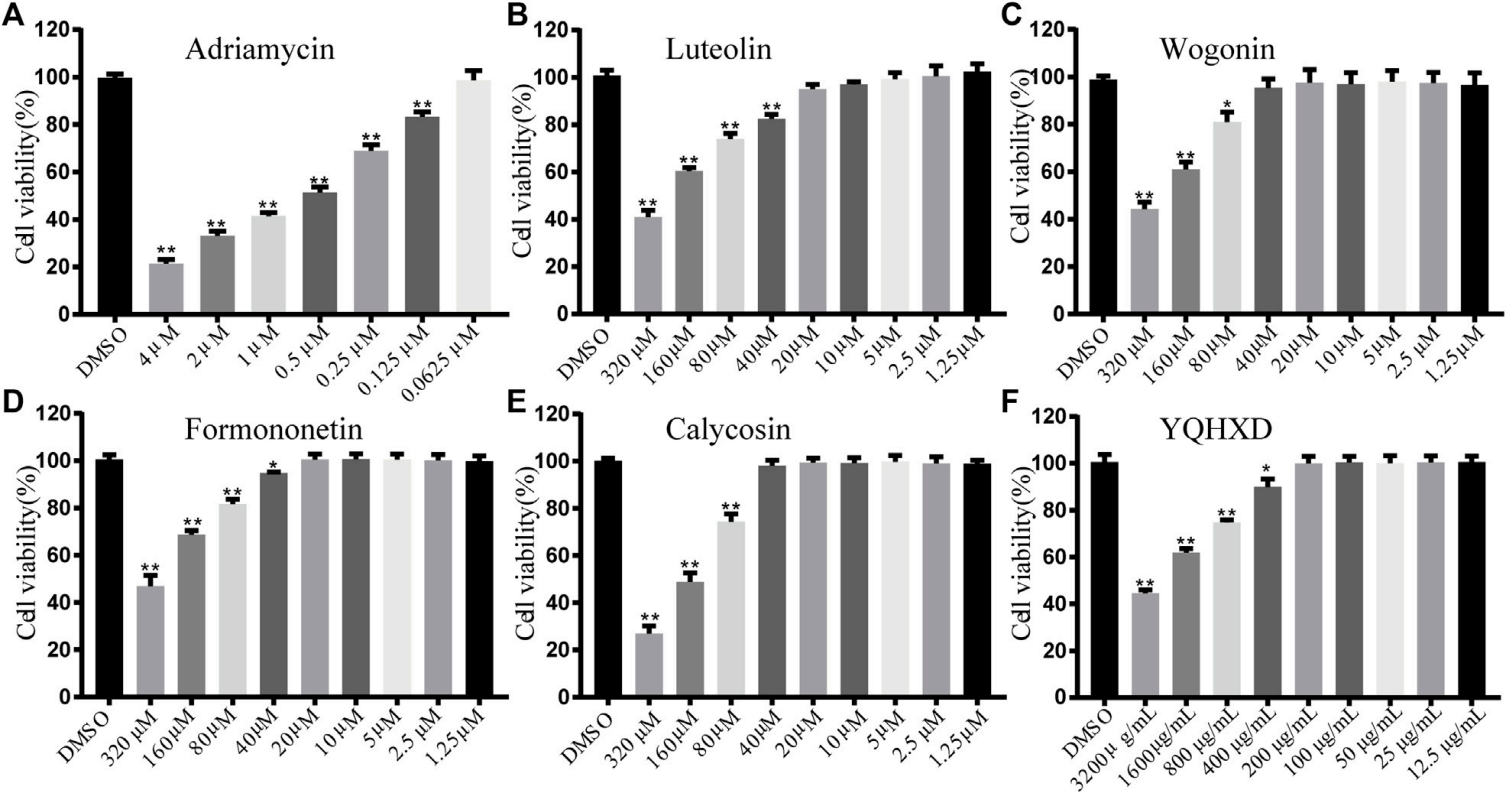
Determination of MPC-5 cell viability by MTT assay. **(A)** MPC-5 cells were treated by a series of concentrations of Adriamycin (from 6.25 × 10^−2^ μM to 4 μM); **(B)** MPC-5 cells were treated by a series of concentrations of Luteolin (from 1.25 to 320 μM); **(C)** MPC-5 cells were treated by a series of concentrations of Wogonin (from 1.25 to 320 μM); **(D)** MPC-5 cells were treated by a series of concentrations of Formononetin (from 1.25 to 320 μM); **(E)** MPC-5 cells were treated by a series of concentrations of Calycosin (from 1.25 to 320 μM); **(F)** MPC-5 cells were treated by a series of concentrations of YQHXD (from 12.5 μg/ml to 3,200 μg/ml). After being incubated with adriamycin or compounds for 24 h, cell viability was detected by MTT method at 490 nm wavelength. ^**^ *p* < 0.01, ^*^ *p* < 0.05 vs. DMSO group.

To determine the optimal concentration of each therapeutic agent administered, MTT assays were performed to determine the activity of DOX 4–0.0625 μM in a range of concentrations, Luteolin, Wogonin, Formononetin, and Calycosin at 320–1.25 μM in a range of concentrations, and YQHXD extracts at 3,200–6.25 μg/ml in a range of concentrations. As shown in Figure 6, the cell viability gradually decreased with the increase of DOX concentration, with the cell survival rate of 50% with 0.5 μM DOX; therefore, DOX at a concentration of 0.5 μM was used as a modeling agent. The cell viability of Luteolin and Formononetin delivered at concentrations less than 20 μM were in excess of 98%, so 20, 10, and 5 μM were considered as high, medium, and low dose administration groups. The cell viability of Wogonin and Calycosin delivered at concentrations less than 40 μM were in excess of 98%, so 40, 20, and 10 μM were considered as high, medium, and low dose administration groups. The cell viability of YQHXD delivered at concentrations less than 200 μg/ml were in excess of 98%, so 200, 100, and 50 μg/ml were considered as high, medium, and low dose administration groups.

#### Assay of SOD, CAT, MDA, GSH-Px

**FIGURE 7:**
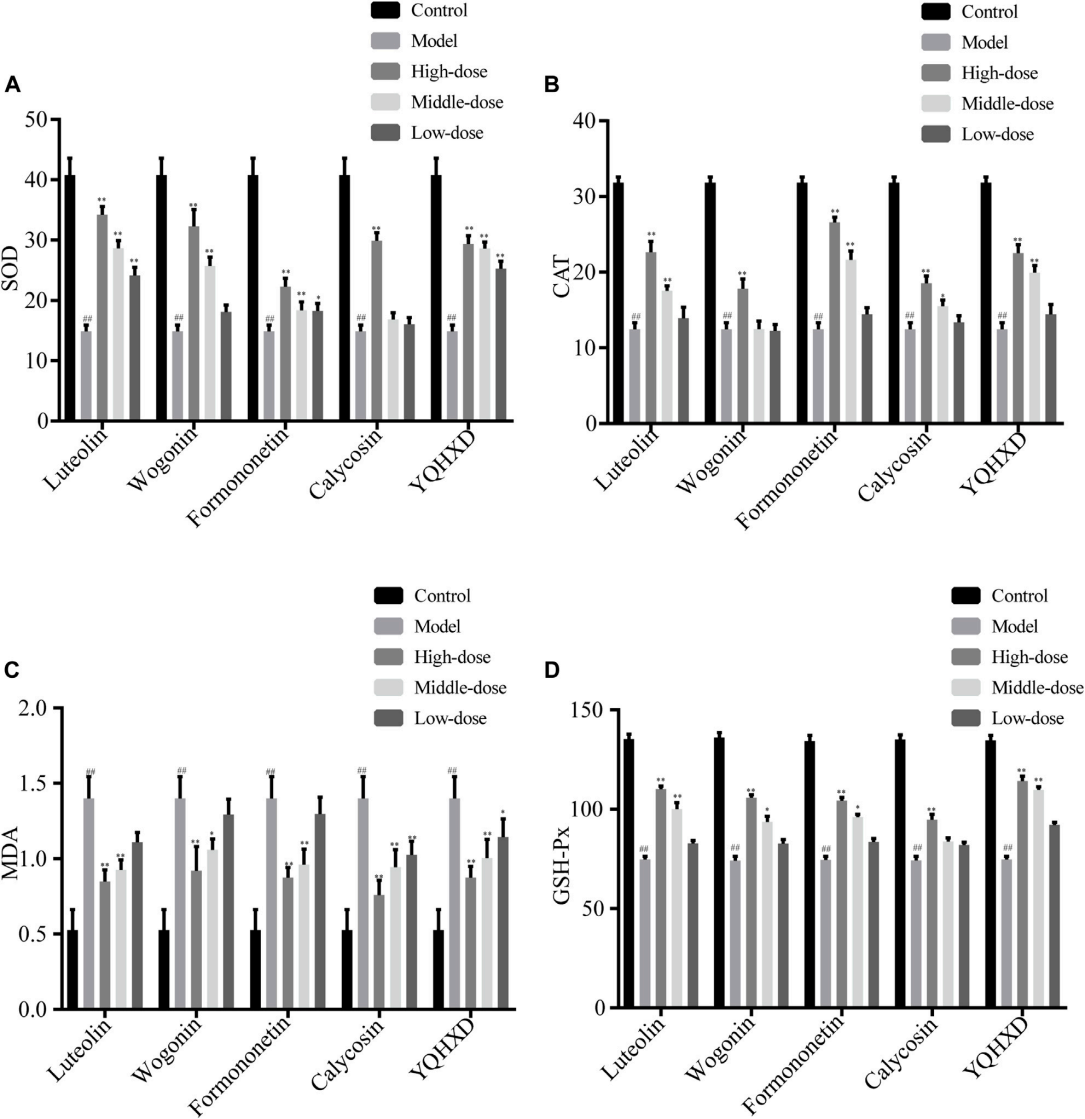
Effects of different Luteolin, Wogonin, Formononetin, Calycosin, and YQHXD on MPC5 cell oxidative stress induced by adriamycinMPC5 cells were placed in the culture dishes for 24 h, compounds of different concentrations and YQHXD were added respectively. At 48 h, adriamycin was added to each group. At 72 h, cell lysates from different drug treatment groups were collected and the activities of superoxide dismutase (SOD) **(A)**, catalase (CAT) **(B)**, malondialdehyde (MDA) **(C)**, glutathione peroxidase (GSH-Px) **(D)** were measured according to manufacturer’s protocols. ^**^ *p* < 0.01, ^*^ *p* < 0.05 vs. adriamycin group, ^##^ *p* < 0.01 vs. control group.

The regulation of oxidative stress levels in MPC5 cells by the five therapeutic agents is shown in Figure 7. The most significant elevation in SOD levels was observed in 20 μM, 10 μM, 5 μM Luteolin, 40 μM, 20 μM Wogonin, 20 μM, 10 μM Formononetin, 40 μM Calycosin and 200, 100 and 50 μg/ml YQHXD extracts groups, **p < 0.01. The SOD levels in 5 μM Formononetin increased significantly compared to the model group, *p < 0.05. 20 μM, 10 μM Luteolin and Formononetin, 40 μM Wogonin, 40, 20, 10 μM Calycosin, 200, 100 μg/ml YQHXD extracts significantly lowered the cellular MDA levels, **p < 0.01, and the MDA levels of cells were markedly decreased in 20 μM Wogonin, 50 μg/ml YQHXD groups, *p < 0.05. Compared with the model group, the CAT levels of cells were remarkably enhanced in 20 μM, 10 μM Luteolin and Formononetin, 40 μM Wogonin and Calycosin, 200 μg/ml, 100 μg/ml YQHXD extracts groups, **p < 0.01, and 20 μM Calycosin also significantly boosted the CAT levels of cells, *p < 0.05. The GSH-Px levels of cells were significantly elevated in the 20 μM, 10 μM Luteolin, 40 μM Wogonin and Calycosin, 20 μM Formononetin, 200 μg/ml, 100 μg/ml YQHXD extracts, **p < 0.01, and 20 μM Wogonin, 10 μM Formononetin also increased the GSH-Px level of cells, *p < 0.05.

#### Detection Results of Caspase 3 and Caspase 9

**FIGURE 8:**
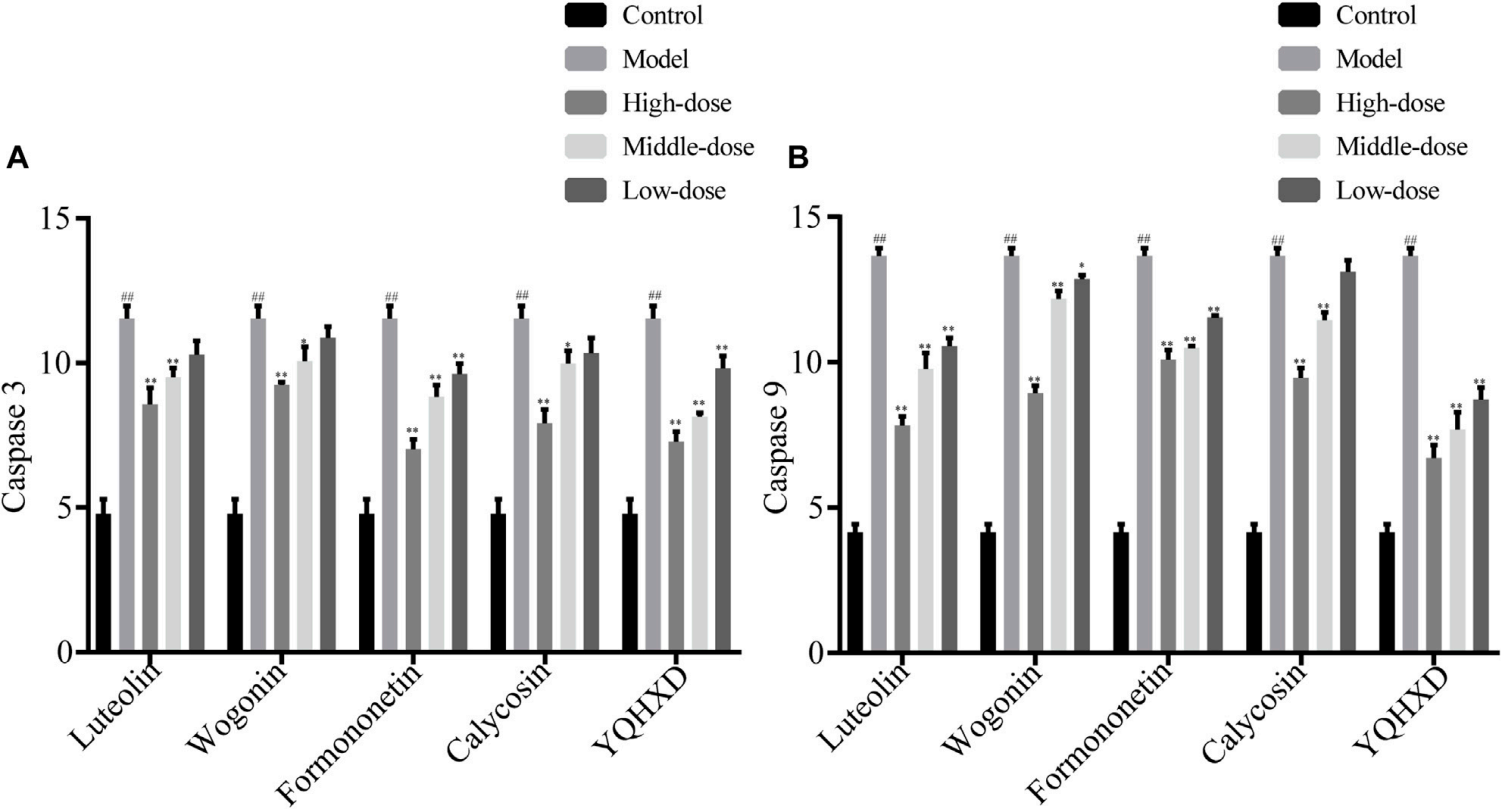
Effects of Luteolin, Wogonin, Formononetin, Calycosin, and YQHXD on MPC5 cell apoptosis induced by adriamycin. MPC5 cells were placed in the culture dishes for 24 h, compounds of different concentrations and YQHXD were added respectively. At 48 h, adriamycin was added to each group. At 72 h, cell lysates from different drug treatment groups were collected and the activities of Caspase 3 **(A)** and Caspase 9 **(B)** were measured according to manufacturer’s protocols. ^**^ *p* < 0.01, ^*^ *p* < 0.05 vs. adriamycin group, ^##^ *p* < 0.01 vs. control group.

The protective effects of the five drugs on cells at the apoptotic level are shown in Figure 8. Compared with the model group, 20 μM, 10 μM Luteolin, 40 μM Wogonin and Calycosin, 20, 10, 5 μM Formononetin and 200, 100 and 50 μg/ml YQHXD extracts markedly inhibited the expression level of cellular Caspase 3, **p < 0.01. 20 μM Wogonin also reduced the expression level of cellular Caspase 3, *p < 0.05. At the expression level of caspase 9 in cells, 20, 10, 5 μM Luteolin and Formononetin, 40 μM, 20 μM Wogonin and Calycosin, 200, 100 and 50 μg/ml YQHXD extracts were significantly lower than those of the model group, **p < 0.01, and 10 μM Wogonin was lower than that of the model group, *p < 0.05.

#### RT-qPCR Results

**FIGURE 9:**
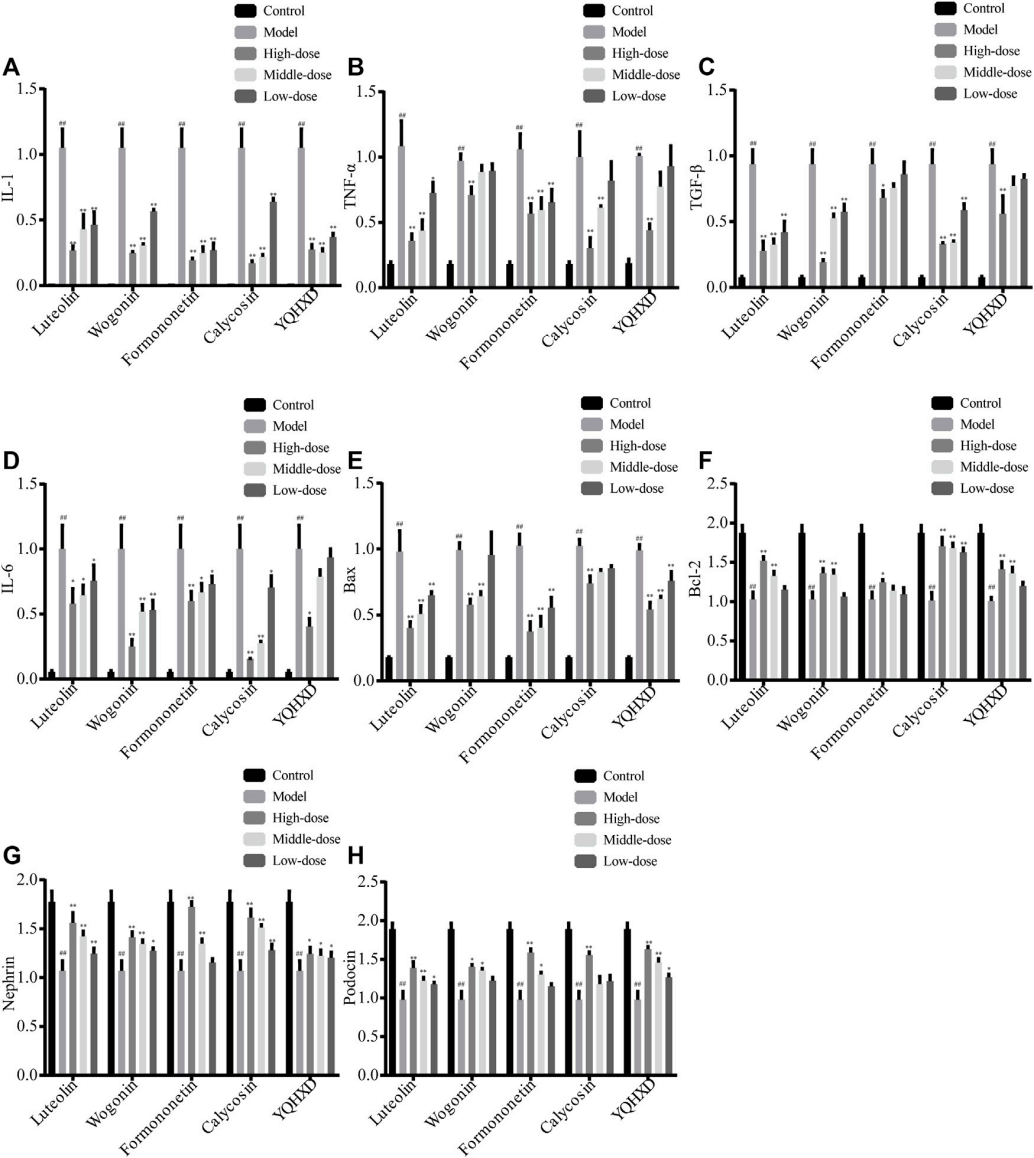
Effects of Luteolin, Wogonin, Formononetin, Calycosin, and YQHXD on mRNA of cytokines and transcription factors in MPC5 with adriamycin-induced nephropathy. **(A)** mRNA expression of IL-1; **(B)** mRNA expression of TNF-α; **(C)** mRNA expression of TGF-β; **(D)** mRNA expression of IL-6; **(E)** mRNA expression of Bax; **(F)** mRNA expression of Bcl-2; **(G)** mRNA expression of Nephrin; **(H)** mRNA expression of Podocin. ^**^ *p* < 0.01, ^*^ *p* < 0.05 vs. adriamycin group, ^##^ *p* < 0.01 vs. control group.

To investigate the mechanism of protective effects of YQHXD, Luteolin, Formononetin, Wogonin, and Calycosin on adriamycin-induced cell injury, a Real-Time PCR was used to determine the relative expression levels of interleukin 6 (IL-6), interleukin 1 (IL-1), tumor necrosis factor-α (TNF-α), transforming growth factor-β (TGF-β), B-cell lymphoma-2 (Bcl-2), Bcl 2-associated X protein (Bax), Podocin, and Nephrin protein mRNAs in the cells, as shown in Figure 9. The high, medium and low dose groups of all five drugs significantly reduced IL-1 expression levels compared to the model group, **p < 0.01. The 20, 10, and 5 μM Formononetin, 40 μM Wogonin and 200 μg/ml YQHXD extracts, 20 μM, 10 μM Luteolin and 40 μM, 20 μM Calycosin significantly drove down the expression level of TNF-α, **p < 0.01, and the 5 μM Luteolin also downregulated the mRNA expression level of TNF-α, *p < 0.05. The mRNA expression levels of cellular TGF-β were markedly inhibited by 20, 10, and 5 μM Luteolin, 40, 20, and 10 μM Wogonin and Calycosin, 200 μg/ml YQHXD extracts, **p < 0.01, and 20 μM Formononetin also brought down the mRNA expression level of cellular TGF-β, *p < 0.05. 40, 20, 10 μM Wogonin, 40 μM, 20 μM Calycosin, 20 μM Formononetin remarkably brought down the cellular IL-6 protein mRNA expression level, **p < 0.01; 20 μM, 10 μM, 5 μM Luteolin, 10 μM, 5 μM Formononetin and 10 μM Calycosin also reduced the mRNA expression level of cellular IL-6 protein, *p < 0.05. 20 μM, 10 μM Luteolin, 40 μM, 20 μM Wogonin, 40 μM, 20 μM, 10 μM Calycosin and 200 μg/ml, 100 μg/ml YQHXD extracts remarkably enhanced the mRNA expression level of Bcl-2 protein in cells, **p < 0.01; 20 μM Formononetin also elevated the mRNA expression level of Bcl-2 protein in cells, *p < 0.05. 20 μM, 10 μM Luteolin, 20 μM Formononetin, 40 μM Calycosin, 200 μg/ml, 100 μg/ml YQHXD extracts markedly upregulated Podocin gene expression levels, **p < 0.01, and 40 μM, 20 μM Wogonin, 5 μM Luteolin, 10 μM Formononetin, 50 μg/ml YQHXD extracts also elevated Podocin gene expression level, *p < 0.05. 20, 10, 5 μM Luteolin, 40 μM, 20 μM, 10 μM Calycosin, 40 μM, 20 μM Wogonin, 20 μM, 10 μM Formononetin, contributed to the increase of Nephrin gene expression, **p < 0.01, 10 μM Wogonin, 200, 100, and 50 μg/ml YQHXD extracts, *p < 0.05.

## Discussion

Kidney disease is becoming a global disease seriously threatening human health, Nephrotic syndrome (NS) is a type of chronic nephropathy, which may be caused by plenty of factors, such as environmental factors, immune factors. Due to its complex pathogenesis and lingering disease, the treatment is limited, few validated methods are available at present. Thus, We urgently need to find effective therapeutic drugs and methods.

YQHXD has been used to treat nephrotic syndrome for decades, and the therapeutic effect of YQHXD on NS is well recognized. However, few studies reported its effective substances and mechanism. UPLC-ESI-LTQ-Orbitrap XL/MS can quickly and efficiently identify hundreds of components in TCM herbs. In this study, we analyzed 233 chemical constituents in 35 min by UPLC-MS, to provide the foundation for further research on the active components.

In traditional pharmacology for TCM study, medicine or extract were added to cells *in vitro* or animal organs *in vivo*. However, many constituents of herbal medicines may take effect only after biological transformations such as digestion and absorption of the gastrointestinal tract. The physical and chemical properties of traditional herbal medicines or extracts may result in physiological changes in osmotic pressure and pH value *in vitro* reaction system, which, therefore, predisposes to false positive or negative results. Serum pharmacochemistry firstly proposed by Tashiro (Naito et al., 2012) overcome these shortcomings. The experimental conditions of serum pharmacochemistry are similar to the effective drug environment *in vivo*. Accordingly, the proposed serum pharmacochemistry provides a new method for studying the effective substances of YQHXD. Rats were used in our pharmacokinetic study, and 50 absorbed prototypes of YQHXD in rats’ plasma were identified, which may contribute to clarifying the potential mechanism of YQHXD through network pharmacology.

To clarify the potential mechanism of YQHXD in treating NS, the serum of rat gavage with the extract of YQHXD was collected and UPLC/MS identified 50 ingredients. Through the network pharmacological analysis of the components of YQHXD that enter the blood, the TCMSP database was used to screen the targets of the components that enter the blood, and the GeneCards database, DisGeNET database, OMIM database were used to identify the combined NS targets, followed by the screening of the intersection targets of NS and YQHXD. The Cytoscape database was employed to identify potential active ingredients including Luteolin, Formononetin, Wogonin, Calycosin, as well as the core targets, among which the targets with high freedom include tumor necrosis factor (TNF), serine/threonine matrix metalloproteinase 9 (MMP9), RAC serine/threonine-protein kinase 1, (AKT1), interleukin-6 (IL-6), caspase 3 (CASP3), fibronectin 1 (FN1), and cyclooxygenase 2 (COX2).

The family of AKT contains three close members, namely, RAC serine/threonine-protein kinase 1/2/3 (AKT1/2/3). Activated AKT regulates the activity of various downstream molecules to achieve the regulation of cellular autophagy, apoptosis, glycolipid metabolism, oxidative stress levels, inflammation, and other physiological functions of the organism (Wang et al., 2019). Additionally, AKT promotes activation of inhibitor of nuclear factor kappa-B kinase (IKK) to accelerate phosphorylation and degradation of nuclear factor κB, thereby facilitating the transfer of nuclear factor κB from the cytoplasm to the nucleus and subsequent binding to specific sequences in deoxyribonucleic acid, which consequently result in gene transcription. The above signaling cascades eventually lead to extracellular matrix (ECM) proliferation (Sun et al., 2014). Furthermore, phosphorylated NF-κB boosts the transcription of pro-inflammatory cytokines such as TNF-α and IL-6 (Chow et al., 2005). IL-6 is secreted by renal thylakoid cells, which mediate endothelial permeability and contributes to glomerular basement membrane thickening and thylakoid expansion (Dalla Vestra et al., 2005).

T-cell subpopulation imbalance and dysfunction are involved in the development of NS (Fiser et al., 1991; Neuhaus et al., 1995), as evidenced by the presence of a large infiltration of inflammatory cells and the overexpression of the inflammatory cytokines TNF and interleukin 1 (IL-1) in the peripheral blood of children with primary NS and mice with adriamycin nephropathy (Lv et al., 2017). TNF plays a vital role in biological processes such as apoptosis, inflammation and, immunity, where low concentrations of TNF-α exert effects on regulating inflammatory response, repairing tissue damage, and anti-infection, while upregulation of TNF-α eliminates immune homeostasis and triggers pathological damage. Various stimulating conditions cause the release of TNF-α from renal cells, which induces a large accumulation of ROS, lipid peroxides, and lipid metabolites in the glomerular basement membrane, giving rise to negative changes in microvascular permeability and damage to the glomerular structure and thus producing proteinuria. TNF-α antagonists can effectively reduce the expression of TNF-α mRNA in the renal cortex, thereby alleviating the inflammatory state of the kidney and preventing proteinuria (Perez-Gracia et al., 2009; Wang et al., 2012).

In renal disease, multiple stimuli may lead to the synthesis of reactive oxygen species, among which nicotinamide adenine dinucleotide phosphate (NADPH)-induced oxidative stress upregulates the expression of apoptosis regulator Bax proteins and downregulates apoptosis regulator Bcl-2 content. The overexpression of Bax triggers the overexpression of factors such as caspase 3, thereby promoting apoptosis.

By setting the confidence greater than 0.7, 36 targets with closer interaction were selected from 79 intersection targets imported into the David database. David database was used to analyze the functional enrichment of 36 core target genes. The results of GO functional enrichment analysis demonstrated that the targets of YQHXD are mainly concentrated in biological processes such as tumor necrosis factor binding, extracellular matrix, and other cellular components, negative regulation of lipid deposition, and cytoplasmic segregation of NF-κB. The main signaling pathways analyzed by KEGG pathway enrichment include TNF, PI3K-AKT, and NF-κB signaling pathways which play an important role in the development of NS, as confirmed by several previous studies.

The PI3K-AKT signaling pathway is a classical signal transduction pathway that regulates renal pathological changes strongly associated with podocyte injury and thylakoid stromal proliferation. Recent studies have illustrated that the PI3K-AKT signaling pathway is closely related to the therapeutic effects of the “Beneficial Qi and Blood Stasis Method,” “Beneficial Qi and Blood Activation Method,” and “Removal of Blood Stasis and Ligament Method” in the treatment of kidney disease in TCM. AKT promotes activation of IKK to accelerate phosphorylation and degradation of nuclear factor κB, thereby facilitating the transfer of nuclear factor κB from the cytoplasm to the nucleus and subsequent binding to specific sequences in deoxyribonucleic acid, which consequently results in gene transcription. NF-κB, a transcription factor, is closely involved in the regulation of renal disease progression, as it promotes the transcription of pro-inflammatory cytokines, such as TNF-α, IL-6, and ICAM-1 (Chow et al., 2005). Studies have confirmed that inhibition of NF-κB activation reduces proteinuria and attenuates renal injury.

Podocytes are a key component of the selective permeability barrier of the glomerular basement membrane, where podocytes connect and cover the surface of the glomerular basement membrane through slit septa to regulate permeability. They wrap around capillaries to form filtration slits that maintain the structural integrity of the glomerular filtration membrane, which underscores the importance of the loss of podocytes in renal disease progression. ADR-induced podocyte apoptosis leads to diminished glomerular filtration barrier function, which may contribute to the development of proteinuria. During adriamycin-induced nephropathy, the loss of podocytes may result in podocyte detachment. PI3K/AKT and its downstream signaling pathways can regulate various physiopathological processes such as oxidative stress, inflammatory response, and apoptosis in podocytes. Che et al. (2015) found that ADR disrupts mitochondrial redox homeostasis in MPC5 cells and generates excess ROS and that the overexpression of ROS and TGF-β1 activates the PI3K/AKT signaling pathway, acting on multiple targets such as Bcl-2 and Caspase 3 in podocytes and mediating apoptosis in podocytes (Lee and Song 2010). Moreover, Yu et al. (2010) revealed that excess TGF-β1 could also directly disassemble the actin backbone of podocytes and promote podocyte apoptosis; ROS and Akt can activate the NF-kB signaling pathway and promote the transcription of pro-inflammatory factors such as TNF-α, IL-6, and ICAM-1 to mediate the inflammatory response.

Formononetin, Wogonin, Calycosin, and Luteolin et al. were selected as compounds with potential therapeutic activity by network pharmacology experiment. After treatment with Compound extracts, Formononetin, Wogonin, Calycosin, and Luteolin, the treatment groups obtained enhanced expression levels of SOD, CAT, and GSH-Px and decreased expression levels of MDA, caspase 3, and caspase 9 in MPC5 cells when compared with the model group. The results of the RT-PCR assay showed that each treatment group showed lowered TNF, TGF-β, IL-1, IL-6, Bax mRNA expression levels and elevated Podocin, Nephrin, and Bcl-2 mRNA levels in MPC5 cells to different degrees, indicating that the five therapeutic agents alleviate oxidative stress, inflammatory response, and apoptosis in MPC5 cells through the NF-κB signaling pathway, and reduce the alteration of podocyte structure. The results of this experiment validate the results of network pharmacology experiments.

## Conclusion

In this study, 233 compounds in YQHXD and 50 components absorbed in the blood of rats were identified qualitatively for the first time by the UPLC-MS technique, which laid the foundation for the quality control of YQHXD in the future. Results of MTT assay, oxidative stress assay, apoptosis, and RT-qPCR assay demonstrated that the four active compounds exert positive effects on the oxidative stress, inflammatory response, and apoptosis of MPC5 cells through NF-κB signaling pathway. This study provides a reference for the development of new drugs for the treatment of NS through analysis of TCM and compound extracts.

This study investigated the protective effects of Luteolin, Formononetin, Calycosin, and Wogonin on adriamycin-induced MPC5 injury in mouse podocytes. In the future, studies of other chemical components in YQHXD on other cytopathological models of the kidney, such as the model of adriamycin-induced thylakoid cell injury and the model of adriamycin-induced renal tubular epithelial cell injury, or the pharmacological efficacy and mechanism of action of YQHXD on the rat model of the nephrotic syndrome from the perspective of *in vivo* experiments, will be conducted to explore in-depth the multiple pathological mechanisms of kidney injury protection by YQHXD and its chemical components.

## Data Availability

The original contributions presented in the study are included in the article/Supplementary Material, further inquiries can be directed to the corresponding author H-GZ (435792387@qq.com).

## Glossary

AKT: RAC serine/threonine-protein kinase
AMB: Astragalus mongholicus Bunge
AMK: Atractylodes macrocephala Koidz
ASD: Angelica sinensis (Oliv.) Diels
Bax: Apoptosis regulator Bax
Bcl-2: Apoptosis regulator Bcl-2
BP: Biological process
CAL: Calycosin
CAT: Catalase
CC: Cellular component
COX2: Cyclooxygenase 2
CPN: Codonopsis pilosula (Franch.) Nannf
DDIG: Disease-drug-ingredient-gene
DOX: Doxorubicin
ECM: Extracellular matrix
ELISA: Enzyme-linked immuno sorbent assay
ESRD: End-stage renal disease
FN1: Fibronectin 1
FOR: Formononetin
GBM: Glomerular basement membrane
GO: Gene biological process
GSH-Px: Glutathione peroxidase
ICAM-1: Intercellular adhesion molecule 1
IKK: Inhibitor of nuclear factor kappa-B kinase
IL-1: Interleukins 1
IL-6: Interleukin 6
KEGG: Encyclopedia of Genes and Genomes
LJH: Leonurus japonicus Houtt
LUT: Luteolin
LSD: Ligusticum striatum DC. (syn. Ligusticum wallichii Franch)
MDA: Malondialdehyde
MF: Molecular function
MMP9: Matrix metalloproteinase 9
MPC5: Mouse podocyte clone 5
NADPH: Nicotinamide adenine dinucleotide phosphate
NF-KB: Nuclear factor kappa-B
NS: Nephrotic Syndrome
P65: Nuclear factor kappa-B subunit p65
WEG: Wolfiporia extensa (Peck) Ginns [syn. Poria cocos (Schw.)]
PI3K: Phosphatidylinositol 3 kinase
PLP: Paeonia lactiflora Pall
PPI: Protein-protein interaction network
PSL: Plantago asiatica L.
ROS: Reactive oxygen species
SMB: Salvia miltiorrhiza Bunge
SOD: Superoxide dismutase
TCM: Traditional Chinese Medicine
TGF-β: Transforming growth factor β
TNF-α: Tumor necrosis factor α
UPLC-MS: Ultra-High Performance Liquid Chromatograph-Mass
WOG: Wogonin
YQHXD: Yiqi Huoxue Decoction
