# Supplementary material for: Integrated UPLC-MS and Network Pharmacology Approach to Explore the Active Components and the Potential Mechanism of Yiqi Huoxue Decoction for Treating Nephrotic Syndrome
Source: Front Pharmacol. 2022 Feb 24;12:775745. doi: 10.3389/fphar.2021.775745 (PMC8919777; doi:10.3389/fphar.2021.775745)
Supplement: Supplementary file 1 [file Table1.docx]

Table S1: Identification of compounds in YQHXD by UPLC-MS^n^.

| **NO.** | **RT**  **min** | **Experical Formula** | **Proposal Ions** | **Experimental  Mass m/z** | **Theoretical Mass m/z** | **Mass Error (ppm)** | **MS^2^/MS^3^** | **Identification** | **Crude drug** |
| --- | --- | --- | --- | --- | --- | --- | --- | --- | --- |
| 1* | 0.83 | C_6_H_15_N_4_O_2_ | [M+H]^+^ | 175.11926 | 175.11895 | 0.308 | MS^2^: 157.95340, 129.78351, 115.75787  MS^3^: 139.87259, 115.89117, 111.81084, 69.81972 | Arginine^(1)^ | AMB, CPN |
| 2 | 0.93 | C_5_H_14_ON | [M+H]^+^ | 104.10710 | 104.10699 | 0.109 | MS^2^:103.87338, 59.84368  MS^3^[103.87338]: 59.84368 | Choline^(1)^ | AMB, CPN |
| 3 | 0.95 | C_6_H_11_O_7_ | [M-H] ^-^ | 195.05081 | 195.04992 | 0.881 | MS^2^: 176.76375, 158.82448, 151.11443, 128.86224  MS^3^[128.86224]: 84.81838, 56.69693 | Gluconic acid^(2)^ | LJH |
| 4 | 0.96 | C_12_H_22_O_11_Cl | [M-H]^-^ | 377.08527 | 377.08451 | 0.754 | MS^2^: 340.99722, 214.93752  MS^3^[340.99722]: 178.80373, 160.82779, 142.92180, 112.79413 | Cl-maititol^(2)^ | LJH |
| 5 | 1.34 | C_18_H_32_O_16_Na | [M+Na]^+^ | 527.15765 | 527.15825 | -0.606 | MS^2^: 365.11737, 335.41119, 305.00702, 275.12787, 244.86111,  MS^3^[365.14355]: 305.00702, 275.12787, 202.99748, 184.88310 | Raffinose^(1)^ | AMB, CPN |
| 6 | 1.46 | C_24_H_42_O_21_Na | [M+Na]^+^ | 689.21075 | 689.21107 | -0.329 | MS^2^: 527.16205, 509.13049, 467.28510, 437.19269, 407.08044  MS^3^[527.16205]: 365.08655, 347.12878 | Stachyose^(1)^ | AMB, CPN |
| 7 | 1.51 | C_30_H_52_O_26_Na | [M+Na]^+^ | 851.26471 | 851.2639 | 0.808 | MS^2^:689.21759, 671.25671, 629.01331, 527.18573 MS^3^[527.16205]: 509.17572, 365.12604, 347.04980 | Verbascose^(3)^ | SMB |
| 8 | 1.59 | C_6_H_7_O_7_ | [M-H]^-^ | 191.01952 | 191.01862 | 4.664 | MS^2^:172.89397, 128.94228, 110.74937  MS^3^[110.74937]: 82.88403, 66.74864  MS^3^[110.74937]: 82.88403, 66.74864 | Citric acid^(2)^ | LJH |
| 9 | 1.66 | C_5_H_8_NO_3_ | [M+H]^+^ | 130.04976 | 130.04986 | -0.110 | MS^2^:83.72221  MS^3^[83.72221]: 55.71096 | Pyroglutamic acid^(1)^ | AMB |
| 10 | 1.69 | C_36_H_62_O_31_Na | [M+Na]^+^ | 1013.31775 | 1013.31672 | 1.024 | MS^2^: 851.26428, 833.20203, 761.41119  MS^3^[83.72221]: 689.26501, 671.12933, 527.19458, 365.11066 | Inulin^(1)^ | CPN |
| 11 | 1.82 | C_15_H_21_O_9_ | [M-H]^-^ | 345.11789 | 345.11800 | -0.344 | MS^2^: 327.06122, 182.89284, 179.06851, 164.87560  MS^3^[164.87560]: 146.92934, 136.94272, 122.87170, 94.74318 | Galiridoside^(2)^ | LJH |
| 12 | 1.96 | C_4_H_5_N_2_O_2_ | [M+H]^+^ | 113.03458 | 113.03455 | 0.026 | MS^2^:112.82144, 95.85370, 94.84018, 70.66897, 68.90097 | Uracil^(1)^ | CPN, AMB |
| 13* | 2.02 | C_6_H1_4_NO_2_ | [M+H]^+^ | 132.10196 | 132.10190 | 0.055 | MS^2^:85.72563  MS^3^[85.72563]: 68.75626 | L-Isoleucine^(1)^ | CPN, AMB |
| 14 | 2.06 | C_5_H_6_N_5_ | [M+H]^+^ | 136.06169 | 136.06177 | -0.082 | MS^2^: 118.84700, 93.90338, 90.87380 | Adenine^(1)^ | CPN, AMB |
| 15 | 2.09 | C_9_H_12_NO_3_ | [M+H]^+^ | 182.08138 | 182.08116 | 0.210 | MS^2^: 164.8773, 135.90851, 119.00150 | Tyrosine^(1)^ | AMB |
| **NO.** | **RT**  **min** | **Experical Formula** | **Proposal Ions** | **Experimental  Mass m/z** | **Theoretical Mass m/z** | **Mass Error (ppm)** | **MS^2^/MS^3^** | **Identification** | **Crude drug** |
| 16* | 2.22 | C6H14NO2 | [M+H]+ | 132.10191 | 132.10190 | 0.005 | MS^2^: 85.90927  MS^3^[85.90927]: 68.81940, 57.93541 | L-leucine^(1)^ | CPN, AMB |
| 17 | 2.30 | C_12_H_21_O_11_ | [M-H]^-^ | 341.10828 | 341.10783 | 0.442 | MS^2^:178.94203, 160.91943, 142.85519  MS^3^[178.94203]: 160.90410, 142.83720, 130.89168, 112.88484, 88.85274 | Sucrose^(4)^ | AMB |
| 18 | 3.03 | C_10_H_17_N_3_O_6_ | [M+H]^+^ | 275.11038 | 275.11118 | -0.807 | MS^2^: 257.08472, 230.89331, 184.96429 | Glu-Gln^(1)^ | AMB |
| 19* | 3.31 | C_7_H_5_O_5_ | [M-H]^-^ | 169.01404 | 169.01314 | 0.890 | MS^2^: 124.81490  MS^3^[124.81490]: 106.86255, 96.86565, 80.84547, 68.65929 | Gallic acid^(5)^ | PLP |
| 20* | 3.35 | C_10_H_12_N_5_O_5_ | [M-H]^-^ | 282.08395 | 282.08329 | 0.655 | MS^2^: 238.64334, 149.84167, 132.88622  MS^3^[149.84167]: 132.81181, 106.75858, 81.79887 | Guanosine^(4)^ | AMB |
| 21* | 4.18 | C_9_H_12_NO_2_ | [M+H]^+^ | 166.08632 | 166.08625 | 0.065 | MS^2^: 130.81908, 119.87080  MS^3^[119.87080]: 102.79120, 92.74552 | Phenylalanine^(1)^ | AMB |
| 22 | 4.19 | C_8_H_10_N | [M+H]^+^ | 120.0807 | 120.08077 | -0.076 | MS^2^:119.79787, 102.86913, 76.76981  MS^3^[119.79787]: 93.64606, 92.85684 | 2-ethenylbenzenamine^(6)^ | AMK |
| 23 | 4.31 | C_13_H_15_O_10_ | [M-H]^-^ | 331.0665 | 331.06597 | 0.527 | MS^2^: 312.98999, 270.97589, 240.99274, 168.83777  MS^3^[168.83777]: 150.77710, 124.79620, 96.94051 | Galloylglucose^(7)^ | PLP |
| 24 | 4.36 | C_19_H_25_O_15_ | [M-H]^-^ | 493.11987 | 493.11987 | 1.074 | MS^2^: 330.99487, 313.08810, 283.04141, 240.88846, 168.90074 | Diglucosyl gallic acid | PSL |
| 25 | 4.40 | C_10_H_14_N_5_O_3_ | [M+H]^+^ | 252.10933 | 252.10911 | 0.214 | MS^2^: 233.97900, 205.99562  MS^3^[205.99562]: 188.94131, 180.83562, 162.92493, 156.01187, 137.80386 | 2'-Deoxyadenosine | AMB |
| 26 | 4.41 | C_7_H_5_O_4_ | [M-H]^-^ | 153.01926 | 153.01823 | 1.025 | MS^2^: 137.97835, 122.74815, 108.76680 | Protocatechuic acid^(3)^ | SMB |
| 27 | 4.45 | C_19_H_25_O_15_ | [M-H]^-^ | 493.12006 | 493.11879 | 1.264 | MS^2^: 330.98578, 313.05179, 271.02475, 168.95485  MS^3^[313.05179]: 222.94998, 178.99437, 168.81038, 150.78036, 124.79082 | β-D-glucopyranosyl-(6'->1'')-β-D-glucopyranosyl-3,4,5-trihydroxybenzoate | PSL |
| 28 | 4.58 | C_6_H_5_O_2_ | [M-H]^-^ | 109.03055 | 109.0284 | 1.554 | MS^2^: 108.84438, 80.81097, 65.89143 | Catechol^(8)^ | PSL |
| 29 | 4.66 | C11H19O8 | [M-H]- | 279.10831 | 279.10744 | 0.866 | MS^2^: 116.89970, 100.84070, 88.82893 | Methyl-4-(β-D-glucopyranosyloxy)  -butanoate^(2)^ | LJH |
| 30 | 4.68 | C14H22NO4 | [M+H]+ | 268.15442 | 268.15433 | 0.085 | MS^2^:160.94862, 87.89188  MS^3^[87.89188]: 69.85638, 57.75333 | Codonopsine^(1)^ | CPN |
| **NO.** | **RT**  **min** | **Experical Formula** | **Proposal Ions** | **Experimental  Mass m/z** | **Theoretical Mass m/z** | **Mass Error (ppm)** | **MS^2^/MS^3^** | **Identification** | **Crude drug** |
| 31* | 4.69 | C_9_H_9_O_5_ | [M-H]^-^ | 197.04539 | 197.04444 | 0.940 | MS^2^: 178.89343, 153.01146, 134.98747, 122.95027, 72.77243 | Danshensu^(3)^ | SMB |
| 32 | 4.69 | C_5_H_12_NO_2_ | [M+H]^+^ | 118.08632 | 118.08625 | 0.065 | MS^2^: 99.78278, 87.79321, 57.70499  MS^3^[99.78278]: 71.84724, 57.89290, | Betaine^(1)^ | AMB |
| 33 | 4.76 | C_18_H_23_O_12_ | [M-H]^-^ | 431.11932 | 431.1184 | 2.128 | MS^2^: 395.14304, 299.10004, 178.85144, 136.80876  MS^3^[136.80876]: 92.77856 | Asperulosidic acid^(4)^ | PSL, AMB |
| 34 | 4.83 | C_16_H_21_O_10_ | [M-H]^-^ | 373.11386 | 373.11292 | 0.937 | MS^2^: 210.95450, 166.89352, 148.93442, 122.87265  MS^3^[210.97523]: 193.13991, 166.98793, 148.97276, 122.88731 | Geniposidic acid^(9)^ | PSL |
| 35 | 4.96 | C_7_H_5_O_4_ | [M-H]^-^ | 153.0193 | 153.01823 | 1.065 | MS^2^: 152.93460, 137.97835, 122.74815, 108.76680 | Gentisic acid^(10)^ | LJH |
| 36 | 5.15 | C_25_H_37_O_16_ | [M-H]^-^ | 593.20776 | 593.20761 | 0.251 | MS^2^: 461.18817  MS^3^[461.18817]: 315.04419, 297.08289, 160.91916, 134.83649 | Leonuriside B^(2)^ | LJH |
| 37 | 5.18 | C_20_H_29_O_12_ | [M-H]^-^ | 461.16583 | 461.16535 | 1.035 | MS^2^: 315.01263, 160.88373  MS^3^[315.01263]: 152.86441, 134.88045 | Verbasoside^(2)^ | LJH |
| 38 | 5.21 | C_20_H_29_O_12_ | [M-H]^-^ | 461.16583 | 461.16535 | 1.035 | MS^2^: 315.05771, 160.97922, 152.86441, 134.84363 | Forsythoside E^(2)^ | LJH |
| 39 | 5.27 | C_11_H_13_N_2_O_2_ | [M+H]^+^ | 205.09721 | 205.09715 | 0.056 | MS^2^:187.87297  MS^3^[187.87297]: 145.88432, 132.18178 | Tryptophane^(1)^ | AMB, LWF |
| 40 | 5.40 | C_21_H_27_O_13_ | [M-H]^-^ | 487.14557 | 487.14461 | 1.956 | MS^2^: 451.17996, 178.85152 | Cistanoside F^(11)^ | LJH |
| 41* | 5.65 | C_10_H_9_O_4_ | [M-H]^-^ | 193.05035 | 193.04955 | 0.815 | MS^2^: 177.79839, 148.83275  MS^3^[148.83275]: 133.84254 | Ferulic acid^(1)^ | AMB, LWF |
| 42 | 5.65 | C_7_H_5_O_3_ | [M-H]^-^ | 137.02452 | 137.02332 | 1.199 | MS^2^: 136.86267, 108.85629, 92.74164 | Protocate chualdehyde^(3)^ | SMB |
| 43 | 5.74 | C_16_H_17_O_11_ | [M-H]^-^ | 385.07709 | 385.07653 | 0.552 | MS^2^: 367.00946, 208.85280, 190.90523 | 3-Trans-feruloyl glucaric acid^(2)^ | LJH |
| 44 | 5.77 | C_16_H_17_O_11_ | [M-H]^-^ | 385.07709 | 385.07653 | 0.552 | MS^2^: 367.11530, 208.92587, 190.81560  MS^3^[190.81560]: 172.70015, 146.92197, 128.84642, 84.72361 | 4-Trans-feruloyl glucaric acid^(2)^ | LJH |
| 45 | 5.88 | C_23_H_27_O_12_ | [M-H]^-^ | 495.15063 | 495.1497 | 1.873 | MS^2^: 465.17966, ,333.02591, 327.09024  MS^3^[465.17966]: 299.04221, 281.04617, 164.90170, 136.76549 | Oxypaeoniflora^(12)^ | PLP |
| 46* | 5.93 | C_9_H_7_O_3_ | [M+H]^+^ | 163.03915 | 163.03897 | 0.179 | MS^2^:162.91995, 144.82370, 134.83873  MS^3^[144.82370]: 116.74153, 88.78174 | 4-Hydroxycoumarin | AMK |
| 47 | 5.97 | C_20_H_27_O_3_ | [M+H]^+^ | 315.19598 | 315.19522 | 0.509 | MS^2^: 297.19635, 270.12323, 214.03906, 176.97142, 159.91626, 139.02878  MS^3^[176.97142]: 159.91626, 130.90193 | 6,7-Dedihydroroyleanone | SMB |
| **NO.** | **RT**  **min** | **Experical Formula** | **Proposal Ions** | **Experimental  Mass m/z** | **Theoretical Mass m/z** | **Mass Error (ppm)** | **MS2/MS3** | **Identification** | **Crude drug** |
| 48* | 6.13 | C_16_H_17_O_9_ | [M-H]^-^ | 353.08731 | 353.0867 | 0.601 | MS^2^: 190.96951, 178.96909, 134.90536  MS^3^[190.96951]: 172.90059, 126.93199 | Chlorogenic acid^(1)^ | AMB |
| 49 | 6.28 | C_24_H_25_O_16_ | [M-H]^-^ | 569.11438 | 569.11371 | 1.176 | MS^2^: 371.06659, 326.97485, 196.90370, 172.92039 | Disyringoyl-glucaric acid^(2)^ | LJH |
| 50 | 6.56 | C_8_H_7_O_4_ | [M-H]^-^ | 167.03487 | 167.03388 | 0.985 | MS^2^: 122.93953  MS^3^[122.93953]: 120.98064, 78.86518 | Vanillic acid^(1)^ | CPN, AMB |
| 51 | 6.56 | C_7_H_7_O_2_ | [M-H]^-^ | 123.04542 | 123.044 | 1.364 | MS^2^: 122.87732, 107.86415, 94.88289, 78.78002 | 2-Hydroxymethylphenol | AMK |
| 52 | 6.71 | C_9_H_7_O_4_ | [M-H]^-^ | 179.03473 | 179.03388 | 0.845 | MS^2^: 178.99605, 134.86198 | Caffeic acid^(3)^ | SMB |
| 53 | 6.83 | C_18_H_27_O_9_ | [M-H]^-^ | 387.16553 | 387.16495 | 1.475 | MS^2^: 369.12234, 341.15280, 206.97254, 162.98172  MS^3^[206.97254]: 188.97475, 162.91951, 144.88344, 108.89615 | Tuberonic acid glucoside^(2)^ | LJH |
| 54 | 6.92 | C_25_H_34_O_12_Na | [M+Na]^+^ | 549.19385 | 549.19424 | -0.398 | MS^2^: 387.09875, 352.16, 335.24, 307.27  MS^3^[387.09875]: 369.08572, 351.18655, 339.09326, 323.97546 | 2-[[1,3-Dihydroxy-1-(4-hydroxy-3-methoxyphenyl)-2-propanyl]oxy]-5-(3-hydroxypropyl)phenyl-β-D-glucopyranoside | AMK |
| 55 | 7.25 | C_29_H_38_O_16_Na | [M+Na]^+^ | 665.20538 | 665.2052 | 0.262 | MS^2^: 543.21393, 503.15228, 433.09595  MS^3^[503.15228]: 381.16235, 341.14087, 218.95612, 184.93724 | Isomucronulatol- 2 ,5 -di-O-glucoside^(1)^ | AMB |
| 56 | 7.43 | C_24_H_29_O_13_ | [M+COOH]^-^ | 525.16058 | 525.16026 | 0.313 | MS^2^: 479.02856, 449.05393, 356.94409, 327.12445, 283.07562, 164.86261 | Albiflorin^(13)^ | PLP |
| 57 | 7.67 | C_9_H_9_O_5_ | [M-H]^-^ | 197.04546 | 197.04444 | 1.010 | MS^2^: 181.87932, 152.87021  MS^3^[152.87021]: 137.84151, 120.81390, 106.89204, 93.84306, 80.96028 | Syringate^(14)^ | CPN |
| 58 | 7.81 | C_17_H_19_O_9_ | [M-H]^-^ | 367.103 | 367.10235 | 1.747 | MS^2^: 190.98914, 172.91974  MS^3^[190.98914]: 172.97151, 126.82137, 110.93315, 84.78492 | 3-Feruloylquinic acid^(2)^ | LJH |
| 59 | 8.01 | C_19_H_29_O_10_S | [M-H]^-^ | 449.14514 | 449.14759 | -2.454 | MS^2^: 327.04169, 309.09335, 164.95142  MS^3^[327.04169]: 309.11789, 164.84540, 122.83923 | (6R,9S)-3-oxo-a-ionol-b-D  -glucopyranoside Sulfate^(15)^ | CPN |
| 60 | 8.01 | C_24_H_29_O_13_ | [M+COOH]^-^ | 525.15936 | 525.16026 | -0.907 | MS^2^: 478.99701, 449.10016, 341.10828, 327.04996, 283.03851  MS^3^[449.05383]: 327.12445, 309.00684, 164.86261 | Paeoniflorin^(5)^ | PLP |
| 61 | 8.01 | C_22_H_25_O_10_ | [M-H]^-^ | 449.14514 | 449.14422 | 2.041 | MS^2^: 327.04169, 309.09335, 164.95142  MS^3^[327.04169]: 309.11789, 164.84540, 122.83923 | Melampyroside^(16)^ | PSL |
| 62 | 8.11 | C_9_H_9_O_4_ | [M+H]^+^ | 181.04939 | 181.0495 | -0.035 | MS^2^: 170.97614, 152.80428  MS^3^[152.80428]: 137.85674, 109.83069 | 4-(methoxycarbonyl) benzoic acid^(6)^ | PSL |
| **NO.** | **RT**  **min** | **Experical Formula** | **Proposal Ions** | **Experimental  Mass m/z** | **Theoretical Mass m/z** | **Mass Error (ppm)** | **MS2/MS3** | **Identification** | **Crude drug** |
| 63 | 8.14 | C_14_H_22_N_3_O_5_ | [M+H]^+^ | 312.15482 | 312.15539 | -0.577 | MS^2^: 295.11661, 180.92271, 113.81650  MS^3^[180.92271]: 152.92918, 137.90398 | Leonurine^(2)^ | LJH |
| 64 | 8.38 | C_24_H_29_O_13_ | [M+COOH]^-^ | 525.16034 | 525.16026 | 0.073 | MS^2^: 478.99701, 449.10016, 341.10828,  MS^3^[449.10016]: 327.04996, 309.05792, 164.77895 | Mudanpioside I^(5)^ | PLP |
| 65 | 8.89 | C_11_H_20_N_3_O_2_ | [M+H]^+^ | 226.15468 | 226.155 | -0.323 | MS^2^:207.97479, 83.75900 | Plantagoguanidinic acid^(17)^ | PSL |
| 66 | 9.04 | C_25_H_25_O_15_ | [M-H]^-^ | 565.11932 | 565.11879 | 0.927 | MS^2^: 388.95172, 371.06549, 366.97827, 196.88901, 172.88747  MS^3^[371.06549]: 326.97443, 265.01651, 196.94946, 172.97107 | 5-Feruloyl-6-syringyl-glucaric acid^(2)^ | LJH |
| 67 | 9.08 | C_25_H_25_O_15_ | [M-H]^-^ | 565.11932 | 565.11879 | 0.927 | MS^2^: 389.01825, 371.13336, 367.07855, 196.96416, 172.81006 | 2-Feruloyl-6-syringyl-glucaric acid^(2)^ | LJH |
| 68 | 9.21 | C_25_H_25_O_15_ | [M-H]^-^ | 565.11932 | 565.11879 | 0.927 | MS^2^: 389.00055, 371.04663, 366.94116, 196.91179, 172.87247 | 4-Feruloyl-6-syringyl-glucaric acid^(2)^ | LJH |
| 69 | 9.27 | C_18_H_33_O_11_ | [M-H]^-^ | 425.20255 | 425.20173 | 0.812 | MS^2^: 263.10965, 160.91109 | Hexyl-b-D-glucopyranosyl- (1/2)-b-D-glucopyranoside^(1)^ | CPN |
| 70 | 9.37 | C_25_H_25_O_15_ | [M-H]^-^ | 565.11932 | 565.11879 | 0.927 | MS^2^: 389.09229, 371.01898, 367.13660,  197.05856, 172.80008 | 3-Feruloyl-6-syringyl-glucaric acid^(2)^ | LJH |
| 71 | 9.53 | C_29_H_35_O_16_ | [M-H]^-^ | 639.19208 | 639.19196 | 0.194 | MS^2^: 477.22302  MS^3^[477.22302]: 315.16354, 178.88724, 160.89789, 134.88246 | Plantainoside D^(18)^ | PSL |
| 72* | 9.57 | C_22_H_23_O_10_ | [M+H]+ | 447.12842 | 447.12857 | -0.153 | MS^2^: 285.11108 MS^3^[285.11108]: 270.04926, 253.02771, 224.96863, 136.84692 | Calycosin-7-O-β-D-glucoside^(19)^ | AMB |
| 73* | 9.80 | C_27_H_29_O_16_ | [M-H]^-^ | 609.14532 | 609.14501 | 0.516 | MS^2^: 300.94659  MS^3^[300.94659]: 178.95331, 150.79285 | Rutin^(1)^ | LWF |
| 74* | 10.07 | C_21_H_19_O_12_ | [M-H]^-^ | 463.08817 | 463.0871 | 1.068 | MS^2^: 300.98126  MS^3^[300.98126]: 178.83394, 150.80750 | Hyperoside^(2)^ | LJH |
| 75 | 10.28 | C_34_H_43_O_19_ | [M-H]^-^ | 755.24011 | 755.2393 | 0.805 | MS^2^: 623.27838, 593.21771, 461.23724  MS^3^[593.21771]: 461.14435, 315.17023 | Lavandulifolioside^(2)^ | LJH |
| 76 | 10.36 | C_15_H_11_O_7_ | [M+H]^+^ | 303.04971 | 303.04992 | -0.219 | MS^2^: 285.06100, 256.97601, 228.91638  MS^3^[256.97601]: 229.05922, 201.03844 | Morin | PSL |
| 77* | 10.37 | C_21_H_19_O_12_ | [M-H]^-^ | 463.08838 | 463.0871 | 1.278 | MS^2^: 301.01648, 299.94183, 271.06042  MS^3^[301.01648]: 178.89627, 150.74750 | Isoquercitrin^(2)^ | LJH |
| 78 | 10.50 | C_30_H_31_O_15_ | [M-H]^-^ | 631.16913 | 631.16632 | 0.573 | MS^2^: 613.11310, 509.15125, 491.21387, 313.13113  MS^3^[313.13113]: 253.06006, 169.03035 | Galloylpaeoniflorin^(5)^ | PLP |
| **NO.** | **RT**  **min** | **Experical Formula** | **Proposal Ions** | **Experimental  Mass m/z** | **Theoretical Mass m/z** | **Mass Error (ppm)** | **MS2/MS3** | **Identification** | **Crude drug** |
| 79 | 10.50 | C_21_H_19_O_11_ | [M-H]^-^ | 447.0929 | 447.09218 | 0.712 | MS^2^: 327.05292, 283.95972, 254.95102 | Luteoloside^(20)^ | PSL |
| 80 | 10.72 | C_29_H_35_O_15_ | [M-H]^-^ | 623.19720 | 623.19704 | 0.153 | MS^2^: 579.41931, 461.21570  MS^3^[461.21570]: 315.22729, 297.11133, 160.95361, 134.88475 | Verbascoside^(21)^ | LJH |
| 81* | 10.93 | C_24_H_25_O_13_ | [M-H]^-^ | 521.13 | 521.12896 | 1.033 | MS^2^: 359.02155, 323.14716  MS^3^[359.02155]: 223.02548, 196.86319, 178.88339, 160.90002 | Salviaflaside^(22)^ | SMB |
| 82 | 11.04 | C_12_H_18_O_4_Na | [M+Na]^+^ | 249.10992 | 249.10973 | 0.636 | MS^2^: 231.03471, 221.04207, 192.77687 | Senkyunolide J^(23)^ | LWF |
| 83 | 11.07 | C_12_H_17_O_3_ | [M+H]^+^ | 209.11736 | 209.11722 | 0.139 | MS^2^: 208.98354, 190.98758, 162.92674, 152.82274  MS^3^[190.98758]: 162.92674, 134.84969 | Senkyunolide G^(24)^ | LWF |
| 84 | 11.11 | C_29_H_35_O_16_ | [M-H]^-^ | 639.19244 | 639.19196 | 0.757 | MS^2^: 477.22308 MS^3^[477.22308]: 315.03537, 296.97528, 178.92883, 160.98169, 134.90576 | Plantamajoside^(25)^ | PSL |
| 85 | 11.18 | C_26_H_38_O_13_Na | [M+Na]^+^ | 581.22009 | 581.22046 | -0.372 | MS^2^: 419.29553, 363.13177, 365.17181, 347.14612  MS^3^[363.13177]: 275.07355, 258.98889, 201.05130, 184.90662 | Lobetyolinin^(1)^ | CPN |
| 86 | 11.36 | C_30_H_31_O_15_ | [M-H]^-^ | 631.16632 | 631.16602 | 0.273 | MS^2^: 613.12073, 509.27612, 313.03009  MS^3^[313.03009]: 168.82948, 150.89832, 124.83952 | 4'-O-galloylalbiflorin^(26)^ | PLP |
| 87 | 11.43 | C_29_H_37_O_15_ | [M-H]^-^ | 625.21338 | 625.21269 | 1.093 | MS^2^: 463.17603  MS^3^[463.17603]: 317.09598, 299.16107, 160.77255, 134.82385 | Isomucronulatol-7,2’-di-O-glucoside^(4)^ | AMB |
| 88* | 11.54 | C_27_H_29_O_15_ | [M-H]^-^ | 593.15100 | 593.15009 | 0.904 | MS^2^: 285.02339, 257.00323  MS^3^[285.02339]: 166.95825, 256.88660, 240.97241, 150.90431 | Nicotiflorin^(2)^ | LJH |
| 89 | 11.61 | C_18_H_11_O_7_ | [M-H]^-^ | 339.05194 | 339.05051 | 0.581 | MS^2^: 320.97131, 295.12335, 184.84634  MS^3^[295.12335]: 279.94873, 276.97412, 249.07336, 184.91165 | Salvianolic acid G^(3)^ | SMB |
| 90 | 11.62 | C_27_H_21_O_12_ | [M-H]^-^ | 537.10352 | 537.10275 | 0.768 | MS^2^: 339.01581, 295.13248  MS^3^[339.01581]: 321.05212, 295.10727, 277.11200, 184.91032 | Salvianolic acid H/I/J/isomer^(3)^ | SMB |
| 91 | 11.7 | C_27_H_21_O_12_ | [M-H]^-^ | 537.10352 | 537.10275 | 0.768 | MS^2^: 493.12628, 338.97675, 294,97565, 184.96826  MS^3^[338.97675]: 321.09021, 295.09113, 280.09692, 184.96826 | Lithospermic acid^(3)^ | SMB |
| 92* | 11.75 | C_7_H_5_O_3_ | [M-H]^-^ | 137.02467 | 137.02332 | 1.349 | MS^2^: 136.85126, 108.88208, 92.76646 | Salicylic acid^(2)^ | LJH |
| 93 | 11.82 | C_16_H_17_O_9_ | [M-H]^-^ | 353.08701 | 353.0867 | 0.301 | MS^2^: 190.90866, 178.89023, 134.95793  MS^3^[190.90866]: 172.84814, 126.79234, 92.86646, 84.84809 | 5-Caffeoylquinic acid^(2)^ | LJH |
|  |  |  |  |  |  |  |  |  |  |
| **NO.** | **RT**  **min** | **Experical Formula** | **Proposal Ions** | **Experimental  Mass m/z** | **Theoretical Mass m/z** | **Mass Error (ppm)** | **MS2/MS3** | **Identification** | **Crude drug** |
| 94 | 11.82 | C_29_H_35_O_15_ | [M-H]^-^ | 623.19727 | 623.19704 | 0.223 | MS^2^: 477.24353, 461.21729  MS^3^[461.21729]: 315.03937, 297.06610, 134.98495 | Isoverbascoside^(21)^ | LJH |
| 95 | 11.95 | C_30_H_31_O_15_ | [M-H]^-^ | 631.16522 | 631.16632 | -0.527 | MS^2^: 613.15424, 509.23334, 463.21777, 312.99274 | 4-O-galloylalbiflorin^(26)^ | PLP |
| 96 | 11.99 | C_25_H_25_O_13_ | [M+H]^+^ | 533.12842 | 533.12896 | -0.547 | MS^2^: 284.97424 MS^3^[284.97424]: 269.98206, 252.93817, 224.95963 | Isomer of calycosin-7-O-Glc-6"-O-malonate^(4)^ | AMB |
| 97 | 12.02 | C_16_H_17_O_9_ | [M-H]^-^ | 353.08728 | 353.0867 | 0.571 | MS^2^: 190.93822, 178.94182, 134.93448 | 4-Caffeoylquinic acid^(2)^ | LJH |
| 98 | 12.02 | C_16_H_13_O_5_ | [M+H]^+^ | 285.07578 | 285.07575 | -0.460 | MS^2^: 270.03680, 252.96262, 224.95395  MS^3^[284.97424]: 213.87849, 136.77293 | Genkwanin^(2)^ | LJH |
| 99* | 12.17 | C_21_H_19_O_11_ | [M-H]^-^ | 447.09323 | 447.09218 | 1.042 | MS^2^: 327.01169, 283.93683, 254.91034  MS^3^[283.93683]: 254.93408, 227.04910 | Quercetin | LJH |
| 100 | 12.60 | C_21_H_19_O_10_ | [M-H]^-^ | 431.0983 | 431.09727 | 2.382 | MS^2^: 268.98849  MS^3^[268.98849]: 224.98120, 182.88109, 158.87399 | Cosmosiin^(27)^ | CPN |
| 101 | 12.86 | C_28_H_32_O_15_ | [M-H]^-^ | 609.18188 | 609.18139 | 1.007 | MS^2^: 301.01581  MS^3^[301.01581]: 286.05563, 242.02917, 198.91330, 124.80437 | Hesperidin^(28)^ | PSL |
| 102 | 13.04 | C_25_H_25_O_13_ | [M+H]^+^ | 533.12811 | 533.12896 | -0.857 | MS^2^: 285.06866 MS^3^[285.06866]: 269.98666, 252.94070, 224.96426, 136.95380 | Calycosin-7-O-Glc-6"-O-malonate^(4)^ | AMB |
| 103 | 13.22 | C_30_H_37_O_15_ | [M-H]^-^ | 637.21320 | 637.21269 | 0.503 | MS^2^: 491.25458, 461.20081  MS^3^[461.20081]: 315.21716, 296.90900, 178.91124, 134.95050 | Plantainoside C^(29)^ | PSL |
| 104 | 13.15 | C_25_H_23_O_12_ | [M-H]^-^ | 515.11920 | 515.1184 | 1.548 | MS^2^: 353.08929, 299.16864, 255.11386  MS^3^[353.08929]: 216.81763, 190.91092, 178.83662, 172.87747 | Cynarin^(2)^ | LJH |
| 105 | 13.37 | C_12_H_15_O_3_ | [M+H]^+^ | 207.10181 | 207.10118 | 0.239 | MS^2^: 188.91054, 179.01219, 164.83308, 150.91985, 132.97295  MS^3^[188.91054]: 170.97398, 160.83101, 118.96529, 104.88371 | Senkyunolide F(23) | LWF |
| 106 | 13.51 | C_20_H_28_O_8_Na | [M+Na]^+^ | 419.168 | 419.16763 | 0.361 | MS^2^: 257.04166, 240.96881 | Lobetyolin^(1)^ | CPN |
| 107 | 13.62 | C_28_H_32_O_15_ | [M-H]^-^ | 609.18188 | 609.18139 | 0.302 | MS^2^: 489.11548, 343.06464, 301.00073, 286.00299  MS^3^[301.00073]: 285.96307, 257.02457, 198.97754, 124.77251 | Neohesperidin^(28)^ | PSL |
| 108 | 13.96 | C_26_H_21_O_10_ | [M-H]^-^ | 493.11401 | 493.11386 | 1.087 | MS^2^: 313.10291, 295.02203  MS^3^[295.02203]: 280.02005, 276.95142, 158.93004, 108.83672 | Salvianolic acid A^(3)^ | SMB |
| 109 | 14.09 | C_36_H_37_O_20_ | [M-H]^-^ | 789.18799 | 789.18726 | 0.720 | MS^2^: 609.08655, 473.19141, 301.01843  MS^3^[609.08655]: 300.95557, 271.01129, 254.82736, 178.95930 | Leonurusoide F^(30)^ | LJH |
| **NO.** | **RT**  **min** | **Experical Formula** | **Proposal Ions** | **Experimental  Mass m/z** | **Theoretical Mass m/z** | **Mass Error (ppm)** | **MS2/MS3** | **Identification** | **Crude drug** |
| 110 | 14.12 | C_36_H_37_O_20_ | [M-H]^-^ | 789.18805 | 789.18726 | 0.780 | MS^2^: 609.09552, 473.06219, 301.05981  MS^3^[609.09552]: 300.97559, 271.08499, 254.91943, 178.97952 | 3'''-syringylrutin^(2)^ | LJH |
| 111 | 14.29 | C_36_H_37_O_20_ | [M-H]^-^ | 789.18774 | 789.18726 | 0.470 | MS^2^: 609.06842, 473.05975, 301.05188  MS^3^[609.06842]: 301.07678, 271.01831, 178.94933 | Leonurusoide E^(30)^ | LJH |
| 112 | 14.33 | C_36_H_37_O_20_ | [M-H]^-^ | 789.18774 | 789.18726 | 0.470 | MS^2^: 609.21692, 473.31781, 301.02380  MS^3^[609.21692]: 300.99188, 271.02713, 178.96500 | 2'''-syringylrutin^(2)^ | LJH |
| 113 | 14.38 | C_12_H_15_O_3_ | [M+H]^+^ | 207.10184 | 207.10136 | 0.269 | MS^2^: 207.05766, 188.92465, 164.82222  MS^3^[188.92465]: 170.94589, 160.97383, 145.90746, 118.91316 | 4-hydroxy-3-butylphthalide^(31)^ | LWF |
| 114 | 14.65 | C_10_H_11_O_3_ | [M-H]^-^ | 179.07129 | 179.07027 | 1.019 | MS^2^: 178.89275, 107.75107, 108.83372, 90.84857, 80.86002, 64.61841 | Coniferyl alcohol^(32)^ | LJH |
| 115 | 14.86 | C_24_H_45_N_4_O_4_ | [M+H]^+^ | 453.3418 | 453.34366 | 0.128 | MS^2^: 444.47650, 435.49396, 387.82239, 341.21997  MS^3^[444.47650]: 435.41656, 387.92203, 322.22906, 209.08455 | 1,8,15,22-Tetraza-2,9,16,23-  cyclooctacosanetetrone | LJH |
| 116 | 15.15 | C_36_H_29_O_16_ | [M-H]^-^ | 717.14551 | 717.14501 | 0.499 | MS^2^: 519.06494, 321.08618  MS^3^[519.06494]: 339.01563, 320.96075, 295.10773, 278.95142 | Salvianolic acid E^(3)^ | SMB |
| 117 | 15.23 | C_36_H_29_O_16_ | [M-H]^-^ | 717.14551 | 717.14501 | 0.499 | MS^2^: 699.48822, 673.22211, 537.06763, 519.10223, 339.04953  MS^3^[519.10223]: 338.94934, 320.99744, 295.09860, 278.96234 | Salvianolic acid B^(3)^ | SMB |
| 118 | 15.30 | C_36_H_29_O_16_ | [M-H]^-^ | 717.14551 | 717.14501 | 0.499 | MS^2^: 519.04944, 338.98181, 320.99976  MS^3^[519.04944]: 338.95630, 321.05243, 295.10126, 279.01611 | Isosalvianolic acid E^(3)^ | SMB |
| 119 | 16.30 | C_24_H_27_O_12_ | [M+COOH]^-^ | 507.15048 | 507.1497 | 1.533 | MS^2^: 461.05109, 327.13107, 299.12015, 283.08948, 196.92448, 178.85419 | (6aR,11aR)9,10-Dimethoxypterocarpan- 3-O-b-D-glucoside^(4)^ | AMB |
| 120 | 16.40 | C_36_H_37_O_19_ | [M-H]^-^ | 773.19305 | 773.19235 | 0.695 | MS^2^: 607.18140, 593.13666, 575.21655, 473.08533, 285.00519 | Leonurusoide C^(30)^ | LJH |
| 121 | 16.42 | C_36_H_37_O_19_ | [M-H]^-^ | 773.19287 | 773.19235 | 0.515 | MS^2^: 607.13708, 593.18182, 575.10114, 473.17651, 284.94412 | Leonurusoide D^(30)^ | LJH |
| 122 | 16.46 | C_36_H_37_O_19_ | [M-H]^-^ | 773.19269 | 773.19235 | 0.335 | MS^2^: 607.15204, 593.22919, 575.22333, 473.14709, 284.93439 | Leonurusoide A^(30)^ | LJH |
| 123 | 16.50 | C_36_H_37_O_19_ | [M-H]^-^ | 773.19287 | 773.19235 | 0.515 | MS^2^: 607.10431, 593.22552, 575.15564, 473.24298, 284.87891 | Leonurusoide B^(30)^ | LJH |
| 124 | 16.65 | C_36_H_29_O_16_ | [M-H]^-^ | 717.1463 | 717.14501 | 1.289 | MS^2^: 519.08405, 321.03018  MS^3^[519.08405]: 339.03711, 320.96136, 295.04099, 279.01443 | Isosalvianolic acid B^(3)^ | SMB |
| 125 | 17.15 | C_23_H_28_O_10_Na | [M+Na]^+^ | 487.15668 | 487.15746 | -1.618 | MS^2^: 469.37192, 325.07513  MS^3^[469.37192]: 303.15771, 158.14626 | 7,2'-dihydroxy-3',4'- dimethoxyisoflavan-7-O-β-D-glucoside^(4)^ | AMB |
| 126 | 17.25 | C_16_H_11_O_5_ | [M-H]^-^ | 283.06113 | 283.06009 | 1.030 | MS^2^: 267.97919  MS^3^[267.97919]: 239.97380, 210.98735, 194.89156, 184.00952 | Calycosin^(19)^ | AMB |
| **NO.** | **RT**  **min** | **Experical Formula** | **Proposal Ions** | **Experimental  Mass m/z** | **Theoretical Mass m/z** | **Mass Error (ppm)** | **MS2/MS3** | **Identification** | **Crude drug** |
| 126 | 17.25 | C_16_H_13_O_5_ | [M+H]^+^ | 285.07608 | 285.07575 | 0.330 | MS^2^: 269.97751, 253.04736, 224.95935, 136.83463  MS^3^[269.97751]: 252.94832, 241.95605, 136.83795 | Calycosin^(19)^ | AMB |
| 127 | 17.32 | C_37_H_31_O_16_ | [M-H]^-^ | 731.16119 | 731.16066 | 0.529 | MS^2^: 533.09869  MS^3^[533.09869]: 353.04285, 335.00006, 309.09821 | Methyl salvianolic acid B^(22)^ | SMB |
| 128 | 17.79 | C_25_H_25_O_12_ | [M+H]^+^ | 517.13434 | 517.13171 | 0.287 | MS^2^: 473.24170, 431.02972, 269.01874 MS^3^[269.01874]: 253.98140, 237.01953, 212.91338 | Formononetin-7-O-Glc-6"-O-malonate^(4)^ | AMB |
| 129 | 17.95 | C_30_H_25_O_13_ | [M-H]^-^ | 593.12982 | 593.12896 | 1.438 | MS^2^: 447.20825, 284.93542  MS^3^[284.93542]: 267.04181, 257.02991, 240.99872, 150.78917 | Apigenin-7-caffeoylglucoside^(2)^ | LJH |
| 130 | 18.61 | C_30_H_25_O_13_ | [M-H]^-^ | 593.13007 | 593.12896 | 1.859 | MS^2^: 447.15497, 285.05200  MS^3^[285.05200]: 266.94772, 256.97180, 241.10278, 150.88774 | Kaempferol-7-coumaroylglucoside^(2)^ | LJH |
| 131 | 18.69 | C_26_H_28_O_13_Na | [M+H]^+^ | 571.14227 | 571.14221 | 0.102 | MS^2^: 527.15686, 485.14648  MS^3^[527.15686]: 512.27228, 485.11554, 323.09955, 226.78573 | (6aR,11aR)9,10-Dimethoxypterocarpan-3-O-β-D-gluc  oside-6''-O-malonate^(4)^ | AMB |
| 132 | 18.69 | C_17_H_17_O_5_ | [M+H]^+^ | 301.10745 | 301.10645 | 0.400 | MS^2^: 268.97443, 190.86771, 166.90765  MS^3^[166.90765]: 151.84372, 133.87637, 106.79704 | 3-Hydro-9,10-diMP^(19)^ | AMB |
| 133 | 19.52 | C_26_H_30_O_13_Na | [M+Na]^+^ | 573.15814 | 573.15786 | 0.966 | MS^2^: 529.21289, 487.23016 MS^3^[529.21289]: 514.11914, 349.12860 | 2'-Hydroxy-3',4'-dimethoxyisoflavan-7-O-b-D-glucoside-6''-O-malonate^(1)^ | AMB |
| 134 | 19.69 | C_30_H_25_O_12_ | [M-H]^-^ | 577.13464 | 577.13405 | 1.018 | MS^2^: 268.96106, 252.27008, 225.12677 | Apigenin-7-(3'''-coumaroyl)-galactopyranoside^(2)^ | LJH |
| 135 | 19.84 | C_31_H_33_O_14_ | [M+COOH]^-^ | 629.1861 | 629.18648 | -0.382 | MS^2^: 583.01477, 552.97443, 535.27118, 431.23822  MS^3^[552.97443]: 431.11432, 413.03375, 309.19891, 164.92958 | Benzoylpaeoniflorin^(5)^ | PLP |
| 136 | 19.88 | C_31_H_33_O_14_ | [M+COOH]^-^ | 629.1861 | 629.18616 | -0.322 | MS^2^: 582.94141, 553.09729, 535.08246, 431.19788  MS^3^[553.09729]: 431.09283, 413.12756, 309.13220, 164.93619 | Benzoylalbiflorin^(5)^ | PLP |
| 137* | 20.05 | C_15_H_9_O_5_ | [M-H]^-^ | 269.0452 | 269.04444 | 0.750 | MS^2^: 224.95103, 200.88519, 150.74980, 116.85899  MS^3^[224.95103]: 210.02858, 197.01891, 180.90877, 168.90672, 144.75797 | Apigenin^(19)^ | AMB |
| 138 | 20.19 | C_30_H_25_O_12_ | [M-H]^-^ | 577.13464 | 577.13405 | 1.018 | MS^2^: 533.20392, 461.26410, 268.94550 | Apigenin-7-(2'''-coumaroyl)-galactopyranoside^(2)^ | LJH |
| 139 | 20.56 | C_18_H_31_O_5_ | [M-H]^-^ | 327.2186 | 327.21704 | 0.439 | MS^2^: 309.14096, 291.23709, 229.15179, 211.00562, 208.98438, 126.94287 | 9,12,13-Trihydroxy-10,15-octadecadienoic acid^(33)^ | SMB |
|  |  |  |  |  |  |  |  |  |  |
| **NO.** | **RT**  **min** | **Experical Formula** | **Proposal Ions** | **Experimental  Mass m/z** | **Theoretical Mass m/z** | **Mass Error (ppm)** | **MS2/MS3** | **Identification** | **Crude drug** |
| 140 | 20.84 | C_16_H_13_O_6_ | [M+H]^+^ | 301.07114 | 301.07066 | 0.475 | MS^2^: 285.97455, 268.97519, 241.01184, 166.91736  MS^3^[285.97455]: 257.90619 | 3,4',5-Trihydroxy-7-methoxyflavone^(19)^ | AMB |
| 141 | 21.16 | C_47_H_78_O_19_Na | [M+Na]^+^ | 969.50079 | 969.50295 | -2.161 | MS^2^: 941.40692, 924.58417, 898.40820, 856.44940, 801.41370, 730.41370  MS^3^[941.40692]: 924.54565, 844.44653, 658.43097, 562.38623, 545.53864 | Astragaloside V/Astragaloside VI  /Astragaloside VII^(4)^ | AMB |
| 142 | 21.41 | C_20_H_33_O_5_ | [M+H]^+^ | 353.23001 | 353.23225 | -2.241 | MS^2^: 335.13568, 317.07648, 235.13986  MS^3^[335.13568]: 317.29605, 298.99274, 270.48029, 256.36700 | 15-epileopersin C | LJH |
| 143 | 21.43 | C_18_H_33_O_5_ | [M-H]^-^ | 329.23276 | 329.23225 | 1.547 | MS: 311.17511, 293.10687, 229.01620, 210.95200, 170.96060 | Tianshic acid^(15)^ | CPN |
| 144 | 21.84 | C_16_H_13_O_4_ | [M+H]^+^ | 269.08011 | 269.08121 | -0.025 | MS^2^: 254.02425, 226.04738, 224.87726, 196.96504, 137.95897, 132.79402  MS^3^[254.02425]: 237.04469, 225.96039, 135.79353, 117.88737, 107.73196 | Formononetin^(4)^ | AMB |
| 144 | 21.84 | C_16_H_11_O_4_ | [M-H]^-^ | 267.06592 | 267.06518 | 0.735 | MS^2^: 252.05939  MS^3^[252.05939]: 222.93463, 207.94168, 194.91460, 131.77985 | Formononetin^(4)^ | AMB |
| 145* | 21.87 | C_42_H_69_O_16_ | [M+COOH]^-^ | 829.45831 | 829.45801 | 0.298 | MS^2^: 783.42084, 621.23438, 586.03680, 489.44446, 453.50983 | Astragaloside IV^(4)^ | AMB |
| 146 | 21.90 | C_42_H_69_O_16_ | [M+COOH]^-^ | 829.45844 | 829.45801 | 0.428 | MS^2^: 783.43158, 621.30438, 489.43640 | Isoastragaloside IV^(4)^ | AMB |
| 147 | 22.00 | C_41_H_68_O_14_Na | [M+Na]^+^ | 807.45111 | 807.45012 | 0.982 | MS^2^: 645.42499, 495.33771, 335.10074  MS^3^[495.33771]: 477.39056, 439.04111 | Astragaloside Ш^(4)^ | AMB |
| 147 | 22.00 | C_42_H_69_O_16_ | [M+COOH]^-^ | 829.45801 | 829.45844 | 0.428 | MS^2^: 783.44733, 621.30255  MS^3^[783.44733]: 621.29266, 489.38165, 383.34827 | Astragaloside Ш^(4)^ | AMB |
| 148 | 22.06 | C_23_H_33_O_6_ | [M+H]^+^ | 405.22504 | 405.22716 | -2.125 | MS^2^: 387.16483, 331.14825, 295.13858 | (-)-Gomisin Ki | LJH |
| 149 | 22.14 | C_16_H_11_O_6_ | [M-H]^-^ | 299.05563 | 299.05501 | 2.058 | MS^2^: 283.95175  MS^3^[283.95175]: 269.13748, 255.97385, 150.96512 | 3-methyl-kaempferol^(2)^ | LJH |
| 150 | 22.18 | C_9_H_11_O_3_ | [M+H]^+^ | 167.07054 | 167.07008 | 0.269 | MS^2^: 151.82538, 133.81618, 125.68007  MS^3^[151.82538]: 133.85056, 123.89526, 105.79950 | Ethylparaben^(34)^ | LH |
| 151 | 22.18 | C_16_H_13_O_6_ | [M+H]^+^ | 301.07114 | 301.07066 | 1.579 | MS^2^: 285.97455, 257.92542, 241.01184, 166.91736  MS^3^[285.97455]: 257.90619 | 7-O-Methyl-luteolin | AMB |
| **NO.** | **RT**  **min** | **Experical Formula** | **Proposal Ions** | **Experimental  Mass m/z** | **Theoretical Mass m/z** | **Mass Error (ppm)** | **MS2/MS3** | **Identification** | **Crude drug** |
| 152 | 22.33 | C_20_H_33_O_5_ | [M+H]^+^ | 353.23047 | 353.23225 | -1.781 | MS^2^: 335.20697, 317.20441, 235.02106 | Leopersin C | LJH |
| 153 | 22.50 | C_9_H_11_O_3_ | [M+H]^+^ | 167.07045 | 167.07008 | 0.179 | MS^2^:151.87575, 133.77858, 125.78311  MS^3^[151.87575]: 133.77870, 123.98038, 105.82416 | Paeonol^(35)^ | PLP |
| 154 | 22.63 | C_18_H_33_O_5_ | [M-H]^-^ | 329.23441 | 329.23257 | 0.319 | MS^2^: 311.16412, 293,20490  MS^3^[311.16412]: 293.19083, 275.23853, 265.25003, 210.92642, 195.11125 | 9,12,13-Trihydroxy-10-octadecenoic acid^(33)^ | SMB |
| 155 | 22.66 | C_48_H_77_O_18_ | [M-H]^-^ | 941.51025 | 941.51044 | -0.192 | MS^2^: 923.51837, 795.52307, 615.53735, 597.40985  MS^3^[923.51837]: 879.54315, 733.34430, 597.10815, 525.57257, 457.52844 | Soyasaponin I^(4)^ | AMB |
| 156 | 22.71 | C_44_H_71_O_17_ | [M+COOH]^-^ | 871.46869 | 871.46857 | 0.113 | MS^2^: 825.38519, 765.41064, 603.52240  MS^3^[825.38519]: 783.46503, 765.32544, 717.40686 | Astragaloside II^(4)^ | AMB |
| 157 | 23.02 | C_12_H_11_O_3_ | [M-H]^-^ | 203.07227 | 203.07109 | 0.819 | MS^2^: 202.93361, 173.90796, 159.82129  MS^3^[202.93361]: 159.98242, 131.78369 | 3-butylidene-4- hydroxyphthalide^(31)^ | ASD |
| 158 | 23.25 | C_44_H_71_O_17_ | [M+COOH]^-^ | 871.46869 | 871.46857 | 0.113 | MS^2^: 825.32172, 783.27167, 765.54047, 603.37964 | Isoastragaloside II^(4)^ | AMB |
| 159 | 23.43 | C_46_H_73_O_18_ | [M+COOH]^-^ | 913.47919 | 913.47914 | 0.048 | MS^2^: 867.32526, 807.46326, 765.46313 | Isoastragaloside I^(4)^ | AMB |
| 160^*^ | 23.17 | C_16_H_11_O_5_ | [M-H]^-^ | 283.06009 | 283.061 | -0.460 | MS^2^: 267.91132, 255.06107 | Wogonin^(23)^ | AMB |
| 160^*^ | 23.17 | C_16_H_13_O_5_ | [M+H]^+^ | 285.07575 | 285.07529 | 0.900 | MS^2^: 270.05731, 257.07001, 242.05884, 224.94849, 166.86272 | Wogonin^(23)^ | AMB |
| 161 | 23.62 | C_12_H_11_O_3_ | [M-H]^-^ | 203.07231 | 203.07115 | 0.879 | MS^2^: 173.90776, 158.94014, 129.80663 | Senkyunolide E^(24)^ | LWF |
| 162 | 23.72 | C_19_H_19_O_4_ | [M+H]^+^ | 311.12793 | 311.12778 | 0.144 | MS^2^: 283.01563, 267.06351, 225.01590  MS^3^[267.06351]: 252.01366, 238.96619, 224.91170, 184.96959 | Tanshinone IIB^(3)^ | SMB |
| 163 | 23.72 | C_19_H_19_O_5_ | [M-H]^-^ | 327.12494 | 327.12344 | 0.56 | MS^2^: 283.09772, 239.12790, 201.07126  MS^3^[283.09772]: 239.10068, 227.03650, 212.13113 | Phenanthro[1,2-b]furan-10,11-dione, 1,2,6,7,8,9-hexahydro-7(8 or 9)-hydroxy-1  -(hydroxymethyl)-6,6-dimethyl-, (1S)-^(3)^ | SMB |
| 164 | 23.95 | C_18_H_29_O_4_ | [M-H]^-^ | 309.20685 | 309.20603 | 0.814 | MS^2^: 291.08136, 208.97046, 184.99191  MS^3^[291.08136]: 273.12488, 206.93744, 124.96771, 120.89093 | 6-Methylgingediol^(15)^ | CPN |
| 165 | 24.07 | C31H47O5 | [M-H]- | 499.3429 | 499.3418 | 1.099 | MS^2^: 481.30252, 453.31253, 437.31018, 419.28976, 325.28168  MS^3^[419.32962]: 403.22052, 389.36816 | 15α-Hydroxydehydrotumulosic acid(36) | PCW |
| **NO.** | **RT**  **min** | **Experical Formula** | **Proposal Ions** | **Experimental  Mass m/z** | **Theoretical Mass m/z** | **Mass Error (ppm)** | **MS2/MS3** | **Identification** | **Crude drug** |
| 166 | 24.10 | C_12_H_17_O_2_ | [M+H]^+^ | 193.12257 | 193.1223 | 0.264 | MS^2^: 174.87056, 146.89253, 136.85953, 104.82687  MS^3^[146.89253]: 118.80200, 104.84105, 90.79341, 80.81168, 66.75367 | Senkyunolide A^(5)^ | LWF，ASD |
| 167 | 24.10 | C_15_H_19_O_2_ | [M+H]^+^ | 231.13832 | 231.13795 | 0.364 | MS^2^: 212.98607, 203.00772, 184.90681, 156.91339  MS^3^[184.90681]: 169.91766, 156.94160, 142.91541, 128.81763 | Dehydrocostuslactone^(37)^ | AMK |
| 168 | 24.18 | C_20_H_31_O_4_ | [M+H]^+^ | 335.21957 | 335.22168 | -2.116 | MS^2^: 317.20966, 247.15854, 242.97479, 225.08325 | (Iso)preleoheterin^(38)^ | LJH |
| 169 | 24.19 | C_18_H_31_O_4_ | [M-H]^-^ | 311.22241 | 311.22168 | 0.724 | MS^2^: 293.09964, 275.21597, 235.09586, 223.08899, 155.04681  MS^3^[293.09964]: 275.21918, 235.16925, 209.06140 | 8-Nonenoic acid(15) | CPN |
| 170 | 24.24 | C_46_H_73_O_18_ | [M+COOH]^-^ | 913.47937 | 913.47914 | 0.468 | MS^2^: 867.28363, 807.39630, 765.45233  MS^3^[867.28363]: 807.47284, 765.43329, 747.48840, 645.23669, 633.39984 | Astragaloside I^(4)^ | AMB |
| 171 | 24.46 | C_19_H_17_O_4_ | [M+H]^+^ | 309.11258 | 309.11213 | 0.444 | MS^2^: 265.00131  MS^3^[265.00131]: 250.02563, 247.03606, 222.94188, 194.90242 | Tanshinaldehyde^(3)^ | SMB |
| 172 | 24.47 | C_18_H_15_O_4_ | [M-H]^-^ | 295.09747 | 295.09648 | 0.985 | MS^2^: 277.03091, 265.03012, 237.92670 MS^3^[265.03012]: 236.98048, 222.08087 | Tanshinone Ⅵ^(22)^ | SMB |
| 173 | 24.55 | C_18_H_15_O_4_ | [M-H]^-^ | 295.09744 | 295.09648 | 0.955 | MS^2^: 277.03369, 264.99390, 249.11737, 237.16531 | przewaquinone B^(39)^ | SMB |
| 174 | 24.60 | C_18_H_33_O_4_ | [M-H]^-^ | 313.23788 | 313.23733 | 0.544 | MS^2^: 295.17523, 277.09305, 195.00076, 183.02774  MS^3^[295.17523]: 277.14178, 251.27213, 195.10513, 179.13011 | 2-[4-(2-hydroxy-ethoxy)-1-isobutyl-1,4,6-trimethyl-hept-2-ynyloxy]-ethanol^(6)^ | AMK |
| 175 | 24.60 | C_20_H_33_O_4_ | [M+H]^+^ | 337.23547 | 337.23733 | -1.866 | MS^2^: 319.12396, 224.98059, 206.88031, 152.88058 | Leoheteronone B(40) | LJH |
| 176* | 24.73 | C_30_H_47_O_5_ | [M-H]^-^ | 487.34274 | 487.3418 | 0.939 | MS^2^: 469.27365, 389.30234, 371.30530  MS^3^[469.27365]: 451.15829, 423.32919, 407.38803, 367.24744 | Tormentic acid^(3)^ | SMB |
| 177 | 24.76 | C_31_H_45_O_6_ | [M-H]^-^ | 513.32227 | 513.32106 | 1.204 | MS^2^: 481.32983  MS^3^[481.32983]: 466.30524, 421.42932, 403.36624 | 5α,8α-peroxydehydrotumulosic acid^(41)^ | PCW |
| 178 | 24.78 | C_48_H_74_O_19_Na | [M+Na]^+^ | 977.47394 | 977.47165 | 2.342 | MS^2^: 933.42950, 891.57025, 873.37946, 693.41779, 657.43170, 477.37054,  MS^3^[933.42950]: 891.45746, 873.47760, 753.49249, 657.40399, 343.26492 | Malonylastragaloside I^(42)^ | AMB |
| 179 | 24.80 | C_48_H_75_O_19_ | [M+COOH]^-^ | 955.4881 | 955.4897 | -1.606 | MS^2^: 911.19147, 849.42175, 765.38281, | Acetylastragaloside I^(42)^ | AMB |
| 180 | 24.94 | C_31_H_45_O_5_ | [M-H]^-^ | 497.32748 | 497.32615 | 1.329 | MS^2^: 479.37384, 435.35376, 419.33102, 401.25888  MS^3^[419.33102]: 403.32584, 389.34207 | 16α,25-Dihydroxydehydroeburiconic acid | PCW |
| **NO.** | **RT**  **min** | **Experical Formula** | **Proposal Ions** | **Experimental  Mass m/z** | **Theoretical Mass m/z** | **Mass Error (ppm)** | **MS2/MS3** | **Identification** | **Crude drug** |
| 181 | 24.99 | C_20_H_33_O_4_ | [M+H]^+^ | 337.23505 | 337.23733 | -2.286 | MS^2^: 319.18903, 224.81381, 207.02382 | 15-epileoheteronone B^(40)^ | LJH |
| 182 | 25.01 | C_18_H_33_O_4_ | [M-H]^-^ | 313.23801 | 313.23733 | 0.674 | MS^2^: 295.12756, 277.19894, 195.04443, 179.02962  MS^3^[295.12756]: 277.16840, 251.19040, 195.08788, 179.05804, 112.77705 | 9,10-dihidroxy-12-octadecenoic acid | SMB |
| 183 | 25.01 | C_20_H_33_O_4_ | [M+H]^+^ | 337.23489 | 337.23733 | -2.446 | MS^2^: 319.33945, 224.92633, 207.07021, 152.98279 | Leoheteronone D^(40)^ | LJH |
| 184 | 25.15 | C_20_H_33_O_4_ | [M+H]^+^ | 337.23523 | 337.23733 | -2.106 | MS^2^: 319.15741, 224.97751, 207.03528, 152.81842 | 15-epileoheteronone D^(40)^ | LJH |
| 185 | 25.47 | C_18_H_31_O_4_ | [M-H]^-^ | 311.22253 | 311.22168 | 0.844 | MS^2^: 293.19696, 275.16724, 235.05298  MS^3^[293.19696]: 275.18207, 265.19080, 249.26746, | 2-Nonenoic acid^(15)^ | CPN |
| 186 | 25.53 | C_18_H_15_O_3_ | [M+H]^+^ | 279.10175 | 279.10157 | 0.179 | MS^2^: 260.99155, 233.00015, 209.09488 | 15,16-dihydrotanshinone I^(3)^ | SMB |
| 187 | 25.57 | C_12_H_15_O_2_ | [M+H]^+^ | 191.10658 | 191.10665 | -0.076 | MS^2^: 191.10625, 172.94191, 148.89799, 134.99260, 106.83734, 78.76982  MS^3^[172.94191]: 155.02434, 144.98326, 116.75432 | (Z)-Ligustilide^(5)^ | ASD LWF |
| 188 | 25.69 | C_27_H_46_O_12_P | [M-H]^-^ | 593.27319 | 593.27213 | 1.770 | MS^2^: 413.16260, 315.02710, 277.13943, 240.98178 | PI^(2)^ | LJH |
| 189 | 25.96 | C_15_H_21_O_2_ | [M+H]^+^ | 233.15399 | 233.1536 | 0.384 | MS^2^: 214.99947, 186.93704, 150.91469 | Isoasterolide A^(6)^ | AMK |
| 190 | 26.01 | C_18_H_17_O_3_ | [M+H]^+^ | 281.11758 | 281.11722 | 0.359 | MS^2^: 263.03845, 253.02957, 234.99045  MS^3^[263.03845]: 247.94057, 235.01265, 221.01555 | Trijuganone B^(22)^ | SMB |
| 191 | 26.04 | C_19_H_21_O_4_ | [M-H]^-^ | 313.14343 | 313.14426 | 0.824 | MS^2^: 295.02768, 283.05545  MS^3^[283.05545]: 255.09613, 240.13446 | Neocryptotanshinone^(3)^ | SMB |
| 192 | 26.1 | C_18_H_35_O_4_ | [M+COOH]^-^ | 315.25363 | 315.25298 | 0.644 | MS^2^: 297.17523  MS^3^[297.17523]: 279.24451, 170.98938, 154.94315 | Methylhexadecanoate^(43)^ | LWF |
| 193* | 26.15 | C_15_H_21_O_2_ | [M+H]^+^ | 233.15355 | 233.1536 | -0.056 | MS^2^: 215.03506, 187.00481, 150.90129, 144.85855, 130.91187, 104.96098  MS^3^[215.03506]: 196.97169, 186.98859, 172.92264, 158.94539 | AtractylenolideⅡ^(1)^ | CPN |
| 194 | 26.29 | C_18_H_15_O_3_ | [M+H]^+^ | 279.10199 | 279.10157 | 0.419 | MS^2^: 261.10596, 233.12564, 208.98724 | Dihydrotanshinone I^(3)^ | SMB |
| 195 | 26.36 | C_27_H_45_O_11_S | [M-H]^-^ | 577.26868 | 577.2677 | 1.682 | MS^2^: 298.99890, 277.12610, 224.89122, 164.91563 | SQDG^(2)^ | LJH |
| 196 | 26.70 | C_31_H_46_NO_4_ | [M+H]^+^ | 496.33929 | 496.34213 | -2.845 | MS^2^: 478.33575, 183.92455  MS^3^[478.33575]: 419.30371, 283.35837, 162.97028 | 7-[4-(11-hydroxy-undecyloxy)-phenyl]-7-pyridin-3-yl-hept-6-enoic acid ethyl ester^(6)^ | AMK |
| 197 | 26.89 | C_31_H_45_O_4_ | [M-H]^-^ | 481.33218 | 481.33123 | 1.960 | MS^2^: 466.25827, 421.31210, 407.16382 | Poricoic acid C^(44)^ | PCW |
| 198 | 26.92 | C_33_H_49_O_6_ | [M-H]^-^ | 541.35278 | 541.35236 | 0.765 | MS^2^: 497.32709, 495.36334, 481.28058, 479.22998, 419.28198 | 29-hydroxydehydropachymic acid^(41)^ | PCW |
| **NO.** | **RT**  **min** | **Experical Formula** | **Proposal Ions** | **Experimental  Mass m/z** | **Theoretical Mass m/z** | **Mass Error (ppm)** | **MS2/MS3** | **Identification** | **Crude drug** |
| 199 | 27.11 | C_18_H_31_O_3_ | [M-H]^-^ | 295.22778 | 295.22677 | 1.009 | MS^2^: 277.14224, 194.97821, 178.96712, 170.87033 | Coronaric acid^(15)^ | CPN |
| 200* | 27.18 | C_19_H_21_O_3_ | [M+H]^+^ | 297.14877 | 297.14852 | 0.249 | MS^2^: 279.04266, 253.98929  MS^3^[279.04266]: 263.99411, 251.12540, 237.03571, 169.01627 | Cryptotanshinone^(3)^ | SMB |
| 201 | 27.23 | C_20_H_27_O_2_ | [M-H]^-^ | 299.2012 | 299.20055 | 0.643 | MS^2^: 283.11954, 226.98663, 214.03220  MS^3^[226.98663]: 212.09746, 183.98355 | Hispanone^(2)^ | LJH |
| 202 | 27.3 | C_31_H_47_O_4_ | [M-H]^-^ | 483.34799 | 483.34688 | 1.104 | MS^2^: 437.36240, 421.40634, 405.31644, 390.30386, 337.26486 | Dehydrotumulosic acid^(41)^ | PCW |
| 203 | 27.35 | C_19_H_21_O_3_ | [M+H]^+^ | 297.14856 | 297.14852 | 0.039 | MS^2^: 279.00250, 254.06879  MS^3^[279.00250]: 263.93915, 251.01001, 237.01672, 223.13068 | Isocryptotanshinone^(3)^ | SMB |
| 204 | 27.46 | C_12_H_15_O_2_ | [M+H]^+^ | 191.10625 | 191.10665 | -0.406 | MS^2^: 172.90623, 162.87242, 148.87233, 134.97240, 106.91415, 78.81138  MS^3^[172.90623]: 154.95393, 144.87309, 129.85947, 116.92865 | (E)-Ligustilide^(5)^ | ASD  LWF |
| 205* | 27.48 | C_15_H_19_O_2_ | [M+H]^+^ | 231.13806 | 231.13795 | 0.104 | MS^2^: 203.01448, 189.07362, 184.90160, 160.97501, 156.86913  MS^3^[184.90160]: 170.01311, 156.94145, 142.80702, 128.82561, 104.94806 | AtractylenolideⅠ^(1)^ | CPN |
| 206 | 27.55 | C_31_H_49_O_4_ | [M-H]^-^ | 485.36368 | 485.36253 | 2.356 | MS^2^: 467.31027, 449.41302, 441.35294, 439.31497, 425.46097, 423.35233 MS^3^[423.35233]: 421.47665, 407.36411, 313.29230 | Tumulosic acid^(41)^ | PCW |
| 207 | 27.96 | C_18_H_29_O_3_ | [M-H]^-^ | 293.21198 | 293.21112 | 0.889 | MS2: 275.24976, 249.09033, 208.91653, 184.96841, 140.99445, 124.96745 | 9-Oxo-10E,12Z-octadecadienoic acid(3) | SMB |
| 208 | 28.09 | C_46_H_59_O_6_S_2_ | [M+H]^+^ | 771.37793 | 771.37475 | 3.173 | MS^2^: 669.32593, 455.25031, 441.17584, 353.12482 | 6-(2-octyloxy-propionyloxy)-3,8-dithia-cyclopenta[a]indene-2-carboxylic acid 4'-dodecyloxy-biphenyl-4-yl ester^(6)^ | AMK |
| 209 | 28.14 | C_33_H_51_O_6_ | [M-H]^-^ | 543.36871 | 543.36801 | 1.278 | MS^2^: 525.27527, 497.32434, 481.37299, 467.33618, 465.42670, 355.32697 | 25-hydroxypachymic acid^(45)^ | PCW |
| 210 | 28.29 | C_31_H_45_O_4_ | [M-H]^-^ | 481.33231 | 481.33123 | 2.231 | MS^2^: 463.28961, 437.47488, 435.42294, 403.23270, 311.19385, 293.20337 | Polyporenic acid C (PAC)^(45)^ | PCW |
| 211 | 28.46 | C_24_H_29_O_4_ | [M+H]^+^ | 381.20627 | 381.20603 | 0.234 | MS^2^: 363.19351, 335.28741, 190.89966  MS^3^[190.89966]: 172.94557, 162.98262, 154.96483, 144.96233 | Riligustilide^(5)^ | ASD，LWF |
|  |  |  |  |  |  |  |  |  |  |
| **NO.** | **RT**  **min** | **Experical Formula** | **Proposal Ions** | **Experimental  Mass m/z** | **Theoretical Mass m/z** | **Mass Error (ppm)** | **MS2/MS3** | **Identification** | **Crude drug** |
| 212 | 28.54 | C_12_H_15_O_2_ | [M+H]^+^ | 191.10625 | 191.10665 | -0.406 | MS^2^: 173.04517, 162.87804 154.83928, 144.94550, 134.90303  MS^3^[173.04517]: 155.06003, 144.90385, 129.91394, 116.92560, 90.86102 | Butylphthalide | ASD  LWF |
| 213 | 28.63 | C_24_H_29_O_4_ | [M+H]^+^ | 381.20593 | 381.20603 | -0.106 | MS^2^: 363.20627, 335.20477, 190.93088  MS^3^[190.93088]: 172.88965, 162.98964, 154.92717, 144.87106 | Tokinolide B^(5)^ | ASD  LWF |
| 214 | 28.7 | C_22_H_44_O_9_P | [M-H]^-^ | 483.27365 | 483.27174 | 3.940 | MS^2^: 255.08780 | PG^(2)^ | LJH |
| 215 | 28.71 | C_31_H_47_O_4_ | [M-H]^-^ | 483.34787 | 483.34688 | 2.035 | MS^2^: 439.39090, 437.46140, 421.31116, 405.32410, 337.31952, 295.13470 | 16α-hydroxyeburiconic acid^(45)^ | PCW |
| 216* | 28.74 | C_24_H_29_O_4_ | [M+H]^+^ | 381.20615 | 381.20603 | 0.114 | MS^2^: 363.13528, 190.97321  MS^3^[190.97321]: 172.94182, 162.88551, 154.89900, 144.91496, 134.82831 | Levistilide A^(5)^ | ASD LWF |
| 217* | 28.86 | C_19_H_19_O_3_ | [M+H]^+^ | 295.13312 | 295.13287 | 0.249 | MS^2^: 277.02783, 266.05096, 249.04050, 234.97763, 221.17557  MS^3^[277.02783]: 262.10181, 249.02155, 231.10379, 221.07034, 191.94714 | Tanshinone IIA^(3)^ | SMB |
| 218 | 28.96 | C_19_H_19_O_3_ | [M+H]^+^ | 295.13312 | 295.13287 | 0.249 | MS^2^: 277.09082, 248.98679, 234.98737  MS^3^[277.09082]: 261.94501, 248.99207, 231.02148, 221.04733 | Isotanshinone IIA^(3)^ | SMB |
| 219 | 29.34 | C_25_H_47_O_11_S | [M-H]^-^ | 555.28424 | 555.28335 | 1.586 | MS^2^: 299.01801, 255.23407, 224.95441, 206.96584, 164.88492 | Almazolone | LJH |
| 220 | 29.47 | C_24_H_29_O_4_ | [M+H]^+^ | 381.20618 | 381.20603 | 0.144 | MS^2^: 335.29721, 190.88525  MS^3^[190.88525]: 172.90472, 163.06718, 154.98695, 134.86925, 116.81750 | Z,Z'-6,6',7,3'a-diligustilide^(23)^ | ASD  LWF |
| 221 | 29.48 | C_33_H_47_O_5_ | [M-H]^-^ | 523.34283 | 523.3418 | 1.966 | MS^2^: 508.35248, 463.28064, 293.14563 | 16α-Acetyloxy-24-methylene-3-oxolanosta-7,9(11)-dien-21oic acid^(45)^ | PCW |
| 222 | 29.62 | C_24_H_29_O_4_ | [M+H]^+^ | 381.20593 | 381.20603 | -0.106 | MS^2^: 363.19409, 190.95100  MS^3^[190.95100]: 172.91945, 162.94324, 154.89626, 134.94942 | 3',6,8',3a-Diligustilide^(23)^ | ASD  LWF |
| 223 | 29.67 | C_22_H_44_O_9_P | [M-H]^-^ | 483.27286 | 483.27174 | 2.305 | MS^2^: 255.13361  MS^3^[255.13361]: 237.14586, 227.04663 | PG^(2)^ | LJH |
| 224 | 29.68 | C_32_H_49_O_5_ | [M-H]^-^ | 513.35828 | 513.35745 | 1.615 | MS^2^: 495.36786, 469.36176, 467.36682, 453.38837, 451.35864 | 3-O-Acetyl-16α-hydroxytrametenolic acid ^(45)^ | PCW |
| 225 | 29.74 | C_16_H_31_O_3_ | [M-H]^-^ | 271.22769 | 271.22677 | 0.919 | MS^2^: 253.21779, 225.15874  MS^3^[225.15874]: 203.13367, 197.15602 | 3-hydroxy-propionic acid tridecyl ester^(6)^ | AMK |
| **NO.** | **RT**  **min** | **Experical Formula** | **Proposal Ions** | **Experimental  Mass m/z** | **Theoretical Mass m/z** | **Mass Error (ppm)** | **MS2/MS3** | **Identification** | **Crude drug** |
| 226 | 29.83 | C_33_H_49_O_5_ | [M-H]^-^ | 525.35846 | 525.35745 | 1.920 | MS^2^: 507.36237, 481.45844, 479.39819, 465.36340, 463.37976, 355.20465  MS^3^[465.36340]: 447.35889, 421.48898, 405.28421, 355.46649, 295.27435 | Dehydropachymic acid (DPA)^(41)^ | PCW |
| 227 | 29.98 | C_25_H_47_O_11_S | [M-H]^-^ | 555.28375 | 555.28335 | 0.704 | MS^2^: 537.33215, 527.19171, 299.10162, 255.16750, 206.94096, 164.88426 | 3-hydroxy-2-(palmitoyloxy)propyl-6-deoxy 6-sulfohexopyranoside^(2)^ | LJH |
| 228 | 30.02 | C_33_H_51_O_5_ | [M-H]^-^ | 527.37408 | 527.3731 | 1.856 | MS^2^: 509.40070, 483.39362, 467.43542, 465.26843, 451.66022, 449.44745 | Pachymic acid (PA)^(41)^ | PCW |
| 229 | 30.15 | C_30_H_47_O_3_ | [M-H]^-^ | 455.35297 | 455.35197 | 0.998 | MS^2^: 437.18906, 434.97733, 415.12805, 407.37189 | Oleanolic acid^(46)^ | LJH SMB |
| 230 | 30.34 | C_18_H_31_O_2_ | [M-H]^-^ | 279.23288 | 279.23185 | 1.023 | MS^2^: 261.17123, 251.07458 | Linoleic acid^(3)^ | LJH SMB |
| 231 | 30.93 | C_16_H_31_O_2_ | [M-H]^-^ | 255.23271 | 255.23185 | 0.853 | MS^2^: 255.29187, 237.12975, 227.00484 | Palmitic acid^(3)^ | SMB |
| 232 | 32.94 | C_30_H_47_O_3_ | [M-H]^-^ | 455.35297 | 455.35197 | 0.998 | MS^2^: 437.38272, 409.38013, 353.40228 | Betulinic acid^(47)^ | LJH |
| 233 | 33.32 | C_36_H_67_N_6_O_6_ | [M+H]^+^ | 679.51099 | 679.51166 | -0.670 | MS^2^: 661.49091, 548.37921, 435.33447, 322.28650 | (Iso)Cyclohexaleucine | LJH |

“*”representes the compounds determined by the comparison of reference substance

1. Meng-Hua, Liu, and, Xin, Tong, and, et al. Rapid separation and identification of multiple constituents in traditional Chinese medicine formula Shenqi Fuzheng Injection by ultra-fast liquid chromatography combined with quadrupole-time-of-flight mass spectrometry. J Pharm Biomed Anal. 2013;74(2013):141-55.

2. Garran TA. A comparative study of Leonurus cardiaca and Leonurus japonicus [Doctor]: China Academy of Chinese Medical Sciences; 2020.

3. Yang ST, Wu X, Rui W, Guo J, Feng YF. UPLC/Q-TOF-MS analysis for identification of hydrophilic phenolics and lipophilic diterpenoids from Radix Salviae Miltiorrhizae. Acta Chromatographica. 2015;1(4):1-18.

4. Xiao M, Chen H, Shi Z, Feng Y, Rui W. Rapid and reliable method for analysis of raw and honey-processed astragalus by UPLC/ESI-Q-TOF-MS using HSS T3 columns. Analytical Methods. 2014;6(19):8045-54.

5. Chen L, Qi J, Chang YX, Zhu D, Yu B. Identification and determination of the major constituents in Traditional Chinese Medicinal formula Danggui-Shaoyao-San by HPLC-DAD-ESI-MS/MS. J Pharm Biomed Anal. 2009;50(2):127-37.

6. Sun X, Cui XB, Wen HM, Shan CX, Wang XZ, Kang A, et al. Influence of sulfur fumigation on the chemical profiles of Atractylodes macrocephala Koidz. evaluated by UFLC-QTOF-MS combined with multivariate statistical analysis. J Pharm Biomed Anal. 2017;141:19.

7. Xu W, Wu X, Huang MQ, Lin Y, Chen LD. Analysis on pharmacodynamic constituents in Gualou Guizhi Decoction by serum pharmacochemistry. Chinese Traditional & Herbal Drugs. 2017;48(10):2033-43.

8. Loureno ELB, Ferreira A, Pinto E, Yonamine M, Farsky SHP. On-Fiber Derivatization of SPME Extracts of Phenol, Hydroquinone and Catechol with GC-MS Detection. Chromatographia. 2006;63(3-4):175-9.

9. Jiang P, Ma Y, Gao Y, Li Z, Lian S, Xu Z, et al. A comprehensive evaluation of the metabolism of genipin-1-β-D-gentiobioside in vitro and in vivo by using HPLC-Q-TOF. J Agric Food Chem. 2016;64(27):5490-8.

10. Wang L, Halquist MS, Sweet DH. Simultaneous determination of gallic acid and gentisic acid in organic anion transporter expressing cells by liquid chromatography-tandem mass spectrometry. Journal of Chromatography B. 2013;937(2013):91-6.

11. Li T, Cao L, Tang H, Li J, Song Y. Liquid chromatography–three-dimensional mass spectrometry enables confirmative structural annotation of cistanoside F metabolites in rat. Journal of Chromatography B. 2021;1162(2021):122457.

12. Qiu XJ, Xi H, Chena ZQ, Ping R. Simultaneous Determination of 16 Chemical Constituents in the Traditional Chinese Medicinal Prescription Si-Ni-San and Chaihu-Shugan-San by Ultra Performance Liquid Chromatography Coupled with Photodiode Array Detection. Asian Journal of Chemistry. 2011;23(12):5301-7.

13. Huang X, Su S, Cui W, Liu P, Duan JA, Guo J, et al. Simultaneous determination of paeoniflorin, albiflorin, ferulic acid, tetrahydropalmatine, protopine, typhaneoside, senkyunolide I in Beagle dogs plasma by UPLC–MS/MS and its application to a pharmacokinetic study after Oral Administration of Shaofu Zhuy. J Chromatogr B Analyt Technol Biomed Life Sci. 2014;962:75-81.

14. Tuberoso CIG, Bifulco E, Jerkovi I, Caboni P, Floris I. Methyl Syringate: A Chemical Marker of Asphodel (Asphodelus microcarpus Salzm. et Viv.) Monofloral Honey. J Agric Food Chem. 2009;57(9):3895-900.

15. Ma XQ, Manleung A, Chan C, Su T, Li WD, Li SM, et al. UHPLC UHD Q-TOF MS/MS analysis of the impact of sulfur fumigation on the chemical profile of Codonopsis Radix (Dangshen). Analyst. 2013;139(2):505-16.

16. Sren, Damtoft, and, Sren, Brun, Hansen, et al. Iridoid glucosides from Melampyrum. Phytochemistry. 1984;23(10):2387-9.

17. Zhong R, Yu Y, Zheng Y, Chen W, Zhou G, Ding J, et al. A simple and selective UHPLC-MS/MS method for quantification of plantagoguanidinic acid in rat plasma and its application to a pharmacokinetic study. Biomed Chromatogr. 2017;31(8):e3929.

18. Manyuan, Wang, Shujun, Fu, Xinshi, Zhang, et al. LC-ESI-MS/MS Analysis and Pharmacokinetics of Plantainoside D Isolated from Chirita longgangensis var. hongyao, a Potential Anti-Hypertensive Active Component in Rats. Molecules. 2014;19(9):15103-15.

19. Jing Z, Xiao-Jie X, Wen X, Huang J, Da-yuan Z, Xiao-Hui Q. Rapid Characterization and Identification of Flavonoids in Radix Astragali by Ultra-High-Pressure Liquid Chromatography Coupled with Linear Ion Trap-Orbitrap Mass Spectrometry. J Chromatogr Sci. 2015;53(6):945.

20. Feng, Qiu, Zhaoxia, Li, Lang, He, et al. HPLC-ESI-MS/MS analysis and pharmacokinetics of luteoloside, a potential anticarcinogenic component isolated from Lonicera japonica, in beagle dogs. Biomed Chromatogr. 2013;27(3):311-7.

21. Jiang Y, Li SP, Wang YT, Chen XJ, Tu PF. Differentiation of Herba Cistanches by fingerprint with high-performance liquid chromatography-diode array detection-mass spectrometry. J Chromatogr A. 2009;1216(11):2156-62.

22. Liang W, Chen W, Wu L, Shi L, Qi Q, Cui Y, et al. Quality Evaluation and Chemical Markers Screening of Salvia miltiorrhiza Bge. (Danshen) Based on HPLC Fingerprints and HPLC-MSn Coupled with Chemometrics. Molecules. 2017;22(3):478.

23. Gao X, Sun WJ, Fu Q, Niu XF. Ultra-performance liquid chromatography coupled with electrospray ionization/quadrupole time-of-flight mass spectrometry for the rapid analysis of constituents in the traditional Chinese medical formula Danggui San. Journal of Separation Science. 2014;37(1-2):53-60.

24. Liu L, Cheng Y, Zhang H. Phytochemical analysis of anti-atherogenic constituents of Xue-Fu-Zhu-Yu-Tang using HPLC-DAD-ESI-MS. Chem Pharm Bull (Tokyo). 2004;52(11):1295-301.

25. Li Y, Gan L, Li GQ, Deng L, Deng Y. Pharmacokinetics of plantamajoside and acteoside from Plantago asiatica in rats by liquid chromatography-mass spectrometry. J Pharm Biomed Anal. 2014;89:251-6.

26. Washida K, Yamagaki T, Iwashita T, Nomoto K. Two New Galloylated Monoterpene Glycosides, 4-O-Galloylalbiflorin and 4′-O-Galloylpaeoniflorin, from the Roots of Paeonia lactiflora (Paeoniae Radix) Grown and Processed in Nara Prefecture, Japan. Cheminform. 2010;41(11):1150-2.

27. Othman MR, Othman R, Ismail AA, Hazni H, Awang K. High-Performance Liquid Chromatography Quadrupole Time-of-Flight Mass Spectrometry (HPLC-QTOFMS) Analysis on the Ethanol:Water (80:20) Extract of Lawsonia inermis Leaves. Sains Malaysiana. 2020;49(7):1597-613.

28. Li X, Xiao H, Liang X, Shi D, Liu J. LC-MS/MS determination of naringin, hesperidin and neohesperidin in rat serum after orally administrating the decoction of Bulpleurum falcatum L. and Fractus aurantii. J Pharm Biomed Anal. 2004;34(1):159-66.

29. Marchetti L, Pellati F, Graziosi R, Brighenti V, Pinetti D, Bertelli D. Identification and determination of bioactive phenylpropanoid glycosides of Aloysia polystachya (Griseb. et Moldenke) by HPLC-MS. J Pharm Biomed Anal. 2019;166:364-70.

30. Souza LMD, Cipriani TR, Serrato RV, Costa DED, Iacomini M, Gorin PAJ, et al. Analysis of flavonol glycoside isomers from leaves of Maytenus ilicifolia by offline and online high performance liquid chromatography-electrospray mass spectrometry. J Chromatogr A. 2008;1207(1-2):101-9.

31. Li HX, Ding MY, Yu JY. Separation and identification of the phthalic anhydride derivatives of Liqusticum Chuanxiong Hort by GC-MS, TLC, HPLC-DAD, and HPLC-MS. J Chromatogr Sci. 2002;40(3):156-61.

32. Dai X, Pang L, Zhang Z, Yang C, Li Y. Development of a sensitive LC–MS/MS method for quantification of coniferyl ferulate and its metabolite coniferyl alcohol in rat plasma: Application to a pharmacokinetic study. J Pharm Biomed Anal. 2017;146:201-5.

33. Senay, Simsek, Douglas, C., Doehlert. Oxygenated fatty acids isolated from wheat bran slurries. Int J Food Sci Nutr. 2014;65(7):803-8.

34. Yi H, Jia X, Liu X, Duan T, Chen H. DLLME Combined with GC–MS for the Determination of Methylparaben, Ethylparaben, Propylparaben and Butylparaben in Beverage Samples. Chromatographia. 2010;72(s3-4):351-5.

35. Yi T, Su D, Du Y, Li W, Hu L. Magnetic solid-phase extraction coupled with HPLC-Q-TOF-MS for rapid analysis of tyrosinase binders from San-Bai decoction by Box–Behnken statistical design. Rsc Advances. 2016;6(111).

36. . !!! INVALID CITATION !!! (36).

37. Jingze, Zhang, Xiao, Hu, Wenyuan, Gao, et al. Pharmacokinetic study on costunolide and dehydrocostuslactone after oral administration of traditional medicine Aucklandia lappa Decne. by LC/MS/MS. J Ethnopharmacol. 2014;151(1):191-7.

38. Hon PM, Wang ES, Lam SKM, Choy YM, Chi ML, Wong HNC. Preleoheterin and leoheterin, two labdane diterpenes from Leonurus heterophyllus. Phytochemistry. 1993;33(3):639-41.

39. Zhu L, Xiang C, Zhuang W, He J, Qin Y, Li P, et al. Screening the antiangiogenic constituents from salvia przewalskii maxim and quantitative analysis of them. Asian Journal of Chemistry. 2013;25(15):8517-21.

40. Giang PM, Son PT, Matsunami K, Otsuka H. New Bis-spirolabdane-Type Diterpenoids from Leonurus heterophyllus SW. Chem Pharm Bull (Tokyo). 2005;53(11):1475-9.

41. Wu LF, Wang KF, Xin M, Liang WY, Chen WJ, Shi L, et al. Screening and Analysis of the Potential Bioactive Components of Poria cocos (Schw.) Wolf by HPLC and HPLC-MSn with the Aid of Chemometrics. Molecules. 2016;21(2):227.

42. Chu C, Cai HX, Ren MT, Liu EH, Li P. Characterization of novel astragaloside malonates from Radix Astragali by HPLC with ESI quadrupole TOF MS. Journal of Separation Science. 2015;33(4-5):570-81.

43. Hradec J, Dufek P. Determination of cholesteryl 14-methylhexadecanoate in blood serum by reversed-phase high-performance liquid chromatography. Journal of Chromatography B Biomedical Applications. 1994;660(2):386.

44. Yang D, Qingyun MA, Cheng Z, Huang S, Minghe MO, Zhao Y. Isolation and Identification of Triterpenoids and Their Biocontrol Activity from the Peer of Poria cocos. Guizhou Agricultural Sciences. 2015.

45. Song-Lin YZXJ-DFLYZL. Evaluation of chemical consistency of triterpene acids in ethanol extracts of Poria and acetic ether extracts thereof by UPLC-QTOF-MS/MS with full scan and mimic MRM mode. Acta Pharmaceutica Sinica. 2019;54(01):130-7.

46. Zeng H, Su S, Xiang X, Sha X, Zhu Z, Wang Y, et al. Comparative Analysis of the Major Chemical Constituents in Salvia miltiorrhiza Roots, Stems, Leaves and Flowers during Different Growth Periods by UPLC-TQ-MS/MS and HPLC-ELSD Methods. Molecules. 2017;22(5):771.

47. Gowers GOF, Chee SM, Bell D, Suckling L, Kern M, Tew D, et al. Improved betulinic acid biosynthesis using synthetic yeast chromosome recombination and semi-automated rapid LC-MS screening. Nature Communications. 2020;11(1):868.
