# Supplementary material for: Integrated UPLC-MS and Network Pharmacology Approach to Explore the Active Components and the Potential Mechanism of Yiqi Huoxue Decoction for Treating Nephrotic Syndrome
Source: Front Pharmacol. 2022 Feb 24;12:775745. doi: 10.3389/fphar.2021.775745 (PMC8919777; doi:10.3389/fphar.2021.775745)
Supplement: Supplementary file 2 [file Table2.docx]

Table S2. Identification of compounds in rat plasma by UPLC-MS^n^.

| **No.** | **RT min** | **Proposal  Ions** | **Experical Formula** | **Experimental Mass m/z** | **Theoretical Mass m/z** | **Mass  Error** | **M^2^/MS^3^** | **Identification** |
| --- | --- | --- | --- | --- | --- | --- | --- | --- |
| 1* | 0.93 | [M+H]^+^ | C_5_H_14_ON | 104.10710 | 104.10699 | 0.109 | MS^2^: 103.85497, 86.00939, 59.73006, | Choline^(1)^ |
| 2* | 1.84 | [M-H]^-^ | C_5_H_6_NO_3_ | 128.03564 | 128.03421 | 1.420 | MS^2^: 127.91696, 83.87839 | Pyroglutamic acid^(1)^ |
| 3* | 2.06 | [M+H]^+^ | C_5_H_6_N_5_ | 136.06169 | 136.06177 | -0.082 | MS^2^: 135.87534, 118.79553, 93.78267 | Adenine^(1)^ |
| 4* | 2.06 | [M+H]^+^ | C_6_H_14_NO_2_ | 132.10196 | 132.10190 | 0.055 | MS^2^: 85.76086, 68.81328, 57.77438 | L-Isoleucine^(1)^ |
| 5* | 2.10 | [M+H]^+^ | C_9_H_12_NO_3_ | 182.08138 | 182.08116 | 0.210 | MS^2^: 164.87901, 135.97744 MS^3^[164.87901]: 146.77562, 122.85385, 118.76942 | Tyrosine^(1)^ |
| 6 | 2.10 | [M+H]^+^ | C_9_H_9_O_3_ | 165.05447 | 165.05462 | -0.151 | MS^2^: 146.82478, 122.93800, 118.73433 MS^3^[146.82478]: 118.78551, 90.78123 | 4-Hydroxycinnamic acid |
| 7* | 2.33 | [M+H]^+^ | C_6_H_14_NO_2_ | 132.10191 | 132.10190 | 0.005 | MS^2^: 131.81645, 113.77196, 85.78362 | Leucine^(1)^ |
| 8 | 3.36 | [M+H]^+^ | C_12_H_15_O_4_ | 223.09410 | 223.09648 | -2.385 | MS^2^: 224.91832,206.99998, 180.90407, 164.60556 | 4,7-Dihydroxy-3-butylphthalide^(2)^ |
| 9* | 4.29 | [M+H]^+^ | C_9_H_12_NO_2_ | 166.08632 | 166.08625 | 0.065 | MS^2^: 119.80794 | Phenylalanine^(1)^ |
| 10 | 4.29 | [M+H]^+^ | C_8_H_10_N | 120.08070 | 120.08077 | -0.076 | MS^2^: 119.82368, 102.92522, 92.80870 | 2-ethenyl-benzenamine |
| 11* | 4.78 | [M-H]^-^ | C_9_H_9_O_5_ | 197.04539 | 197.04444 | 0.940 | MS^2^: 178.86980, 152.97386, 122.86346 | Danshensu^(3)^ |
| 12* | 4.83 | [M-H]^-^ | C_16_H_21_O_10_ | 373.11386 | 373.11292 | 0.937 | MS^2^: 210.94466, 166.89185, 148.84869,122.83897 | Geniposidic acid^(4)^ |
| 13* | 5.01 | [M-H]^-^ | C_7_H_5_O_3_ | 137.02452 | 137.02332 | 1.199 | MS^2^: 136.77991, 108.68976, 92.82329 | Protocatechualdehyde |
| 14* | 5.36 | [M+H]^+^ | C_11_H_13_N_2_O_2_ | 205.09721 | 205.09715 | 0.056 | MS^2^: 187.91312, 159.01634, 146.98102, 131.84344 | Tryptophan^(1)^ |
| 15* | 5.67 | [M-H]^-^ | C_10_H_9_O_4_ | 193.05035 | 193.04955 | 0.815 | MS^2^: 177.88452, 148.98358, 133.87895 | Ferulic acid^(1)^ |
| 16* | 5.91 | [M+H]^+^ | C_9_H_7_O_3_ | 163.03920 | 163.03897 | 0.229 | MS^2^: 162.91995, 144.82370, 134.83873 MS^3^[144.82370]: 116.74153, 88.78174 | 4-Hydroxycoumarin |
| 17 | 6.22 | [M-H]^-^ | C_7_H_5_O_4_ | 153.01926 | 153.01823 | 1.025 | MS^2^: 108.91360, 94.99182 | Protocatechuic acid^[3]^ |
| 18 | 7.67 | [M-H]^-^ | C_9_H_9_O_5_ | 197.04546 | 197.04444 | 1.010 | MS^2^: 181.80382, 123.76682 | Syringic acid |
| **No.** | **RT min** | **Proposal  Ions** | **Experical Formula** | **Experimental Mass m/z** | **Theoretical Mass m/z** | **Mass  Error** | **M2/MS3** | **Identification** |
| 19* | 8.13 | [M-H]^-^ | C_22_H_25_O_10_ | 449.14514 | 449.14422 | 2.041 | MS^2^: 327.14380, 309.18292, 164.92078 | (6R,9S)-3-Oxo-a-ionolb- D-glucopyranosidesulfate^(5)^ |
| 20* | 8.13 | [M-H]^-^ | C_19_H_29_O_10_S | 449.14551 | 449.14759 | -2.084 | MS^2^: 327.09210, 309.15057, 164.88855 | Melampyroside^(6)^ |
| 21* | 8.35 | [M+H]^+^ | C_14_H_21_N_3_O_5_ | 312.15482 | 312.15539 | -0.577 | MS^2^: 294.99539, 180.87286, 113.83076 | Leonurine^(7)^ |
| 22* | 8.38 | [M+COOH]^-^ | C_24_H_29_O_13_ | 525.16034 | 525.16026 | 0.073 | MS^2^: 478.92010, 449.13641, 327.09467 | Paeoniflorin^(8)^ |
| 23* | 9.26 | [M+H]^+^ | C_11_H_20_N_3_O_2_ | 226.15468 | 226.155 | -0.323 | MS^2^: 207.98775, 180.10121, 83.74562 | Plantagoguanidinic acid^(9)^ |
| 24* | 9.57 | [M+H]^+^ | C_22_H_23_O_10_ | 447.12842 | 447.12857 | -0.153 | MS^2^: 285.06720 MS^3^[285.06720]: 269.96832, 252.90375, 224.98459, 136.73813 | Calycosin-7-O-β-D-glucoside^(10)^ |
| 25* | 9.92 | [M-H]^-^ | C_27_H_29_O_16_ | 609.14532 | 609.14501 | 0.516 | MS^2^: 300.99994, 300.02588, 271.03522 | Rutin^(1)^ |
| 26 | 10.64 | [M+H]^+^ | C_30_H_56_N_5_O_5_ | 566.42621 | 566.42759 | -1.386 | MS^2^: 548.42084, 435.36401, 322.18591, 208.96283 | Cyclopenta (iso)leucine |
| 27 | 11.07 | [M+H]^+^ | C_12_H_17_O_3_ | 209.11736 | 209.11722 | 0.139 | MS^2^: 208.98354, 190.98758, 162.92674, 152.82274 | Senkyunolide G^[24]^ |
| 28 | 12.46 | [M+H]^+^ | C_36_H_67_N_6_O_6_ | 679.51099 | 679.51166 | -0.980 | MS^2^: 661.47943, 548.42059, 435.31854, 322.12280, 209.08578 | Cyclohexa (iso)leucine |
| 29* | 12.93 | [M-H]^-^ | C_28_H_32_O_15_ | 609.18188 | 609.18139 | 1.007 | MS^2^: 300.96039, 286.08490 MS^3^[300.96039]: 285.98416, 268.01797, 257.00543, 241.92694 | Hesperidin^(11)^ |
| 30* | 13.62 | [M-H]^-^ | C_28_H_32_O_15_ | 609.18188 | 609.18139 | 1.302 | MS^2^: 301.00714, 285.99182 MS^3^[301.00714]: 285.96609, 268.01086, 257.03241, 241.95950 | Neohesperidin^(11)^ |
| 31 | 15.63 | [M-H]^-^ | C_12_H_13_O_4_ | 221.08177 | 221.08083 | 0.935 | MS^2^: 176.94601 MS^3^[176.94601]: 146.84616, 134.91173, 119.79230, 91.82375 | Diethyl phthalate |
| 32 | 16.8 | [M-H]^-^ | C_23_H_23_O_11_ | 475.12512 | 475.12348 | 1.632 | MS^2^: 456.97397, 299.07635, 174.82127 | Plantasioside |
| 33* | 17.24 | [M+H]^+^ | C_16_H_13_O_5_ | 285.07568 | 285.07575 | -0.070 | MS^2^: 269.91565, 252.97833, 224.90387, 136.87979 | Calycosin^(10)^ |
| 34 | 17.26 | [M-H]^-^ | C_23_H_25_O_11_ | 477.14056 | 477.13913 | 1.422 | MS^2^: 408.87421, 301.04102, 174.71718 | plantainoside B |
| **No.** | **RT min** | **Proposal  Ions** | **Experical Formula** | **Experimental Mass m/z** | **Theoretical Mass m/z** | **Mass  Error** | **M2/MS3** | **Identification** |
| 35* | 17.32 | [M-H]^-^ | C_15_H_9_O_6_ | 285.04044 | 285.03936 | 0.555 | MS^2^: 284.99921, 240.89581, 216.97528, 174.99593, 150.75604 | Luteolin^(12)^ |
| 36 | 17.34 | [M-H]^-^ | C_23_H_25_O_11_ | 477.14078 | 477.13913 | 1.642 | MS^2^: 408.87265, 301.04153, 175.01697 | Calceorioside B |
| 37* | 20.05 | [M-H]^-^ | C_15_H_9_O_5_ | 269.04520 | 269.04444 | 0.750 | MS^2^: 248.95686, 241.02834, 224.95999, 182.80550, 148.85371 | Apigenin^(10)^ |
| 37* | 20.07 | [M+H]^+^ | C_15_H_11_O_5_ | 271.05994 | 271.06009 | -0.160 | MS^2^: 270.99731, 243.02495, 224.90265, 152.82750, 134.97874 | Apigenin^(10)^ |
| 38 | 20.52 | [M-H]^-^ | C_15_H_9_O_6_ | 285.04037 | 285.03936 | 1.006 | MS^2^: 284.92410, 264.87445, 256.92914, 240.94769, 150.91664 | Kaempferol^(13)^ |
| 39 | 21.54 | [M-H]^-^ | C_17_H_13_O_6_ | 313.07153 | 313.07066 | 0.865 | MS^2^: 297.83539, 268.92551, 225.00107, 120.86797 | Salvianolic acid F^(14)^ |
| 40 | 21.84 | [M-H]^-^ | C_16_H_11_O_4_ | 267.06593 | 267.06518 | 0.735 | MS^2^: 252.05936 | Formononetin^(1)^ |
| 41 | 23.16 | [M+H]^+^ | C_16_H_13_O_5_ | 285.07529 | 285.07575 | -0.460 | MS^2^: 270.02197 MS^3^[270.02197]: 251.96974, 241.92352, 223.80623, 178.95102 | Wogonin^(1)^ |
| 41 | 23.16 | [M-H]^-^ | C_16_H_11_O_5_ | 283.06100 | 283.06009 | 0.900 | MS^2^: 267.91516, 242.97446, 166.94473 | Wogonin^(1)^ |
| 42 | 25.04 | [M-H]- | C19H21O4 | 313.14426 | 313.14343 | 0.824 | MS^2^: 292.96503, 269.13028, 241.04068, 226.00989, 183.14142 | Neocryptotanshinone^[50]^ |
| 43 | 25.24 | [M+H]+ | C19H19O4 | 311.12793 | 311.12778 | 0.144 | MS2: 283.11206 MS^3^[283.11206]: 264.98773, 254.07599, 241.01566, 237.06198 | Hydroxytanshinone IIA^(14)^ |
| 44 | 25.28 | [M+H]+ | C19H19O4 | 311.12817 | 311.12778 | 0.384 | MS2: 282.99210 MS^3^[282.99210]: 265.02911, 240.99643, 237.01489, 223.04358 | Hydroxycryptotanshinone^(14)^ |
| 45 | 27.19 | [M+H]^+^ | C_25_H_51_N_3_O_6_S | 522.35364 | 522.35713 | -0.123 | MS^2^: 504.35309, 183.90768 | N,N,N-tributyl-1-butanaminium(2S,3S)-2-methyl-3-([[(2-methyl-2-propanyl)oxy]carbonyl]amino)-4-oxo-1-azetidinesulfonate^(15)^ |
| 46 | 27.34 | [M-H]^-^ | C_30_H_45_O_5_ | 485.32761 | 485.32615 | 1.459 | MS^2^: 464.94211, 439.38687, 411.31262, 383.28577, 369.23242 | PoriacosonesA/PoriacosonesB |
| 47* | 27.48 | [M+H]^+^ | C_15_H_19_O_2_ | 231.13806 | 231.13795 | 0.104 | MS^2^: 212.89125, 202.96411, 184.98637, 156.91467, 142.84723 | Atractylenolide I^(1)^ |
| 48* | 28.86 | [M+H]^+^ | C_19_H_19_O_3_ | 295.13312 | 295.13287 | 0.249 | MS^2^: 277.02859, 253.12329, 248.96436, 235.01224 | Tanshinone IIA^(3)^ |
| 49 | 29.74 | [M-H]^-^ | C_16_H_31_O_3_ | 271.22769 | 271.22677 | 0.919 | MS^2^: 225.13847, 204.87167, 154.76984, 88.75327 MS^3^[225.13847]: 221.09474, 203.11285, 197.16194, 82.70636 | 3-hydroxy-propionic acid tridecyl ester^(15)^ |
|  |  |  |  |  |  |  |  |  |
| **No.** | **RT min** | **Proposal  Ions** | **Experical Formula** | **Experimental Mass m/z** | **Theoretical Mass m/z** | **Mass  Error** | **M2/MS3** | **Identification** |
| 50 | 30.23 | [M-H]^-^ | C_30_H_47_O_5_ | 487.34302 | 487.34180 | 1.219 | MS^2^: 443.43552 MS^3^[443.43552]: 425.35574, 399.46808, 357.27618, 341.28702, 279.17773 | 3α,16α,26-Trihydroxylanosta-8,24-dien-21-oic acid |

“*” indicates that the compounds exist in YQHXD and rat plasma simultaneously.

1. Meng-Hua, Liu, and, Xin, Tong, and, et al. Rapid separation and identification of multiple constituents in traditional Chinese medicine formula Shenqi Fuzheng Injection by ultra-fast liquid chromatography combined with quadrupole-time-of-flight mass spectrometry. J Pharm Biomed Anal. 2013;74(2013):141-55.

2. Li HX, Ding MY, Yu JY. Separation and identification of the phthalic anhydride derivatives of Liqusticum Chuanxiong Hort by GC-MS, TLC, HPLC-DAD, and HPLC-MS. J Chromatogr Sci. 2002;40(3):156-61.

3. Yang ST, Wu X, Rui W, Guo J, Feng YF. UPLC/Q-TOF-MS analysis for identification of hydrophilic phenolics and lipophilic diterpenoids from Radix Salviae Miltiorrhizae. Acta Chromatographica. 2015;1(4):1-18.

4. Jiang P, Ma Y, Gao Y, Li Z, Lian S, Xu Z, et al. A comprehensive evaluation of the metabolism of genipin-1-β-D-gentiobioside in vitro and in vivo by using HPLC-Q-TOF. J Agric Food Chem. 2016;64(27):5490-8.

5. Ma XQ, Manleung A, Chan C, Su T, Li WD, Li SM, et al. UHPLC UHD Q-TOF MS/MS analysis of the impact of sulfur fumigation on the chemical profile of Codonopsis Radix (Dangshen). Analyst. 2013;139(2):505-16.

6. Sren, Damtoft, and, Sren, Brun, Hansen, et al. Iridoid glucosides from Melampyrum. Phytochemistry. 1984;23(10):2387-9.

7. Garran TA. A comparative study of Leonurus cardiaca and Leonurus japonicus [Doctor]: China Academy of Chinese Medical Sciences; 2020.

8. Chen L, Qi J, Chang YX, Zhu D, Yu B. Identification and determination of the major constituents in Traditional Chinese Medicinal formula Danggui-Shaoyao-San by HPLC-DAD-ESI-MS/MS. J Pharm Biomed Anal. 2009;50(2):127-37.

9. Zhong R, Yu Y, Zheng Y, Chen W, Zhou G, Ding J, et al. A simple and selective UHPLC-MS/MS method for quantification of plantagoguanidinic acid in rat plasma and its application to a pharmacokinetic study. Biomed Chromatogr. 2017;31(8):e3929.

10. Jing Z, Xiao-Jie X, Wen X, Huang J, Da-yuan Z, Xiao-Hui Q. Rapid Characterization and Identification of Flavonoids in Radix Astragali by Ultra-High-Pressure Liquid Chromatography Coupled with Linear Ion Trap-Orbitrap Mass Spectrometry. J Chromatogr Sci. 2015;53(6):945.

11. Li X, Xiao H, Liang X, Shi D, Liu J. LC-MS/MS determination of naringin, hesperidin and neohesperidin in rat serum after orally administrating the decoction of Bulpleurum falcatum L. and Fractus aurantii. J Pharm Biomed Anal. 2004;34(1):159-66.

12. Cheruvu HS, Yadav NK, Valicherla GR, Arya RK, Hussain Z, Sharma C, et al. LC-MS/MS method for the simultaneous quantification of luteolin, wedelolactone and apigenin in mice plasma using hansen solubility parameters for liquid-liquid extraction: Application to pharmacokinetics of Eclipta alba chloroform fraction. J Chromatogr B 2018;1081-1082:76-86.

13. Souza LMD, Cipriani TR, Serrato RV, Costa DED, Iacomini M, Gorin PAJ, et al. Analysis of flavonol glycoside isomers from leaves of Maytenus ilicifolia by offline and online high performance liquid chromatography-electrospray mass spectrometry. J Chromatogr A. 2008;1207(1-2):101-9.

14. Liang W, Chen W, Wu L, Shi L, Qi Q, Cui Y, et al. Quality Evaluation and Chemical Markers Screening of Salvia miltiorrhiza Bge. (Danshen) Based on HPLC Fingerprints and HPLC-MSn Coupled with Chemometrics. Molecules. 2017;22(3):478.

15. Sun X, Cui XB, Wen HM, Shan CX, Wang XZ, Kang A, et al. Influence of sulfur fumigation on the chemical profiles of Atractylodes macrocephala Koidz. evaluated by UFLC-QTOF-MS combined with multivariate statistical analysis. J Pharm Biomed Anal. 2017;141:19.
